# Optimization of Indole- and Pyrazole-fused Glycyrrhetinic Acid Derivatives as Potent PTP1B Inhibitors: In Silico, In Vitro, In Vivo, and Metabolomic Studies

**DOI:** 10.1021/acsbiomedchemau.5c00164

**Published:** 2025-10-31

**Authors:** Mitzi López-Sánchez, Hannya Mendoza-Mota, Ledy De-la-Cruz-Martínez, Félix Matadamas-Martínez, Diana Laura Torres-Chacón, Rosendo Martínez-Arellano, Juan Francisco Palacios-Espinosa, Jaime Pérez-Villanueva, Martin González-Andrade, José Carlos Páez-Franco, Julio César Almanza-Pérez, Francisco Cortés-Benítez

**Affiliations:** a Maestría y Doctorado en Ciencias Farmacéuticas, División de Ciencias Biológicas y de la Salud, Universidad Autónoma Metropolitana − Unidad Xochimilco, Ciudad de México 04960, Mexico; b Laboratorio de Síntesis y Aislamiento de Sustancias Bioactivas, Departamento de Sistemas Biológicos, División de Ciencias Biológicas y de la Salud, Universidad Autónoma Metropolitana − Unidad Xochimilco, Ciudad de México 04960, Mexico; c Laboratorio de Biosensores y Modelaje Molecular, Departamento de Bioquímica, Facultad de Medicina, Universidad Nacional Autónoma de México, Ciudad de México 04510, Mexico; d Red de Apoyo a la Investigación, Universidad Nacional Autónoma de México e Instituto Nacional de Ciencias Médicas y Nutrición Salvador Zubirán, Ciudad de México 14080, Mexico; e Unidad de Investigación Médica en Enfermedades Infecciosas y Parasitarias, UMAE Hospital de Pediatría, Centro Médico Nacional Siglo XXI, 37767 Instituto Mexicano del Seguro Social, Ciudad de México 06720, Mexico; f Laboratorio de Farmacología, Departamento de Ciencias de la Salud, División de Ciencias Biológicas y de la Salud, Universidad Autónoma Metropolitana - Unidad Iztapalapa, Ciudad de México 09340, Mexico

**Keywords:** type 2 diabetes, protein tyrosine phosphatase 1B, glycyrrhetinic acid, molecular docking, untargeted metabolomics

## Abstract

Protein tyrosine phosphatase 1B (PTP1B) is a crucial enzyme involved in regulating insulin and leptin signaling pathways, making it a promising target for treating type 2 diabetes. In this study, we synthesized 14 derivatives of indole- and pyrazole-fused glycyrrhetinic acid (GA) and evaluated their effects on PTP1B, using both its long (*h*PTP1B_1–400_) and short (*h*PTP1B_1–285_) forms. We analyzed enzymatic kinetics and selectivity over T-cell protein tyrosine phosphatase (TCPTP). Molecular docking and molecular dynamics simulations were performed to understand the binding mode of the compounds within PTP1B. Untargeted metabolomics, using gas chromatography–mass spectrometry, assessed metabolic changes caused by the most potent PTP1B inhibitors in HepG2 cells. We also evaluated these inhibitors *in vivo* to determine their effects on insulin sensitivity through the insulin tolerance test (ITT) in streptozotocin (STZ)-induced diabetic mice. Two compounds, **4b** (indole-fused) and **5g** (pyrazole-fused), showed greater potency against the long form of PTP1B compared to the short form, indicating that both compounds preferentially bind to the disordered C-terminal region of PTP1B. Molecular docking and molecular dynamics studies supported this finding. Furthermore, enzymatic kinetics revealed that compounds **4b** and **5g** function as uncompetitive inhibitors of PTP1B, with *K*_i_ values of 0.32 and 0.72 μM, respectively. Notably, both GA derivatives exhibited more pronounced inhibition of PTP1B compared to the well-established inhibitors ursolic acid and Ertiprotafib, while also demonstrating selectivity over TCPTP. Metabolomic analysis revealed that these compounds increased pantothenic acid and glycine levels, while decreasing glucose and fatty acid levels in HepG2 cells, suggesting enhanced glycolysis and reduced lipogenesis. Both compounds exhibited low cytotoxicity in HFF-1 cells and significantly reduced glucose levels in the ITT in STZ-induced diabetic mice, outperforming the insulin-sensitizing drug Pioglitazone.

## Graphical abstract

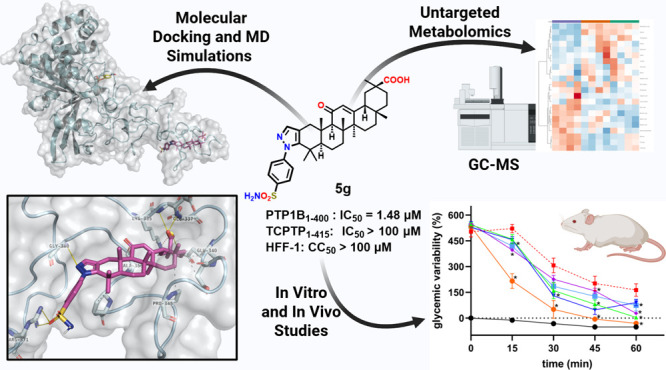

## Introduction

According to the World Health Organization (WHO), diabetes is a chronic metabolic disease characterized by elevated blood glucose levels, which can lead to significant damage over time to the heart, blood vessels, eyes, kidneys, and nerves.^1^ Recently, the International Diabetes Federation has reported that 589 million adults aged 20 to 79 years are currently living with diabetes, which represents 11.1% of the global population in this age group. It is projected that by 2050, this number will rise to 852.5 million people, marking an increase of 13.0%.^2^ Currently, the most common form of diabetes is type 2 diabetes mellitus (T2DM), accounting for 90% of cases, with contributing factors including urbanization, an aging population, sedentary lifestyles, and increasing rates of obesity and overweight individuals. It is important to note that complications associated with this disease, especially concerning kidney and cardiovascular failure, resulted in approximately 3.4 million deaths globally. This corresponds to 9.3% of global deaths from all causes.

The development of T2DM is a complex process; however, various molecular pathways and targets have been identified, facilitating the development of new treatments.^3–6^ The main medication categories include secretagogues (such as sulfonylureas and glinides like Glibenclamide and Repaglinide), insulin mimickers (such as DPP–4 inhibitors or gliptins, including Sitagliptin and Linagliptin), insulin sensitizers (PPAR−γ agonists like Pioglitazone and Rosiglitazone), starch blockers (α–glucosidase inhibitors such as Acarbose and Voglibose), and renal glucose reabsorption inhibitors (SGLT2 inhibitors such as Canagliflozin and Dapagliflozin). While these medications enhance blood glucose management and aid in diabetes control, they can also have various side effects, ranging from mild complications to severe conditions,^7–11^ including gastrointestinal issues (like nausea and diarrhea), dry mouth, headache, insomnia, anemia, neuropathy, hypoglycemia, bladder cancer, and increased risk of cardiovascular problems. Therefore, it is essential to create treatments for T2DM that are both safe and effective while also addressing and potentially reversing its complications.

Another interesting molecular target is Protein Tyrosine Phosphatase 1B (PTP1B), a major nonreceptor protein tyrosine phosphatase (PTP) encoded by the *PTPN1* gene.^12,13^ Its structure consists of three domains: an N-terminal catalytic domain (1–300), a regulatory domain (301–400), and a C-terminal domain (401–435) that guides the enzyme to the endoplasmic reticulum (ER) membrane.^14,15^ Its involvement in downregulating the canonical insulin and leptin signaling pathways is well-established. PTP1B is responsible for catalyzing the dephosphorylation of phosphotyrosine (pTyr) residues on the activated insulin receptor subunit β (IRb) as well as on insulin receptor substrates 1 and 2 (IRS-1, IRS-2), suppressing downstream phosphatidylinositol (PI3K)/AKT signaling.^15,16^ Additionally, it dephosphorylates the leptin receptor (LepR) and Janus kinase (JAK2), which in turn inactivates the signal transducer and activator of transcription 3 (STAT3). This mechanism plays a key role in regulating the expression of genes such as POMC and SOCS3, both of which are involved in WAT browning and energy expenditure.^17,18^ The overexpression of PTP1B interferes with these signaling pathways, which can lead to the development of various conditions and diseases, including several types of cancer, especially breast and pancreatic cancer, cardiovascular diseases, fatty liver, and senescence.^19–23^ Additionally, its role has been recently identified in mental health conditions such as schizophrenia, Alzheimer’s disease, Rett syndrome, and major depressive disorder.^24–26^

In this context, elevated levels of PTP1B, particularly in the hypothalamus, are associated with insulin and leptin resistance. Mice lacking PTP1B exhibit improved glucose regulation, reduced weight gain, and altered energy expenditure.^27,28^ Therefore, PTP1B inhibitors function as insulin mimickers and agents that sensitize insulin and leptin receptors,^29^ thus presenting a dual advantage in tackling both T2DM and obesity, which is a noteworthy benefit over current therapies. However, developing PTP1B inhibitors remains a challenge. Many of these inhibitors contain a highly negatively charged group that interacts with the catalytic site, negatively affecting their pharmacokinetic properties.^30^ Additionally, the highly conserved catalytic domain within the PTP family makes these inhibitors less selective, increasing their potential toxicity.^31,32^ As a result, there are currently no approved antidiabetic drugs that target PTP1B activity.^33^

Our research group recently reported the synthesis and assessment of indole- and pyrazole-fused Glycyrrhetinic acid (GA) derivatives (Figure 1).^34^ These compounds were tested for their ability to inhibit the formation of *p*-nitrophenol from *p*-nitrophenylphosphate (pNPP) in the presence of PTP1B. It was found that fusing these heterocycles to the GA backbone led to more potent PTP1B inhibitors, exhibiting 6- to 25-fold greater potency than GA. Similarly, they demonstrated superior potency compared to known PTP1B inhibitors such as ursolic acid and claramine. One of these compounds also displayed better activity than Suramin. Following this, the most potent compounds from the study (FC-114 and FC-122) (Figure 1) were tested in STZ-induced diabetic rats. The findings of this study indicated that both compounds had superior antihyperglycemic effects compared to glibenclamide and acarbose, but did not outperform pioglitazone.^35^ We hypothesized that this might be due to the high lipophilicity of both molecules, which could have negatively impacted their oral absorption. Therefore, herein, we present the synthesis of novel indole and *N*-phenylpyrazole derivatives of GA, featuring more polar substituents such as CN, COOH, CONH_2_, and SO_2_NH_2_ groups (Figure 1). Additionally, recognizing the significance of fluorinated semisynthetic derivatives in PTP1B inhibition,^34,36,37^ we also proposed the synthesis of compounds containing OCF_3_, SCF_3_, and OCF_2_H groups. A total of 14 new derivatives from both series were evaluated for their inhibitory activity against the long form of the PTP1B enzyme (*h*PTP1B_1–400_), with their potency compared to that of well-established PTP1B inhibitors, including ursolic acid and Ertiprotafib. Furthermore, we conducted enzymatic kinetics analyses on the most promising compounds to ascertain their mode of inhibition on PTP1B.

**Figure 1:**
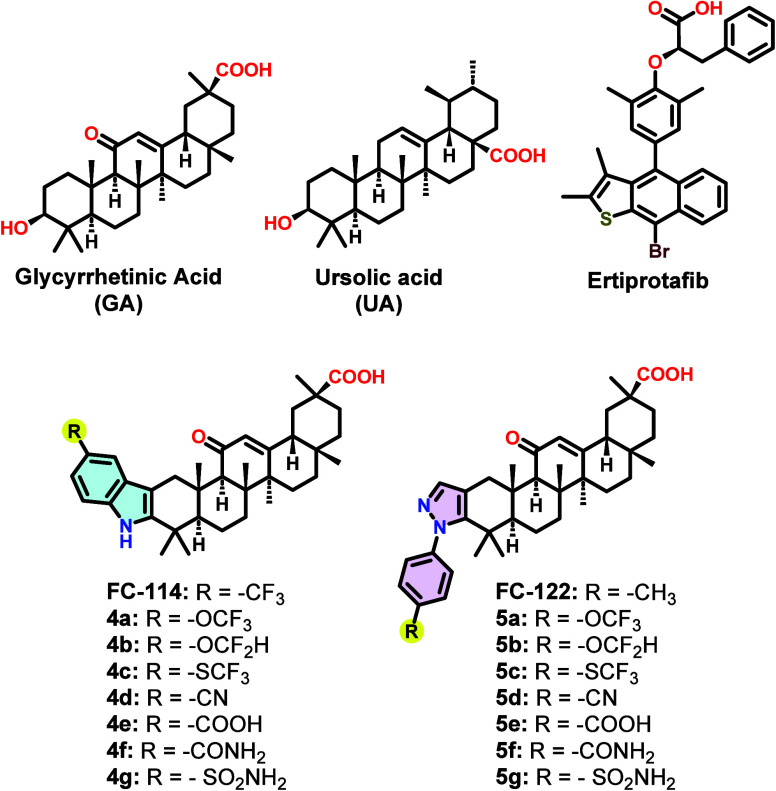
Structures of GA derivatives **4a**–**g** and **5a**–**g**, glycyrrhetinic acid, ursolic acid, and Ertiprotafib.

Given the importance of selectivity in developing PTP1B inhibitors, we assessed the ability of the compounds to inhibit the TCPTP enzyme, which shares 74% of structural homology with PTP1B at the catalytic site.^38,39^ To better understand the binding modes of the synthesized compounds to PTP1B, we performed molecular docking studies along with molecular dynamics simulations (MDS). The top candidates identified through enzymatic assays were tested to activate the phosphorylation of key proteins in the insulin and leptin signaling pathways in insulin-resistant HepG2 cells.

Moreover, metabolomics studies were conducted on HepG2 cells treated with the leading candidates to observe the metabolic changes induced by these compounds. To evaluate the potential toxicity of the synthesized molecules, we performed a cell viability assay in HFF-1 cell line. Finally, the most promising compounds were tested on STZ-induced diabetic CD-1 mice to assess their capability to reduce insulin resistance.

## Results and Discussion

### Synthesis

GA derivatives **4a**–**g** and **5a**–**g** were synthesized following the procedure outlined in Scheme 1. The synthesis began with Glycyrrhizin ammonium salt as the raw material. This triterpene was hydrolyzed in a hydrochloric acid solution, resulting in GA at a high yield of 85%. Subsequently, the carbinol at the C3 position of GA underwent oxidation via the Jones reaction, producing 3-oxoglycyrrhetinic acid (**2**) with a high yield (95%). Later, the Fischer indole reaction was employed to react this intermediate with various *p*-phenylhydrazines, yielding the indole-fused GA derivatives (**4a–g**) with yields ranging from 26% to 67%.

**Scheme 1:**
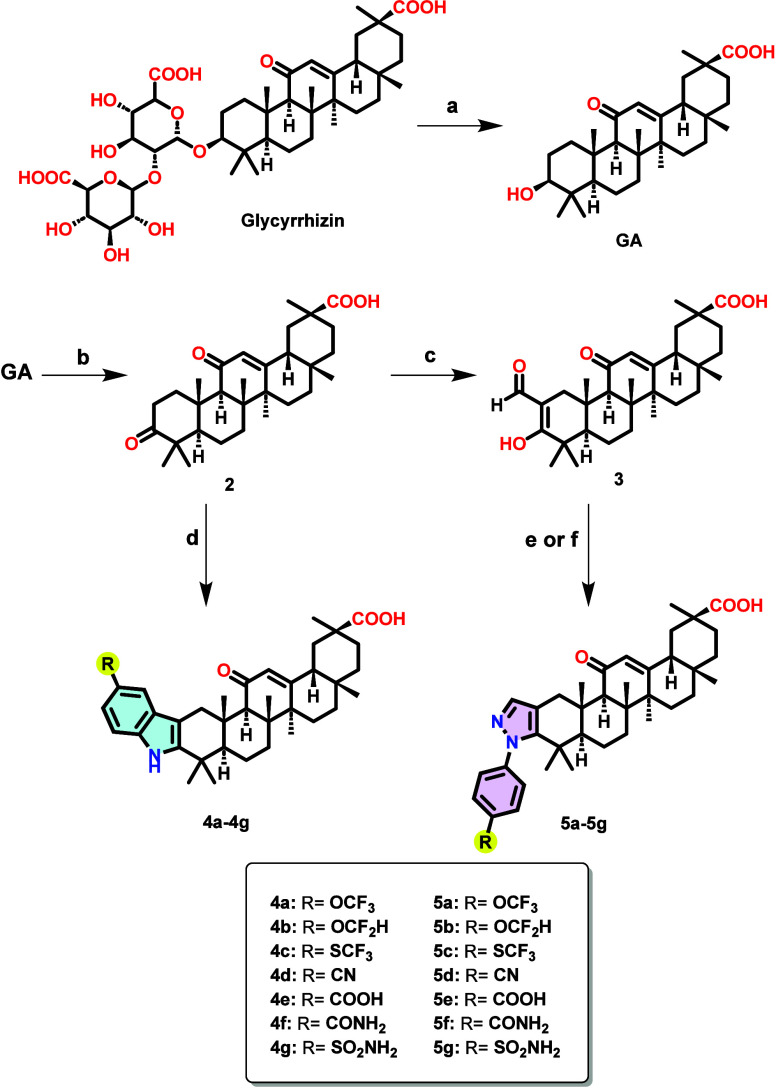
Reagents and Conditions for the Synthesis of Compounds **4a**–**g** and **5a**–**g** ^a^

a (a) HCl 1 M, reflux 18 h; (b) CrO_3_/H_2_SO_4_, THF 0 °C to r.t., 2 h; (c) ethyl formate, NaH, THF, r.t., 18 h; (d) Ar*-*NHNH_2_ hydrochloride, AcOH, reflux; (e) Ar-NHNH_2_ hydrochloride, EtOH, 80°C 2–12 h; (f) Ar-NHNH_2_ hydrochloride, DMF, r.t. 4–6 h.

In a parallel approach, intermediate **2** was reacted via Claisen condensation with ethyl formate and NaH to yield intermediate **3**, which was then reacted with *p*-phenylhydrazines to produce the pyrazole-fused GA derivatives (**5a–g**). Refluxing ethanol served as the initial solvent for synthesizing these compounds, resulting in yields ranging from 15% to 33%. However, we observed nearly a 2-fold increase in yield (up to 69%) when we replaced ethanol with dimethylformamide (DMF). Moreover, using DMF enabled the reaction to proceed efficiently at room temperature, while also decreasing the formation of byproducts. This allowed for easier purification through recrystallization, rather than using a chromatography column.

The structure of the final compounds was confirmed through ^1^H and ^13^C NMR (Supporting Information Figures S1–S28), as well as ESI-MS mass spectrometry (Supporting Information Figures S29–S42). In previous research, we provided a comprehensive characterization of the carbon and hydrogen signals in NMR for an indole and a *N*-phenylpyrazole derivative of GA.^34^ Building on this study, we observed very similar signals for the synthesized compounds. For instance, there are seven distinct signals integrating for the three hydrogens associated with the methyl groups 23-CH_3_, 24-CH_3_, 25-CH_3_, 26-CH_3_, 27-CH_3_, 28-CH_3_, and 29-CH_3_, appearing at approximate chemical shifts of 1.4, 1.3, and 1.2 ppm (as three separate signals), along with signals at 1.1 and 0.8 ppm. Both the indole and pyrazole derivatives of GA exhibit two doublet signals (*J* = 15.51 Hz) at approximately 3.7 and 2.2 ppm, corresponding to the diastereotopic hydrogens of the 1-CH_2_ group. The chemical shifts of these signals indicate the fusion of the heterocycle to the C2 and C3 positions of the triterpene. Additionally, in all final compounds, there is a single signal integrating for one hydrogen corresponding to the vinyl proton of 12-CH position. In the ^13^C NMR spectrum, characteristic signals of the triterpene skeleton were observed at approximately 199, 177, and 170 ppm, corresponding to the positions C12, 30-COOH, and C13, respectively.

The presence of the indole ring in the **4a–g** derivatives is supported by four signals each one integrating for one hydrogen in ^1^H NMR spectra: the first is a doublet of doublets (*J* = 1.44 and 8.67 Hz) at a chemical shift of 6.9 to 7.6 ppm, the second is a singlet observed at approximately 7.2 to 7.4 ppm, and the third is a doublet (*J* = 8.65 Hz) ranging from 7.2 to 7.9 ppm. The fourth consists of a broad singlet with variable chemical shifts ranging from 10.3 to 11.2 ppm, depending on the substituent at position 5 of the indole; this signal is attributed to the NH proton of the heterocycle.

In the ^1^H NMR spectra of the pyrazole derivatives of GA, the presence of a benzene ring attached to the N1 position of the pyrazole is indicated by two doublet signals (*J* = 9.1 or 8.5 Hz) located between 7.3 and 7.9 ppm, which integrate for two hydrogens and suggest a *para*-substituted pattern. Additionally, a single signal integrating for one hydrogen appears between 7.3 and 7.5 ppm, corresponding to the 3-CH of the pyrazole ring. In the ^13^C NMR spectrum, two intense signals are detected between 129 and 134 ppm, representing the four methine (CH) carbons of the aromatic ring. The C3 carbon of the pyrazole is observed around 138 ppm.

The various functional groups attached to the benzenoid ring of the indole and *N*-phenylpyrazole can also be confirmed by NMR spectroscopy. The trifluoromethoxy and trifluoromethylthio groups are detected as a quadruplet signal (*J* = 250 Hz) at 120 and 126 ppm, respectively, in the ^13^C NMR. Meanwhile, the difluoromethoxy group appears as a triplet signal at 119 ppm (*J* = 256 Hz) in the ^13^C NMR and at 7.1 ppm (*J* = 75 Hz) in the ^1^H NMR. The cyano group for compounds **4d** and **5d** is identified at 99.8 and 114 ppm, respectively, in their ^13^C NMR spectra. In terms of the carboxy group (−COOH) attached to the aromatic ring of compounds **4e** and **5e**, this group is located at 168.5 and 166.5 ppm, respectively, while for compounds **4f** and **5f**, the carboxamide is seen at 169.3 and 167.1 ppm, respectively. In the ^1^H NMR spectra, the carboxamide protons (−CONH_2_) of **4f** and **5f** appear as two separate signals that exchange with D_2_O at 6.9 and 7.9 ppm for compound **4f**, while for compound **5f**, this signal overlaps with the benzenoid protons at 7.5 ppm. Lastly, the protons of the sulfonamide group (−SO_2_NH_2_) for compounds **4g** and **5g** were assigned to a single broad signal integrating for two hydrogens, located at 7.0 and 7.5 ppm, respectively, in the ^1^H NMR.

### Biological Evaluation

#### PTP1B Inhibitory Activity

The inhibitory activity of PTP1B was assessed using the long form of the PTP1B enzyme (*h*PTP1B_1–400_). This enzyme comprises a highly conserved N-terminal catalytic domain, known as the PTP domain (PTP1B_1–300_), in addition to an intrinsically disordered regulatory domain (PTP1B_301–400_). Competitive, bidentate, and allosteric inhibitors primarily target the catalytic domain, while the regulatory domain has been proposed as an alternative site for allosteric inhibition.^14^

The synthesized compounds (**4a**–**g** and **5a**–**g**), along with reference inhibitors (GA, Ursolic acid, Ertiprotafib, FC-114, and FC-122), were evaluated for their ability to inhibit the formation of *p*-nitrophenol from *p*-nitrophenylphosphate (pNPP). The IC_50_ values obtained from these tests are detailed in Table 1. The newly synthesized compounds gave IC_50_ values ranging from 22.37 to 1.58 μM. The potency order for the indole derivatives of GA is as follows: **4b** > **4g** > **4a** > **4e** > **4c** > **4f** > **4d**. Several interesting observations about structure–activity relationships arose from these results. For example, comparing the inhibitory activity of the positive control FC-114 (IC_50_ = 1.75 μM), which has a trifluoromethyl group at the C5 position of the indole ring, with compounds **4a**, **4b**, and **4c**, which contain trifluoromethoxy, difluoromethoxy, and trifluoromethylthio groups, respectively, at the same position, reveals notable differences. Replacing the trifluoromethyl group with trifluoromethoxy (IC_50_ = 2.13 μM) slightly reduces biological activity. Substituting it with difluoromethoxy increases inhibitory activity, resulting in an IC_50_ of 1.58 μM. Conversely, replacing trifluoromethyl with trifluoromethylthio decreases inhibitory activity nearly three times, with an IC_50_ of 5.60 μM. We suggest that the oxygen atom in the trifluoromethoxy and difluoromethoxy groups is crucial for forming key interactions at the PTP1B binding site, likely acting as a hydrogen bond acceptor. This interaction does not occur with the sulfur atom found in the trifluoromethylthio group.

**Table 1:** Inhibitory Activity of PTP1B and TCPTP by GA Derivatives^a^

**compound**	*h* **PTP1B**_**1–400**_ **IC**_**50**_ **(μM ± S.D)**	*h* **PTP1B**_**1–285**_ **IC**_**50**_ **(μM ± S.D)**	*h* **TCPTP**_**1–415**_ **IC**_**50**_ **(μM ± S.D)**
**4a**	2.13 ± 0.13	ND	ND
**4b**	1.58 ± 0.07	7.03 ± 0.28	>100
**4c**	5.60 ± 0.12	ND	ND
**4d**	22.37 ± 0.53	ND	ND
**4e**	4.33 ± 0.58	ND	ND
**4f**	9.30 ± 0.25	ND	ND
**4g**	2.06 ± 0.12	9.11 ± 0.58	>100
**5a**	2.29 ± 0.15	ND	ND
**5b**	6.19 ± 0.58	ND	ND
**5c**	2.08 ± 0.04	ND	ND
**5d**	6.83 ± 0.17	ND	ND
**5f**	10.33 ± 0.51	ND	ND
**5g**	1.48 ± 0.18	5.51 ± 0.16	>100
FC-114	1.75 ± 0.08	ND	ND
FC-122	2.14 ± 0.15	ND	ND
GA	29.05 ± 0.03	ND	ND
Ursolic acid	5.61 ± 0.31	26.59 ± 1.46^b^	>400^b^
Ertiprotafib	2.16 ± 0.37	ND	>100
TCS401	8.1 ± 1.0^c^	12.4 ± 1.0^c^	6.7 ± 0.6^c^

a ND = Not determined.

b IC_50_ values retrieved from the reference^14^

c IC_50_ values retrieved from the reference^36^

Furthermore, when the trifluoromethyl group is replaced with a carboxy group in compound **4e**, the IC_50_ is 4.33 μM, indicating a nearly 2-fold decrease in potency. The substitution of the same group with carboxamide in compound **4f** leads to a dramatic drop in inhibitory activity to an IC_50_ of 9.30 μM, while replacing it with a cyano group in compound **4d** results in an even more considerable decrease in the inhibitory activity to an IC_50_ of 22.37 μM, representing reductions by factors of five and 12, respectively. Notably, incorporating a sulfonamide group in compound **4g** (IC_50_ = 2.06 μM) results in only a slight reduction in potency compared to the lead compound, FC-114.

These results highlight the importance of fluorinated derivatives in enhancing biological activity against PTP1B, a finding consistent with our previous study.^34^ Furthermore, our analysis of the **4d**–**g** derivatives indicates that having at least two hydrogen bond acceptor groups for the indole derivatives of GA is crucial, as seen for the derivative with the carboxy group (**4d**), which exists as a carboxylate at physiological pH, and the sulfonamide group derivative (**4g**) exhibited inhibitory activity on PTP1B comparable to that of the FC-114 compound.

Regarding the inhibitory activity of the *N*-phenylpyrazole derivatives of GA (**5a–g**), the potency order is as follows: **5g** > **5c** > **5a** > **5b** > **5d**. The **5e** derivative was found to be unstable and rapidly degraded during evaluation, so it was excluded from the study. The trend in inhibitory activity on PTP1B for these derivatives differs in some cases from what was observed with the indole derivatives of GA. For instance, among the fluorinated compounds, the **5c** (IC_50_ = 2.08 μM) derivative, which has the trifluoromethylthio group, is the most potent compared to the **5a** and **5b** derivatives, which are substituted with trifluoromethoxy and difluoromethoxy, respectively. Indeed, the **5c** derivative is slightly more potent than the lead compound FC-122 (IC_50_ = 2.14 μM), which contains a methyl group in the phenyl ring.

On the other hand, the **5d** and **5f** derivatives, which contain a cyano or carboxamide group, exhibit reduced inhibitory activity on PTP1B, similar to the findings with the indole derivatives of GA. As noted in our previous work,^34^ we believe that bulky and hydrophobic groups enhance the inhibitory activity against PTP1B within this series. However, an exception to this trend is compound **5g** (IC_50_ = 1.48 μM), which contains a sulfonamide group and is the most potent in this series, even surpassing the lead compound FC-122.

Among both series, the most potent compounds were **4a**, **4b**, **4e**, **4g**, **5c,** and **5g**. The lipophilicity values, calculated using SwissADME^40,41^ server (http://www.swissadme.ch/) and represented as Log *P*, for the indole-fused GA derivatives **4a**, **4b**, **4e**, and **4g** are 7.68, 7.38, 6.41, and 5.69, respectively. These values are lower than those of the lead compound FC-114 (Log *P* = 7.86). In contrast, the calculated lipophilicity values for the pyrazole-fused GA derivatives **5c** and **5g** are 7.97 and 5.39, respectively; only compound **5g** exhibits a lower lipophilicity than the lead compound FC-122 (Log *P* = 6.93). Given that this study aims to identify compounds with lower Log *P* values than their analogues FC-114 and FC-122, compound **5c** was excluded from further investigations.

Interestingly, the PTP1B inhibitory activity of **4a**, **4b**, **4e**, **4g**, and **5g** is more pronounced than that shown by ursolic acid (IC_50_ = 5.61 μM) and TCS401 (IC_50_ = 8.1 μM), which are known and widely reported inhibitors of PTP1B.^14,42–45^ Furthermore, the GA derivatives **4b** and **5g** demonstrate more pronounced inhibitory activity than that of Ertiprotafib (IC_50_ = 2.16 μM), a gold standard inhibitor of PTP1B that reached the phase II clinical trial for diabetes treatment,^46–49^ but unfortunately it failed due to its undesired side effects.^6^

Conversely, the most potent compounds **4b**, **4g**, and **5c** were assessed against the short form of PTP1B (*h*PTP1B_1–285_), which lacks the intrinsically disordered C-terminal region. The findings from this study indicate that these compounds are nearly 4-fold less potent against this form, with IC_50_ values of 7.03, 9.11, and 5.51 μM, respectively. This suggests that the residues within the disordered C-terminal region of PTP1B_1–400_ are critical for the binding of the GA derivatives tested. In contrast to analogous regions in other PTP family proteins, the intrinsically disordered C-terminal domain of PTP1B (residues 301–400) contains two α-helices, α8′ (residues 320–327) and α9′ (residues 360–377), which may act as binding sites for various compounds. Notably, due to the absence of homology in this domain with other Protein Tyrosine Phosphatases (PTPs), it has been suggested that the ability of compounds to bind to this site enhances the selectivity of PTP1B inhibitors compared to other PTP proteins, such as TCPTP.^50,51^ A prominent example of this selectivity is trodusquemine (MSI-1436), a highly selective noncompetitive PTP1B inhibitor that directly interacts within the disordered C-terminal region, demonstrating its preference for PTP1B.^52,53^ Therefore, based on this case, it is expected that the derivatives **4b** and **5g** will show selectivity for the PTP1B enzyme over TCPTP.

#### TCPTP Inhibitory Activity

The effectiveness and safety of a PTP1B inhibitor primarily depend on its selectivity, given that protein tyrosine phosphatases share significant structural homology within their catalytic domains. For instance, PTP1B and TCPTP exhibit a high degree of similarity, with 74% homology in their amino acid sequences.^38,39^ To assess the selectivity of GA derivatives **4b** and **5g**, these compounds were tested against *h*TCPTP_1–415_. Ertiprotafib was also included in this study. As illustrated in Table 1, the findings indicated that none of the synthesized molecules, along with Ertiprotafib, demonstrated inhibitory activity against TCPTP at concentrations of up to 100 μM, suggesting that they are at least 63 to 23-fold more selective for PTP1B over TCPTP. In contrast, the PTP1B inhibitor TCS401, previously tested by our group, displayed a lack of selectivity, inhibiting both the PTP1B and TCPTP enzymes with IC_50_ values of 8.1 and 6.7 μM, respectively.^36^

#### Enzymatic Kinetics Studies

Compounds **4a**, **4b**, **4e**, **4g**, and **5g** were selected for enzyme kinetic assays to determine their mode of inhibition against *h*PTP1B_1–400_. Kinetic analyses were conducted using varying concentrations of substrate and inhibitor. The kinetic parameters are summarized in Table 2, and Figure 2 provides a graphical representation in the form of a Lineweaver–Burk plot. Ursolic acid and Ertiprotafib exhibited mixed-type and uncompetitive inhibition, respectively, with *K*_i_ values of 4.3 and 0.15 μM. Compounds **4a**, **4b**, **4e**, **4g**, and **5g** showed *K*_i_ values of 0.74, 0.32, 4.05, 1.62, and 0.72 μM, respectively. Notably, these compounds (except **4e**) demonstrate a higher affinity for PTP1B compared to Ursolic acid but less than Ertiprotafib. Interestingly, all GA derivatives displayed uncompetitive inhibition, indicating that these compounds bind to an allosteric site of PTP1B_1–400_ only when the substrate is already bound to the catalytic site (E-S complex), thereby forming an enzyme–substrate-inhibitor complex (E–S-I complex). In our study, Ertiprotafib exhibited uncompetitive inhibition, which differs from the noncompetitive inhibition reported elsewhere.^46^ However, it is worth noting that Ertiprotafib was previously assessed using *h*PTP1B_1–393_ rather than *h*PTP1B_1–400_.

**Table 2:** Kinetic Parameters of *h*PTP1B_1‑400_ with Different Inhibitors

**compound**	***K**_**i**_* **(μM)**	***K***_**m**_ **(mM)**	*V*_**max**_ **(mM/min)**	**inhibition type**
**4a**	0.74 ± 0.10	1.84 ± 0.19	1.06 ± 0.24	uncompetitive
**4b**	0.32 ± 0.05	0.76 ± 0.17	1.24 ± 0.18	uncompetitive
**4e**	4.05 ± 0.67	0.99 ± 0.19	1.64 ± 0.23	uncompetitive
**4g**	1.62 ± 0.19	0.87 ± 0.13	1.46 ± 0.15	uncompetitive
**5g**	0.72 ± 0.12	0.97 ± 0.20	1.34 ± 0.21	uncompetitive
Ertiprotafib	0.15 ± 0.02	0.94 ± 0.19	1.71 ± 0.24	uncompetitive
ursolic acid	4.03 ± 0.80	1.70 ± 0.33	0.63 ± 0.08	mixed

**Figure 2:**
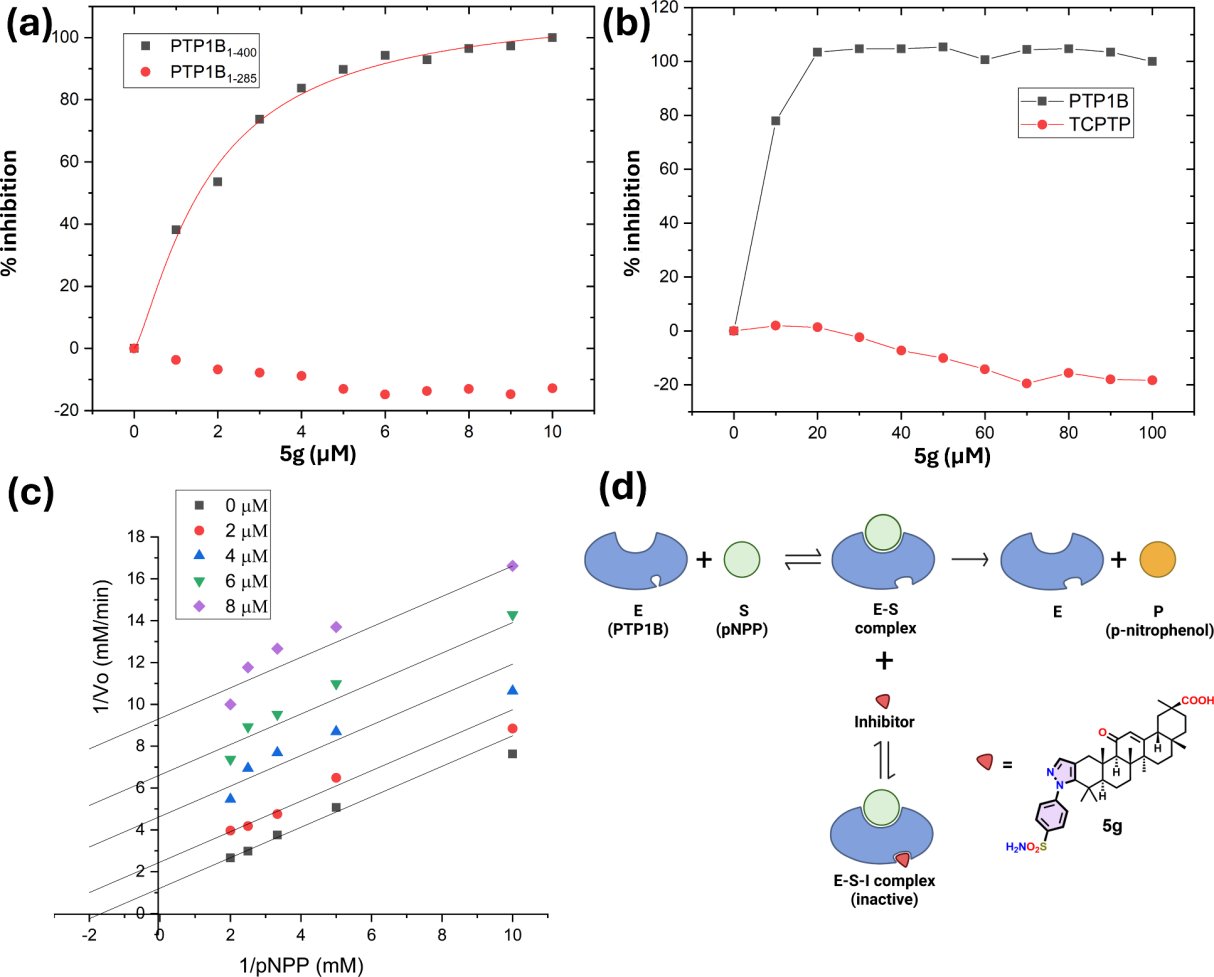
(**a**) Inhibitory activity of **5g** against long and short forms of PTP1B (*h*PTP1B_1–400_ and *h*PTP1B_1–285_). (**b**) Comparison of the Inhibitory activity of **5g** against *h*PTP1B_1–400_ and *h*TCPTP_1–415_. (**c**) Enzyme kinetics of *h*PTP1B_1–400_ inhibition. The Lineweaver–Burk plot shows the mechanism of inhibition of **5g**. The plot represents the reciprocal of the reaction velocity (1/V) as a function of the reciprocal pNPP concentration (1/pNPP). Data are representative of two independent experiments. The inhibitory mechanism for each compound was determined by fitting data to the equations defined for competitive, noncompetitive, uncompetitive, and mixed inhibition models. Data displayed in the graphs correspond to the best fit for each inhibition model (based on the *R* ^*2*^ coefficient) (OriginPro 2018 (64 bit) SR1). (**d**) Uncompetitive inhibition model. E: enzyme; S: substrate; I: inhibitor; P: product. Adapted from “Uncompetitive inhibition”, in BioRender. Matuz Mares, D. (2025) https://BioRender.com/do1oh1y.

The uncompetitive inhibition of PTP1B by GA derivatives offers a promising strategy for developing a new class of PTP1B inhibitors, as competitive or noncompetitive inhibitors typically target PTP1B. Considering the preferential inhibitory activity of **4a**, **4b**, **4e**, **4g**, and **5g** on the long form of PTP1B (*h*PTP1B_1–400_) compared to the short form (*h*PTP1B_1–285_), as well as their type of inhibition along with the lack of activity on TCPTP, we suggest that GA derivatives exert their inhibitory effects on PTP1B by interacting within the unstructured C-terminal site.

#### Molecular Docking

To explore the potential binding modes of GA derivatives (**4a**, **4b**, **4e**, **4g**, and **5g**) with the long form of PTP1B (PTP1B_1–400_), molecular docking simulations were conducted. Given that the PTP1B inhibition by GA derivatives is uncompetitive, we developed a three-dimensional model of the PTP1B_1–400_ enzyme complexed with the pNPP substrate at the catalytic site to form the enzyme–substrate complex (PTP1B_1–400_-pNPP). Due to the lack of a resolved structure for the PTP1B_1–400_ enzyme by X-ray diffraction, electron microscopy, or NMR, we obtained the structure of PTP1B_1–435_ from the AlphaFold^54–56^ database (code AF–P18031-F1). The last 35 residues were then removed to derive the PTP1B_1–400_ structure. This structure underwent Molecular Dynamics Simulations (MDS) using YASARA Structure^57–59^ software and the AMBER 11 ^60^ force field to achieve a biologically relevant folding of the unstructured C-terminal region. Afterward, the pNPP substrate was docked at the catalytic site of PTP1B_1–400_ using Vina, followed by submitting the protein–ligand complex to molecular dynamics simulations using the AMBER 11 force field, yielding the optimized structure of the PTP1B_1–400_-pNPP complex.

Docking simulations were conducted using the PTP1B_1–400_-pNPP structure along with compounds **4a**, **4b**, **4e**, **4g**, and **5g**. Three software programs were employed for this purpose: AutoDock (The Scripps Research Institute, La Jolla, CA, USA), Vina (also from The Scripps Research Institute), and GOLD (The Cambridge Crystallographic Data Centre, Cambridge, UK). These software programs were used to leverage various scoring functions, aiming to identify the most reliable solution for each protein–ligand interaction.

To determine the preferred binding sites for the synthesized compounds within the PTP1B_1–400_-pNPP complex, first, a blind docking simulation was performed using AutoDock and Vina. The findings from both programs revealed that the compounds exhibit a preference for three distinct zones in the PTP1B_1–400_-pNPP complex (Figure 3a), with a marked inclination toward the disordered C-terminal region rather than the catalytic domain. The first binding site (Site 1) is a pocket that comprises part of the p-Tyr Loop in the catalytic domain and a portion of the disordered C-terminal region, featuring the residues Lys^39^, Asn^42^, Arg^43^, Asp^61^, Cys^90^, His^92^, Phe^135^, Ala^384^, and Ala^385^. The second binding site (Site 2) is situated in the disordered C-terminal region, consisting of the residues Pro^319^, His^320^, Asn^321^, Lys^335^, Glu^336^, Glu^340^, Cys^344^, Ala^356^, and Pro^358^. The third binding site (Site 3), also located in the disordered C-terminal tail, includes the residues Lys^131^, Arg^380^, Ala^382^, Gln^383^, Pro^395^, and Pro^395^. Following this, site-directed docking simulations of the GA derivatives were performed using AutoDock, Vina, and GOLD. The docking scores and binding modes are presented in Table S1 and Figure S44 of the Supporting Information, respectively.

**Figure 3:**
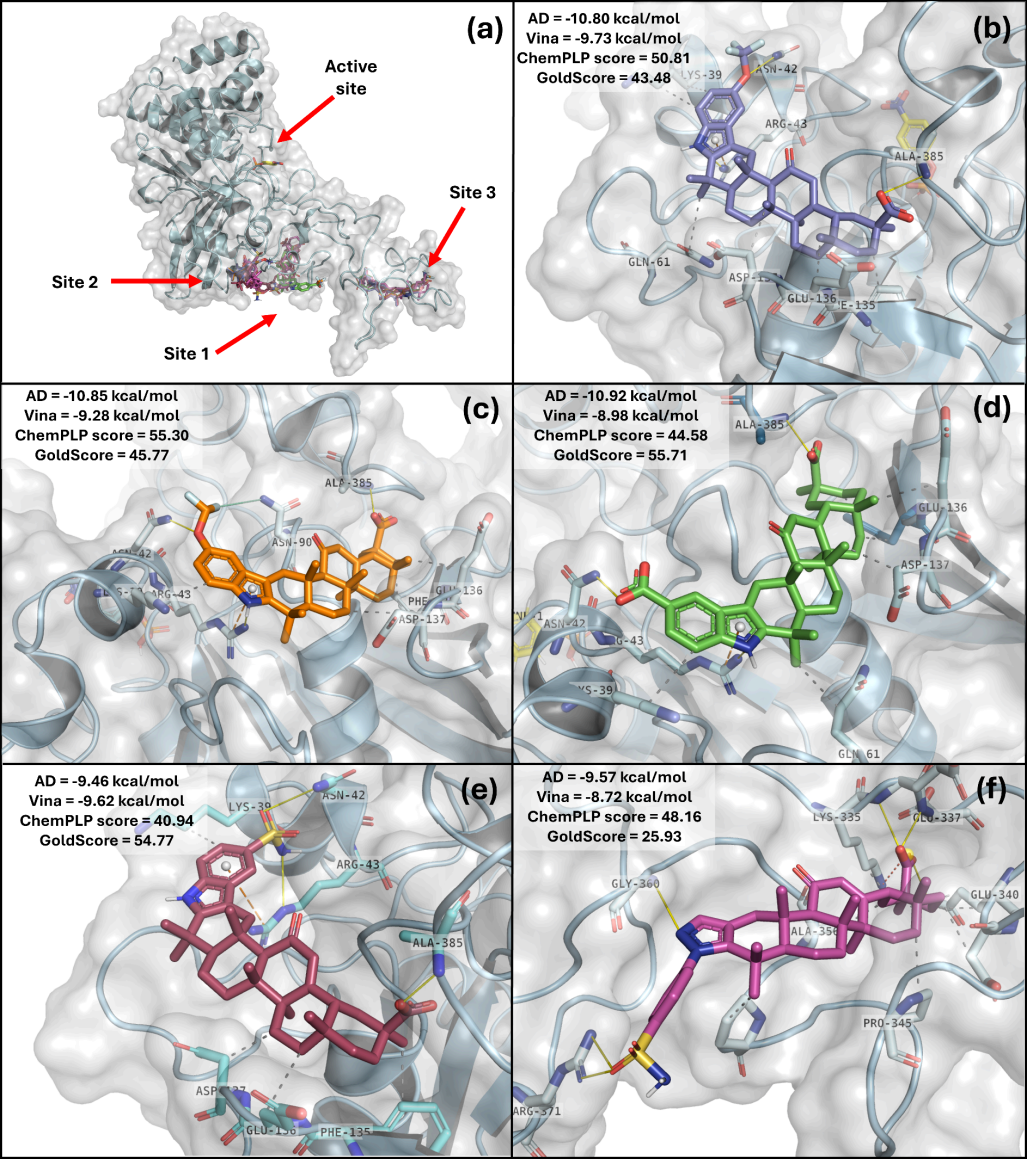
(a) Predicted binding modes of GA derivatives **4a** (blue), **4b** (orange), **4e** (green), **4g** (raspberry), and **5g** (magenta), after blind docking against PTP1B_1–400_-pNPP complex (pale cyan with gray surface). The structure of pNPP is represented in yellow. (b–e) Predicted binding mode of compounds **4a**, **4b**, **4e**, and **4g**, respectively, within site 2 of the PTP1B_1–400_-pNPP complex. (f) Predicted binding mode of **5g** within site 3 of the PTP1B_1–400_-pNPP complex. Solid yellow lines indicate hydrogen bond interactions, while red, orange, and gray dotted lines represent salt bridge interactions, π-cation interactions, and hydrophobic contacts, respectively.

The findings indicate that the fluorinated compounds **4a** and **4b** exhibit binding energies, as well as ChemPLP (CS) and GoldScore (GS) scores, that are more favorable for site 1. The binding energies of both compounds are quite similar, as indicated by the results from Autodock and Vina. However, the GOLD analysis reveals that compound **4b** achieves superior CS and GS values. Notably, this compound also exhibits high CS and GS scores at sites 2 and 3, indicating a slightly higher affinity than **4a** and suggesting the potential to bind to multiple sites on PTP1B. Compound **4e** has an improved binding energy according to Autodock and exhibits better CS and GS values, specifically displaying improved binding energy at site 2.

With respect to the derivatives **4g** and **5g**, although both compounds contain the same sulfonamide group, they exhibit distinct preferences for different binding sites. Compound **4g** exhibits more favorable binding energies at site 1, as indicated by the results from both Autodock and Vina. However, the GOLD analysis indicates that it has a comparable affinity for both sites 1 and 2. In contrast, compound **5g**, like **4g**, also demonstrates favorable binding energies at site 1; however, its CS and GS values are more favorable at site 3, suggesting that this molecule has a stronger affinity for that site.

Upon detailed observation of the binding poses of the GA derivatives within site 1, it is evident that the triterpene skeleton across all compounds establishes hydrophobic interactions with the residues Ala^382^, Ala^384^, and Pro^387^ (Supporting Information Figures S44a, S44d, S44g, S44j, and S45). Additionally, amide-π interactions with the indole ring and the Ser^386^ backbone are noted for compounds **4a**, **4b**, and **4c**. Notably, only derivatives **4a**, **4b**, and **4e** exhibit a similar binding pose, where the carbonyl group at position C11 acts as a hydrogen bond acceptor for the residues Glu^396^ and Lys^397^. Meanwhile, the fluorine atoms in compounds **4a** and **4b**, along with the carboxylate group in compound **4e**, participate in hydrogen bonding with Leu^394^. In contrast, the binding pose of compounds **4g** and **5g** differs; specifically, compound **4g** forms a salt bridge between the carboxylate group at position C30 and Arg^390^, while also forming a hydrogen bond between its carbonyl group at C11 and Gln^383^, which additionally engages in hydrogen bonding with one of the oxygen atoms of the sulfonamide group. As for compound **5g**, it displays π-anion-like interactions between the Glu^396^ residue and the benzenoid ring, along with interactions involving the sulfonamide group and the side chain of Ser^396^ (Supporting Information Figures S44m and S45e).

Regarding site 2 the indole derivatives **4a**, **4b**, **4e**, and **4g** (Figure 3b-3e) exhibit very similar binding poses (Supporting Information Figures S44b, S44e, S44h, S44k, and S46). In these compounds, the triterpene skeleton establishes hydrophobic interactions with the residues Cys^90^, His^92^, and Phe^135^, while the indole engages in a hydrophobic contact with Lys^39^.Furthermore, all four compounds form a hydrogen bond between the NH group of the indole and Asp^61^, with the indole ring also participating in a π-cation interaction with Arg.^43^ Notably, the trifluoromethoxy and difluoromethoxy groups in compounds **4a** and **4b**, respectively, act as hydrogen bond acceptors interacting with the Asn^42^ residue; compound **4b**, in particular, forms two interactions, compared to one for compound **4a**. In contrast, the sulfonamide group of compound **4g** functions as a hydrogen bond donor to Lys^39^. Interestingly, among these indole derivatives, only compounds **4b** and **4g** are positioned to form a hydrogen bond between the carboxylate group at position C30 and Ala^385^. Regarding compound **5g**, it displays an inverse orientation relative to the indole derivatives, establishing several significant interactions (Supporting Information Figures S44n and S46e). In this case, the carboxylate group at C30 forms both a hydrogen bond and a salt bridge with the residues Asn^62^ and Arg^43^, respectively, while the sulfonamide group acts as a hydrogen bond donor to Glu^136^.

Finally, at site 3, it was noted that indole derivatives **4a**, **4b**, **4e**, and **4g** exhibit a similar binding orientation (Supporting Information Figures S44c, S44f, S44i, S44l, and S47). In this case, the triterpene skeleton engages in hydrophobic interactions with residues Lys^335^, Pro^353^, Pro^358^, and Tyr^359^. The Ala^356^ residue participates in π-sigma interactions with the indole ring of all derivatives, with the exception of **4a**. Additionally, the four indole derivatives of GA form a salt bridge between the carboxylate group of C30 and the Arg^371^ residue. Conversely, the trifluoromethoxy and difluoromethoxy groups of compounds **4a** and **4b**, respectively, engage in halogen-like interactions with the Glu^340^ and Lys^350^ residues. These interactions are characterized by a dipole–dipole interaction between the fluorine atom and the carbonyl group of both residues, highlighting the importance of fluorinated derivatives at this binding site of PTP1B. Moreover, the Lys^350^ residue plays a crucial role in the interactions of compounds **4e** and **4g**, as in the first one, it forms a salt bridge with the carboxylate group of the indole ring, while in the latter, it establishes a hydrogen bond with the sulfonamide group attached to the indole ring. Notably, the **5g** derivative (Figure 3f) also displays an inverted binding orientation compared to the other indole derivatives, yet it forms the same hydrophobic interactions. In this structure, the sulfonamide group acts as a hydrogen bond acceptor with Arg^371^. At the same time, the carboxylate group in C30 engages in two significant interactions: a salt bridge with Lys^335^ and a hydrogen bridge with Glu^336^ (Supporting Information Figure S47e).

#### Molecular Dynamics Simulations (MDS)

To evaluate the stability of each complex formed between PTP1B_1–400_-pNPP and GA derivatives at the three distinct binding sites previously mentioned, we conducted molecular dynamics simulations. These simulations were performed using the YASARA Structure program and the AMBER 11 force field during a 300 ns time simulation. The root-mean-square deviation (RMSD) for the Cα (Figure 4a-4c) was determined, along with the binding energy calculated using the Mechanics Poisson–Boltzmann Surface Area (MM-PBSA) method for each ligand onto the PTP1B_1–400_-pNPP complex during the entire MDS (Figure 4d).

**Figure 4:**
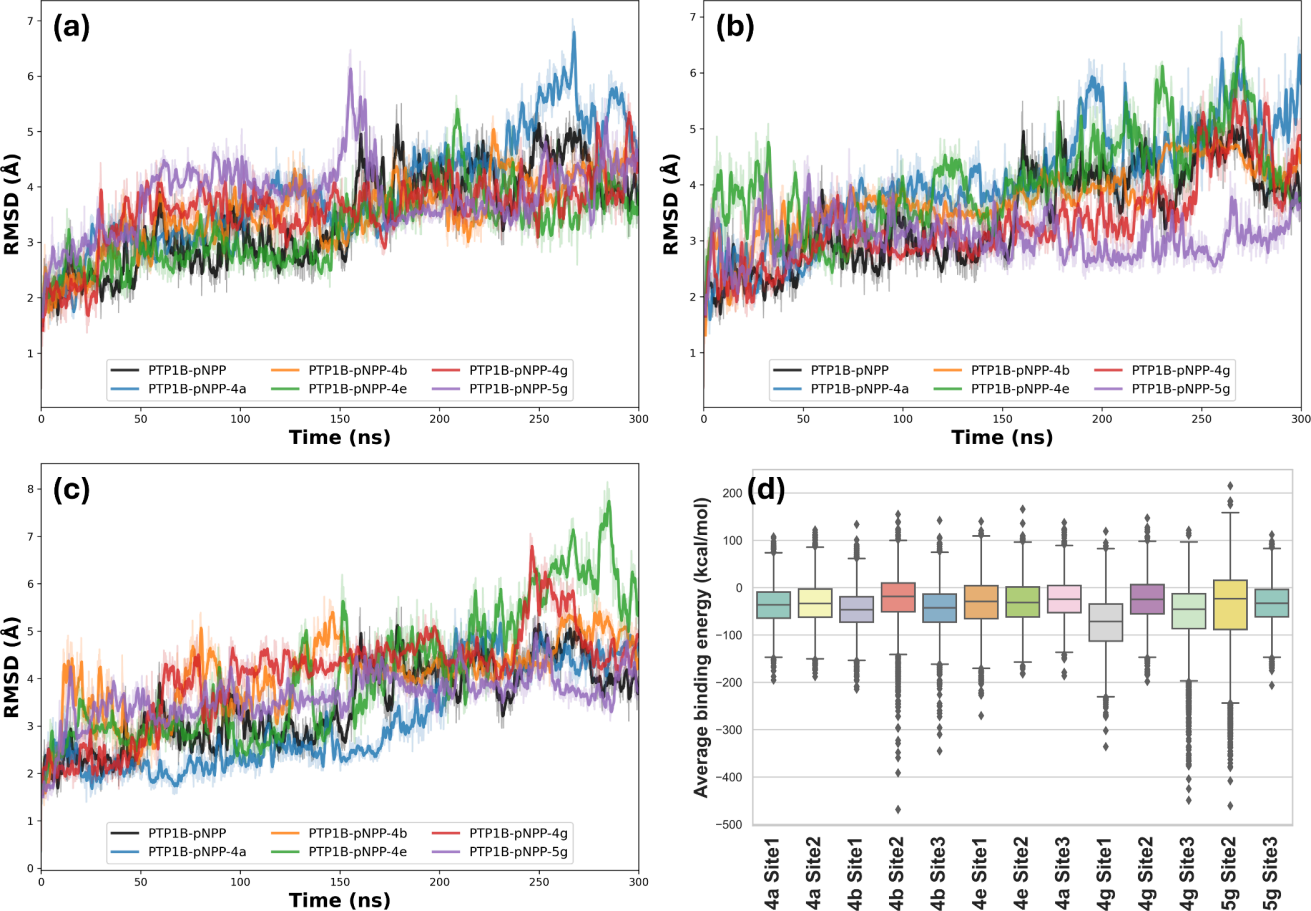
Standard RMSD of PTP1B_1–400_-pNPP complex, as well as PTP1B_1–400_-pNPP-ligand systems from docking complexes at site 1 (a), site 2 (b), and site 3 (c). Boxplot of average binding energy of ligands using the MM-PBSA method as a function of 300 ns of MD simulation within the PTP1B_1–400_-pNPP complex (d). The central line within the box represents the median of the binding energy. The lower and upper edges of the box correspond to the first (Q1) and third quartiles (Q3), respectively, defining the interquartile range (IQR). The whiskers extend to the minimum and maximum values that do not exceed 1.5 times the IQR. Points beyond the whiskers represent outliers.

The analysis of the overall fluctuation of the Cα carbons in the PTP1B_1–400_-pNPP system, as depicted in Figures 4a–4c, shows that stabilization occurs after 150 ns, with an average RMSD value of 3.45 Å. In examining the trajectory of the PTP1B_1–400_-pNPP-ligand complexes, it was observed that compounds **4a** and **5g** completely dissociated from the protein at sites 3 and 1, respectively. This suggests that the interactions of both compounds at these sites are weak.

Regarding the results of the RMSD plots at site 1, it can be observed that the PTP1B_1–400_-pNPP-**4e** complex has an average RMSD value of 3.25 Å, which is lower than the value observed for the PTP1B_1–400_-pNPP complex at 3.45 Å. The complexes PTP1B_1–400_-pNPP-**4b** (3.53 Å) and PTP1B_1–400_-pNPP-**4g** (3.57 Å) had RMSD values similar to that of PTP1B_1–400_-pNPP. This indicates that compound **4e**, followed by **4b** and **4g**, can primarily stabilize the overall conformation of the PTP1B_1–400_-pNPP complex when bound to site 1. At site 2, the complexes PTP1B_1–400_-pNPP-**4g** and PTP1B_1–400_-pNPP-**5g** exhibited lower RMSD values than the PTP1B_1–400_-pNPP complex, with values of 3.31 Å and 3.05 Å, respectively. This suggests that the presence of the sulfonamide group in compounds **4g** and **5g** enhances the stabilization of the PTP1B_1–400_-pNPP complex’s conformation. For site 3, all PTP1B_1–400_-pNPP-ligand complexes demonstrated higher RMSD values. However, it is noteworthy that the PTP1B_1–400_-pNPP-**5g** complex exhibited a lower RMSD value after 160 ns of simulation.

To quantitatively assess the affinity of the inhibitors at sites 1, 2, and 3 of the PTP1B_1–400_-pNPP complex, we performed a binding energy analysis over a 300 ns simulation using the MM-PBSA method (Figure 4d). The binding energy was derived by calculating the energy of the ligand-protein complex system (i.e., the bound state) and subtracting the energy at an infinite distance between the ligand and the protein system (i.e., the unbound state) every 100 ps. More negative values indicate better binding in the context of the AMBER11 force field. Calculations for compounds **4a** and **5g** at sites 1 and 3 were excluded since, as noted earlier, they dissociated from those binding sites.

The results indicated that GA derivatives have average binding energies ranging from −74.1 to −23.6 kcal/mol across the three binding sites. A more detailed analysis revealed that the fluorinated derivatives **4a** and **4b** exhibited better binding energy at site 1, with values of −36.4 and −46.3 kcal/mol, respectively. Notably, the presence of a difluoromethoxy group, as opposed to a trifluoromethoxy group, has a positive impact on binding with the PTP1B enzyme, aligning with the observed experimental inhibitory activity values. Compound **4e** demonstrated binding energies of −31.4, −30.0, and −24.1 kcal/mol for sites 1, 2, and 3, indicating that this molecule possesses similar affinity for the first two sites. However, this affinity is lower than that of all the other evaluated derivatives, which correlates with the observed experimental results, as this compound has the highest K_i_ value. On the other hand, compound **4g** shows a high affinity for sites 1 and 3, with binding energy values of −74.1 and −57.4 kcal/mol, respectively. Finally, it is noteworthy that compound **5g** exhibits a high affinity for site 2, with a binding energy value of −47.1 kcal/mol. Thus, it appears that the evaluated indole derivatives of GA form stable interactions with site 1, while the pyrazole derivative demonstrates stable interactions with site 2.

### *In vitro*, *In Vivo*, and Metabolomic Studies

#### Cytotoxic Activity against HFF-1 Cells

Given the significance of a drug candidate’s toxicity in relation to its inhibition potential, a cytotoxicity assay was conducted using Normal Human Foreskin Fibroblasts (HFF-1). These cells are a widely used noncancerous human fibroblast cell line in cytotoxicity assays for assessing the potential toxicity of biomaterials, nanoparticles, and other compounds on healthy cells.^61–66^ The concentration that resulted in a 50% reduction in HFF-1 proliferation (CC_50_) was evaluated for compounds **4b**, **4g**, and **5g**. The findings revealed a CC_50_ of 98.7 ± 2 μM for compound **4b**, while compounds **4g** and **5g** demonstrated a CC_50_ greater than 100 μM, indicating lower cytotoxicity compared to the results observed for GA and the lead compounds FC-114 and FC-122 (CC_50_ values of 62.0 ± 2, 59.5 ± 3, and 68.4 ± 1 μM, respectively).^35^ This suggests that substituting the trifluoromethyl group in FC-114 with a difluoromethoxy or sulfonamide group in the indole-fused GA derivatives has a positive impact on their cytotoxic profile. A similar trend is observed in the pyrazole-fused GA derivative, where a methyl group on the phenyl ring is replaced with a sulfonamide group.

#### Biological Assessment of GA Derivatives against Insulin-Resistant HepG2 Cells

To assess the potential effects of the most potent PTP1B inhibitors identified in this study on insulin and leptin signaling pathways, Western blotting was performed on HepG2 cells treated with compounds **4b**, **4g**, and **5g**. Metformin and Ursolic Acid were used as positive controls. HepG2 cells are a widely utilized human liver cell line known for maintaining hepatocyte insulin response mechanisms, making them an ideal model for studying insulin resistance in the liver. These models are typically established by exposing HepG2 cells to various inducers, such as high insulin. These treatments disrupt normal insulin signaling pathways, particularly the PI3K/Akt pathway, resulting in a state of cellular insulin resistance that resembles conditions in the human liver.^67–69^

Before conducting Western blotting, the viability of HepG2 cells treated with compounds was assessed at 24, 48, and 72 h. The findings of this study revealed that compounds **4b** and **5g** did not compromise HepG2 cell viability at 24, 48, and 72 h (Supporting Information Figure S48), demonstrating reduced cytotoxic activity. Thus, we evaluated the same compounds at 24 h in both a HepG2 normal model (Supporting Information Figure S49) and an insulin-resistant model (Supporting Information Figure S50). The results of this study indicated that neither metformin, ursolic acid, nor the GA derivatives significantly reduced PTP1B levels in either model. However, we observed a notable increase in AKT phosphorylation (p-AKT) levels with metformin in both models, while ursolic acid did not show significant changes in p-AKT, p-IRS, or p-STAT3 levels. Among the synthesized derivatives, only compound **4g** demonstrated a slight but not statistically significant increase in both p-IRS and p-STAT3 levels in the normal HepG2 cells, whereas compound **4b** showed only a minor increase in p-STAT3. In contrast, in the HepG2 insulin-resistant model, compound **5g** displayed a tendency to increase p-IRS levels.

#### Untargeted Metabolomics Analysis

HepG2 cells are commonly utilized in metabolomics to study alterations in metabolic pathways, such as glycolysis and lipid metabolism, when exposed to various substances,^70–75^ due to their ability to sustain liver-specific functions.^76^ To explore the metabolic changes induced by the **4b** and **5g** derivatives in HepG2 cells, an untargeted metabolomic analysis using GC-MS was carried out. Both compounds were selected for this study as they were the most potent PTP1B inhibitors. To visualize the primary changes in the metabolic profile, we utilized heatmap analysis (Figure 5a), incorporating all metabolites, which demonstrated that the untargeted approach effectively identified differences resulting from the treatments. Additionally, principal component analysis (PCA) (Figure 5b), an unsupervised multivariate method, revealed that the first and second components explained 63% and 25% of the total variance, respectively, indicating clear separation of all groups (R^2^: 0.66872; p-value based on 999 permutations: 0.002). Using the Kruskal–Wallis and Dunn’s tests, the findings showed that these triterpenes significantly altered the levels of seven metabolites, including glucose, fructose, pantothenic acid, palmitic acid, stearic acid, citric acid, and glycine. Analyzing these metabolites suggests that both compounds may positively influence glucose, fructose, and fatty acid metabolism (Figure 5c). Notably, compound **5g** significantly lowers intracellular levels of glucose and fructose while markedly increasing glycine levels, which is biosynthesized from 3-phosphoglycerate (3PGA)^77^ via serine/glycine synthesis pathway,^78^ therefore suggesting a potential enhancement in glycolysis. It is worth mentioning that the impaired *de novo* synthesis of glycine, as well as lower serum levels of this amino acid, are associated with obesity, insulin resistance, and T2DM.^79,80^ Additionally, **5g** leads to a significant increase in pantothenic acid (vitamin B5) levels. This vitamin is essential for synthesizing coenzyme A (CoA), which in turn produces acetyl-CoA and succinyl-CoA, all of which are crucial for ATP production in the tricarboxylic acid (TCA) cycle.^81^ Moreover, CoA increases mitochondrial activity due to its role in fat and carbohydrate metabolism, and to a lesser extent, via protein metabolism.^82,83^ Additionally, pantothenic acid has been shown to enhance energy expenditure, improve glucose tolerance, and reduce obesity in mice. On the other hand, our findings indicate that **5g** leads to a reduction in intracellular citrate levels, which may promote glycolysis and diminish gluconeogenesis similarly to metformin.^84^ Consequently, this could potentially lower hepatic glucose output. In summary, the increased intracellular levels of pantothenic acid and glycine, combined with the lowered citrate levels achieved by **5g**, may play a significant role in improving insulin resistance and T2DM.

**Figure 5:**
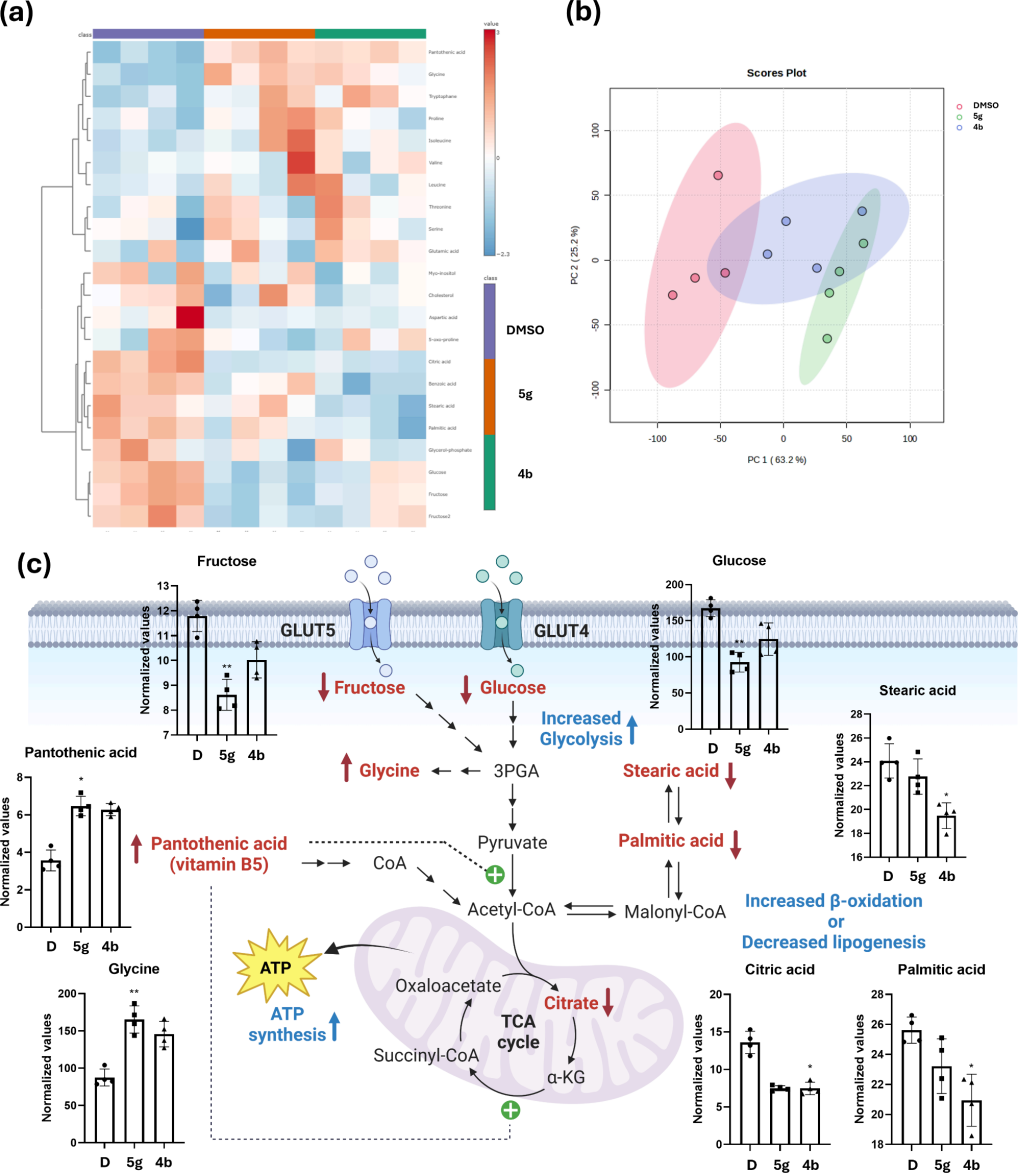
GC-MS-based untargeted metabolomic analysis of HepG2. (a) Heatmap analysis of HepG2 cells treated with DMSO (D, vehicle), **4b**, or **5g** for 24 h. (b) Principal Component Analysis (PCA) of untargeted metabolomic analysis of HepG2 treated with DMSO (vehicle), **4b**, and **5g** at 30 μM for 24 h (c). Statistically significant metabolites altered between the groups. Graphs were constructed using sum-normalized peak height values. Asterisks indicate statistical significance according to the Dunn test, with *p*-values denoted as follows: *p < 0.05, **p < 0.01. Created in BioRender. Matuz Mares, D. (2025) https://BioRender.com/hpj5plg.

In contrast, the metabolomic analysis of HepG2 cells treated with **4b** showed that this compound exhibits similar effects to those of **5g**, albeit to a lesser degree, as the reductions in glucose and fructose levels, as well as the increases in glycine and pantothenic acid, are not statistically significant. Nevertheless, **4b** was found to significantly reduce the intracellular levels of palmitic and stearic acids. This reduction may indicate a decrease in lipogenesis or an increase in fatty acid beta-oxidation.^85,86^ The underlying mechanism could involve elevated levels of acetyl-CoA, which may enhance energy production and contribute to anaplerosis in the TCA cycle. Conversely, the increased *de novo* synthesis of palmitic and stearic acids is associated with insulin resistance, as these lipids can lead to inflammation and increased fat accumulation, particularly in the liver.^87–91^ In a word, compound **4b**’s ability to lower both lipid levels may provide a valuable strategy for treating insulin resistance and T2DM.

#### Hypoglycemic and Insulin Sensitivity Assessment on CD-1 Mice

The studies were carried out on compounds **4b**, **4g**, and **5g** using both normoglycemic CD1 mice and a mouse model of noninsulin-dependent diabetes mellitus (NIDDM). An oral glucose tolerance test (OGTT) was conducted on the normoglycemic mice to evaluate the hypoglycemic activity of the compounds, while an insulin tolerance test (ITT) was performed on hyperglycemic CD1 mice to assess the compounds’ capacity to enhance insulin sensitivity in streptozotocin-induced diabetic mice. Pioglitazone and GA were included as positive controls. The hypoglycemic effects and insulin-sensitizing activity of the compounds and GA were measured using a single intragastric dose of 50 mg/kg, whereas Pioglitazone was administered at a dose of 45 mg/kg.

As depicted in Figure 6a, the GA derivatives **4b**, **4g**, and **5g** did not cause hypoglycemia in normoglycemic CD1 mice at a dose of 50 mg/kg. In the evaluation of reference drugs, pioglitazone exhibited the most significant reduction in the hyperglycemic peak.

**Figure 6:**
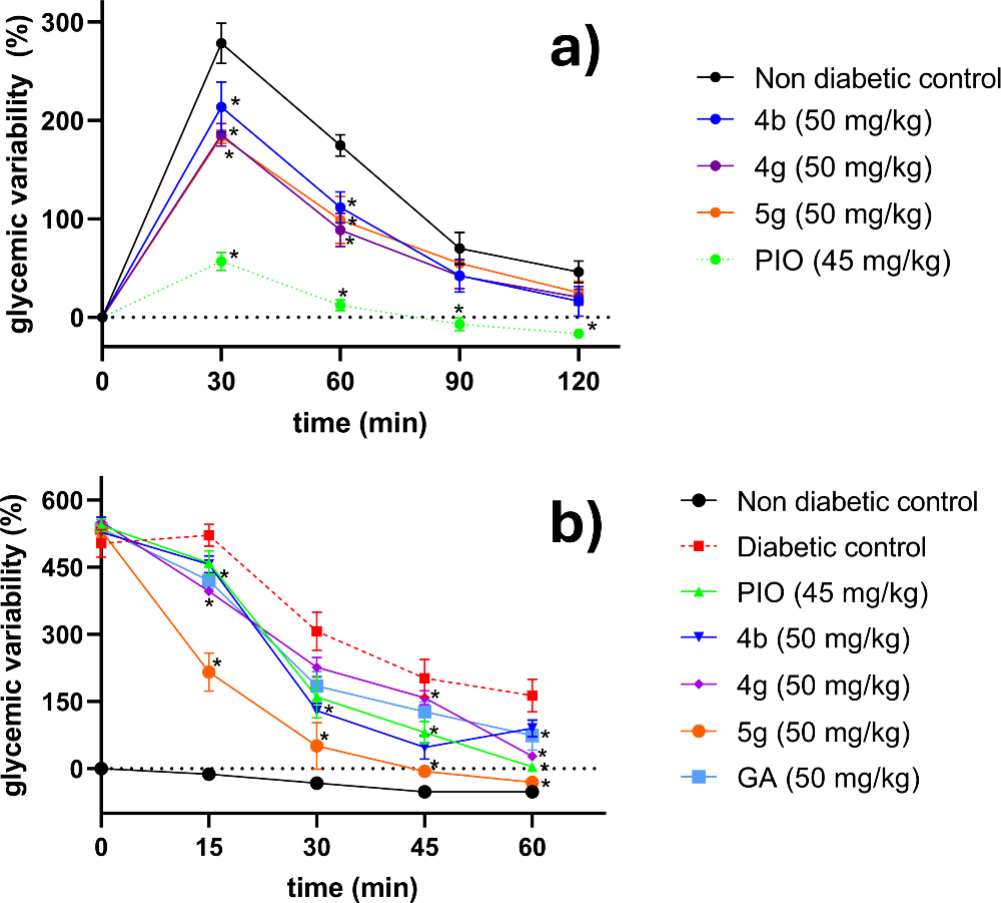
Percentage variation of glucose induced by compounds **4b**, **4g**, and **5g** in (a) the OGTT of normoglycemic CD1 mice and (b) the ITT of diabetic CD1 mice (*n* = 4). Pioglitazone (PIO) and GA were used as controls.

CD-1 mice with STZ-induced diabetes have been observed to develop insulin resistance.^92^ Therefore, compounds **4b**, **4g**, and **5g**, along with pioglitazone and GA, were assessed in diabetic animals for their capacity to enhance insulin sensitivity (Figure 6b). The results show that both pioglitazone and GA were found to markedly decrease glycemia variation compared to the saline solution group. Regarding the triterpenes **4b**, **4g**, and **5g**, all three demonstrated the ability to reduce glycemia variation starting from the 15th minute, suggesting their capability to improve insulin sensitivity. However, only compounds **4b** and **5g** displayed greater potency than GA. Notably, compound **5g** showed superior activity compared to pioglitazone, achieving significance from the 15th minute of the test. Additionally, statistical analysis indicated no significant differences in glycemic variation between the group of diabetic animals treated with compound **5g** and normoglycemic mice after the 45th minute of the ITT. This suggests that compound **5g** enhances insulin sensitivity to such an extent that blood glucose levels in diabetic mice resemble those of normoglycemic animals. These findings position compound **5g** as a promising new lead compound.

## Conclusions

Fourteen glycyrrhetinic acid derivatives were synthesized, achieving overall yields of up to 54% for indole-fused GA derivatives and 46% for pyrazole-fused GA derivatives. Among these, compounds **4a**, **4b**, **4e**, **4g**, and **5g**, which feature trifluoromethoxy, difluoromethoxy, carboxylic acid, and sulfonamide groups, demonstrated the most potent inhibition of the PTP1B enzyme in its long form (*h*PTP1B_1–400_). These compounds displayed selectivity by remaining inactive against the TCPTP enzyme. Furthermore, these semisynthetic triterpenes exhibited an uncompetitive inhibition type, with molecular docking studies and molecular dynamics simulations suggesting that they bind to the intrinsically unstructured C-terminal site of PTP1B.

Cytotoxicity studies conducted on HFF-1 cells showed that compounds **4b**, **4g**, and **5g** have a higher cytotoxic concentration 50 (CC_50_) compared to glycyrrhetinic acid, suggesting that they may have a safer profile. Furthermore, a nontargeted metabolomics analysis indicated triterpenes **4b** and **5g** increased intracellular levels of pantothenic acid and glycine, while decreasing glucose, fructose, citric acid, palmitic acid, and stearic acid, suggesting an enhancement of glycolysis alongside a reduction in lipogenesis or an increase in β-oxidation in HepG2 cells. Lastly, it was found that these compounds improve insulin sensitivity in STZ-induced diabetic CD1 mice, with compound **5g** exhibiting the most significant effect, even outperforming Pioglitazone.

## Experimental Section

### General Experimental Procedures

All reagents and starting materials were acquired from Sigma-Aldrich (Toluca, MEX, Mexico, and St. Louis, MO, USA). Ertiprotafib was acquired from ChemMedExpress (Monmouth Junction, NJ, USA) while Pioglitazone and Glycyrrhetinic Acid were acquired from Biosynth (Compton, UK). Reactions were monitored using thin-layer chromatography (TLC) on 0.2 mm silica gel-coated 60 F254 plates (Sigma-Aldrich) and visualized under UV light. Melting points were measured with a Fischer-Johns melting point apparatus without correction. Both ^1^H and ^13^C NMR spectra were recorded on Agilent DD2 (Agilent, Santa Clara, CA, USA) and Bruker Ascend spectrometers (Bruker, Billerica, MA, USA), operating at 600 MHz for ^1^H, and at 151 MHz for ^13^C, respectively. Chemical shifts are reported in parts per million (ppm) relative to tetramethylsilane (Me_4_Si = 0); coupling constants (J values) are expressed in Hertz (Hz). Multiple patterns are indicated as follows: s, singlet; d, doublet; q, quartet; dd, doublet of doublets; t, triplet; m, multiplet; br s, broad singlet. In addition, the purity of the compounds was determined using the Agilent DD2 spectrometer by quantitative nuclear magnetic resonance (^1^H-qNMR) spectroscopy in DMSO-*d*_6_ at 298 K, with sodium 4,4-dimethyl-4-silapentane-1-sulfonate (DSS) as the internal standard. The purity was calculated according to the ″Purity by Absolute qNMR Instructions″ (Table S2).^93^

High-resolution mass spectra (HRMS) were obtained using a micrOTOF-ESI-TOF-MS mass spectrometer via direct infusion in positive mode, employing nitrogen (4 mL/min) as the nebulizer gas, with a spray voltage of 4.5 kV at 150 °C, across a mass range of *m*/*z* 50–3000. The results are expressed as *m*/*z*. All data are included in the Supporting Information. Following IUPAC guidelines, compounds were named using the automatic generator tool integrated into ChemDraw Professional 22.0.0 software (PerkinElmer, Waltham, MA, USA). The synthesis of compounds **4a** and **5g** was previously reported by Amit Kumar et al. (2022)^94^ and Yuanyuan Wang et al. (2021),^95^ respectively. However, both compounds were prepared here with some modifications.

For the insulin-resistant model using HepG2 cells, the following reagents were acquired from Sigma-Aldrich (Toluca, MEX, Mexico, and St. Louis, MO, USA): Insulin (I2643), Dulbecco’s Modified Eagle’s Medium (DMEM) low-glucose media (D5523), Trypsin-EDTA solution (T4174), antibiotic-antimycotic solution­(A5955), dimethyl sulfoxide (DMSO–D8418), sodium deoxycholate (D6750), triethylammonium bicarbonate buffer 1 M (TEAB-T7408), glutaraldehyde 25% (G6275), acetic acid (695092), and crystal violet (C0775). The bicinchoninic acid protein quantitation kit (23225) and the Halt Cocktail with protease and phosphatase inhibitors (1861281) were acquired from Thermo Fisher Scientific. Fetal bovine serum (26140–079) was sourced from Gibco. The chemiluminescent solution (170–5061) was obtained from Biorad. Rabbit anti-PTP1B (5311S), IRS (2382S), p-IRS (2381T), Akt (9272), p-Akt (4060S), STAT3 (12640S), p-STAT3 (9145S), and mouse anti-β actin (3700S) primary antibodies were obtained from Cell Signaling, while secondary antibodies conjugated to horseradish peroxidase were from Jackson Laboratories (rabbit-111–035, mouse-115–035).

### General Procedure for Fischer Indolization (**4a**–**4g**)

These compounds were prepared from compound **2**, following the method described by De-la-Cruz-Martínez et al. (2021).^34^

#### (20β)-5′-(Trifluoromethoxy)-11-oxo-1′*H*-oleana-2,12-dieno­[3,2-*b*]­indol-29-oic Acid (**4a**)

Yellow solid. Yield 37%. Mp. 237–240 °C. ^1^H NMR (600 MHz, DMSO-*d*_6_) δ_H_ 12.04 (bs, 1H), 11.02 (bs, 1H), 7.30 (d, *J* = 8.65 Hz, 1H), 7.16 (s, 1H), 6.95 (dd, *J* = 1.44, 8.67, 1H), 5.51 (s, 1H), 3.67 (d, *J* = 15.35 Hz, 1H), 2.68 (s, 1H), 2.25 (d, *J* = 15.31 Hz, 1H), 1.40 (s, 3H), 1.30 (s, 3H), 1.22 (s, 3H), 1.13 (s, 3H), 1.12 (s, 3H), 1.06 (s, 3H), 0.80 (s, 3H). ^13^C NMR (151 MHz, DMSO-*d*_6_) δ_C_ 198.76, 177.66, 170.06, 143.66, 141.18, 134.73, 132.54, 127.57, 120.51 (q, *J =* 253.74 Hz), 113.41, 111.29, 109.37, 105.69, 59.64, 52.22, 48.06, 44.85, 43.09, 40.77, 37.49, 37.55, 36.82, 33.99, 31.57, 31.34, 30.44, 30.39, 28.46, 27.82, 26.16, 25.84, 22.91, 21.05, 18.03, 17.95, 15.79. HRMS (ESI-MS) *m*/*z* for C_37_H_46_F_3_NO_4_^+^ [M + H]^+^ calc. 626.3457; found 626.3451. Purity determined by ^1^H-qNMR was 95.9 ± 0.2% (n = 3).

#### (20β)-5′-(Difluoromethoxy)-11-oxo-1′*H*-oleana-2,12-dieno­[3,2-*b*]­indol-29-oic Acid (**4b**)

Light green solid. Yield 49%. Mp. 235–238 °C. ^1^H NMR (600 MHz, DMSO-*d*_6_) δ_H_ 7.77 (bs, 1H), 7.23 (m, 2H), 6.92 (dd, *J* = 8.59 and 2.20 Hz), 6.85 (t, *J* = 75.28 Hz, 1H), 5.80 (s, 1H), 3.89 (d, *J* = 15.51 Hz, 1H), 2.66 (s, 1H), 2.25 (m, 1H), 1.42 (s, 3H), 1.32 (s, 3H), 1.26 (s, 3H), 1.24 (s, 3H), 1.22 (s, 3H), 1.18 (s, 3H), 0.88 (s, 3H). ^13^C NMR (151 MHz, DMSO-*d*_6_) δ_C_ 198.80, 177.67, 170.04, 143.69, 143.10, 133.86, 127.71, 117.32 (t, *J* = 256.19 Hz), 112.69, 111.22, 107.40, 105.40, 59.69, 52.31, 48.07, 44.87, 43.10, 40.79, 37.54, 36.96, 33.99, 31.58, 31.37, 30.51, 30.41, 28.47, 27.85, 26.17, 25.86, 22.95, 21.06, 18.06, 17.97, 16.24. HRMS (ESI-MS) *m*/*z* for C_37_H_47_F_2_NO_4_^+^ [M + H]^+^ calc. 608.3551; found 608.3550. Purity determined by ^1^H-qNMR was 97.9 ± 0.5% (n = 3).

#### (20β)-5′-(Trifluormethylthio)-11-oxo-1′*H*-oleana-2,12-dieno­[3,2-*b*]­indol-29-oic Acid (**4c**)

Light green solid. Yield 47%. Mp. 231–233 °C. ^1^H NMR (600 MHz, CDCl_3_) δ_H_ 12.21 (bs, 1H), 11.22 (bs, 1H), 7.59 (s, 1H), 7.38 (d, *J* = 8.33 Hz, 1H), 7.28 (d, *J* = 8.22 Hz, 1H), 5.52 (s, 1H), 3.72 (d, *J* = 15.41 Hz), 2.69 (s, 1H), 2.29 (d, *J* = 15.57 Hz, 1H), 1.41 (s, 3H), 1.30 (s, 3H), 1.22 (s, 3H), 1.13 (s, 3H), 1.05 (s, 3H), 1.01 (s, 3H), 0.80 (s, 3H). ^13^C NMR (151 MHz, DMSO-*d*_6_) δ_C_ 198.72, 177.64, 170.05, 143.29, 137.60, 130.01 (*J* = 307.77 Hz), 128.57, 127.78, 127.48, 126.47, 111.88, 110.03, 105.74, 59.65, 52.24, 48.06, 44.84, 43.09, 40.80, 37.55, 37.47, 36.74, 33.95, 31.56, 31.34, 30.68, 30.41, 31.34, 29.28, 28.45, 27.83, 26.16, 25.85, 22.92, 22.82, 18.01, 17.95, 15.80. HRMS (ESI-MS) *m*/*z* for C_37_H_46_F_3_NO_3_S^+^ [M + H]^+^ calc. 642.3229; found 642.3226. Purity determined by ^1^H-qNMR was 98.0 ± 0.4% (n = 3).

#### (20β)-5′-Cyano-11-oxo-1′*H*-oleana-2,12-dieno­[3,2-*b*]­indol-29-oic Acid (**4d**)

White solid. Yield 27%. Mp. 263–264 °C. ^1^H NMR (600 MHz, DMSO-*d*_6_) δ_H_ 10.35 (bs, 1H), 7.77 (s, 1H), 7.43 (s, 1H), 7.35 (d, *J* = 8.32 Hz, 1H), 7.28 (d, J = 8.33 and 1.48 Hz, 1H), 5.77 (s, 1H), 3.90 (d, *J* = 15.51 Hz, 1H), 2.66 (s, 1H), 2.24 (d, *J* = 15.64 Hz, 1H), 1.43 (s, 3H), 1.35 (s, 3H), 1.28 (s, 3H), 1.21 (s, 3H), 1.19 (s, 3H), 1.14 (s, 3H), 0.85 (s, 3H). ^13^C NMR (151 MHz, DMSO-*d*_6_) δ_C_ 198.71, 177.68, 170.12, 144.88, 138.16, 127.41, 123.13, 122.59, 121.07, 111.66, 106.26, 99.82, 59.55, 52.11, 48.08, 44.86, 43.11, 40.76, 37.56, 37.47, 36.59, 31.58, 31.30, 30.39, 30.33, 28.47, 27.83, 26.18, 25.84, 22.95, 22.79, 21.08, 18.01, 17.96, 15.77. HRMS (ESI-MS) *m*/*z* for C_37_H_46_N_2_O_3_ [M + H]+ calc. 567.35881; found 567.3587. Purity determined by ^1^H-qNMR was 95.9 ± 0.8% (n = 3)

#### (20β)-5′-Carboxy-11-oxo-1′*H*-oleana-2,12-dieno­[3,2-*b*]­indol-29-oic Acid (**4e**)

White solid. Yield 31%. Mp. 269–274 °C. ^1^H NMR (600 MHz, DMSO-*d*_6_) δ 12.23 (bs, 1H), 11.10 (bs, 1H), 7.95 (s, 1H), 7.64 (dd, *J* = 8.67 and 1.44 Hz, 1H), 7.30 (d, *J* = 8.45 Hz, 1H), 5.52 (s, 1H), 3.74 (d, *J* = 15.59 Hz), 2.69 (s, 1H), 2.27 (d, *J* = 15.59 Hz, 1H), 1.41 (s, 3H), 1.31 (s, 3H), 1.22 (s, 3H), 1.14 (s, 3H), 1.12 (s, 3H), 1.05 (s, 3H), 0.80 (s, 3H). ^13^C NMR (151 MHz, DMSO-*d*_6_) δ_C_ 198.81, 177.66, 170.07, 168.52, 142.64, 138.89, 127.47, 127.09, 121.60, 120.63, 119.82, 110.16, 106.34, 59.65, 52.27, 48.09, 44.82, 43.09, 40.79, 40.48, 37.54, 37.49, 36.79, 33.94, 31.58, 31.33, 30.36, 28.47, 27.82, 25.84, 22.93, 22.82, 18.03, 17.95, 15.82. HRMS (ESI-MS) *m*/*z* for C_37_H_47_NO_5_^+^ [M + H]^+^ calc. 586.3532; found 586.3527. Purity determined by ^1^H-qNMR was 96.8 ± 0.5% (n = 3).

#### (20β)-5′-Carbamoyl-11-oxo-1′*H*-oleana-2,12-dieno­[3,2-*b*]­indol-29-oic Acid (**4f**)

Ivory solid. Yield 67%. Mp. > 300 °C. ^1^H NMR (600 MHz, DMSO-*d*_6_) δ 12.21 (bs, 1H), 10.93 (bs, 1H), 7.93 (s, 1H), 7.85 (bs, 1H), 7.58 (dd, *J* = 8.44 and 1.54 Hz, 1H), 7.24 (d, *J* = 8.43 Hz, 1H), 6.94 (bs, 1H), 5.52 (s, 1H), 3.79 (d, *J* = 15.40 Hz, 1H), 2.69 (s, 1H), 2.25 (d, *J* = 15.55 Hz, 1H), 1.42 (s, 3H), 1.31 (s, 3H), 1.22 (s, 3H), 1.14 (s, 3H), 1.12 (s, 3H), 1.06 (s, 3H), 0.80 (s, 3H). ^13^C NMR (151 MHz, DMSO-*d*_6_) δ 198.82, 177.72, 170.10, 169.30, 142.20, 138.01, 127.49, 126.98, 124.11, 120.22, 117.74, 109.80, 106.16, 59.73, 52.35, 48.08, 44.92, 43.13, 40.82, 37.84, 37.01. 33.95, 31.59, 31.39, 30.46, 28.49, 27.86, 26.19, 25.86, 23.04, 22.92, 18.08, 17.98, 15.83. HRMS (ESI-MS) *m*/*z* for C_37_H_48_N_2_O_4_^+^ [M + H]^+^ calc. 585.3692; found 585.3688. Purity determined by ^1^H-qNMR was 93.5 ± 0.4% (n = 3)

#### (20β)-5′-Sulfamoyl-11-oxo-1′*H*-oleana-2,12-dieno­[3,2-*b*]­indol-29-oic Acid (**4g**)

Orange solid. Yield 26%. Mp. > 300 °C. ^1^H NMR (600 MHz, DMSO-*d*_6_) δ_H_ 12.20 (bs, 1H), 11.17 (bs, 1H), 7.82 (s, 1H), 7.47 (dd, *J* = 8.47 and 1.57 Hz, 1H), 7.36 (d, *J* = 8.46 Hz, 1H), 7.04 (bs, 2H), 5.53 (s, 2H), 3.78 (d, *J* = 15.36 Hz, 1H), 2.70 (s, 1H), 2.28 (d, *J* = 15.50 Hz, 1H), 1.42 (s, 3H), 1.32 (s, 3H), 1.22 (s, 3H), 1.14 (s, 3H), 1.12 (s, 3H), 1.04 (s, 3H), 0.80 (s, 3H). ^13^C NMR (151 MHz, DMSO-*d*_6_) δ_C_ 198.78, 177.64, 170.10, 143.43, 137.56, 133.95, 127.47, 127.09, 117.64, 115.87, 110.84, 110.34, 106.35, 59.61, 52.27, 48.08, 44.82, 43.09, 40.81, 37.52, 36.77, 34.01, 31.56, 31.32, 30.37, 28.45, 26.17 25.84, 22.97, 22.84, 18.03, 17.95, 15.75. HRMS (ESI-MS) *m*/*z* for C_36_H_48_N_2_O_5_S^+^ [M + H]^+^ calc. 621.3362; found 621.3359. Purity determined by ^1^H-qNMR was 95.3 ± 0.6% (n = 3).

### General Procedure for the Synthesis of *N*-Phenylpyrazole Ga Derivatives (**5a**–**5g**)

Compounds **5a**-**5g** were synthesized from compound **3** using the method described by De-la-Cruz-Martínez et al. (2021)^34^ (Method A). The reaction time was monitored by TLC over a period from 2 to 12 h. A second methodology (Method B) was also employed for compounds **5a**, **5c**, and **5d**–**5g**, which is described as follows: a solution of compound **3** (0.04 mmol) and the substituted phenylhydrazine hydrochloride (0.07 mmol) in DMF (4 mL) was allowed to stir at room temperature for 4 to 6 h, during which the reaction was monitored by TLC. The reaction mixture was then carefully poured into cold water, leading to the formation of a precipitate, which was subsequently filtered under vacuum. The resulting solid was dissolved in a 50:50 mixture of CH_2_Cl_2_ and ethyl acetate. The mixture was heated, and activated charcoal was added. After filtration through Celite, the filtrate was washed with the same solvent mixture. The recovered mother liquor was stirred and heated to a final volume of 15 mL, at which point hexanes were added dropwise until a precipitate formed. After cooling, the solid was finally filtered under vacuum to afford the final compounds.

#### (20β)-11-Oxo-2′-[4-(trifluoromethoxy)­phenyl]-2′*H*-oleana-2,12-dieno­[3,2-*c*]­pyrazol-29-oic acid (**5a**)

White solid. Yield 15% (Method A), 31% (Method B). Mp. 275–280 °C. ^1^H NMR (600 MHz, DMSO-*d*_6_) δ_H_ 12.21 (bs, 1H), 7.54 (d, *J* = 8.8 Hz, 2H), 7.49 (d, *J* = 8.5 Hz, 2H), 7.35 (s, 1H), 5.50 (s, 1H), 3.57 (d, *J* = 15.4 Hz, 1H), 2.59 (s, 1H), 2.18 (d, *J* = 15.5 Hz, 1H), 2.15 – 2.05 (m, 2H), 1.99 (s, 1H), 1.83 – 1.75 (m, 2H), 1.74 – 1.64 (m, 3H), 1.54 (d, *J* = 10.6 Hz, 1H), 1.42 (d, *J* = 11.9 Hz, 2H), 1.38 (s, 3H), 1.33 – 1.22 (m, 3H), 1.18 (dd, *J* = 17.2, 10.1 Hz, 2H), 1.11 (s, 3H), 1.10 (s, 3H), 1.06 (s, 3H), 1.00 (s, 3H), 0.97 (s, 3H), 0.78 (s, 3H). ^13^C NMR (151 MHz, DMSO-*d*_6_) δ 198.58, 177.67, 170.16, 148.26, 145.39, 141.16, 138.15, 131.11, 127.41, 120.01 (q, *J =* 256.86 Hz), 121.12, 113.88, 59.53, 53.37, 48.08, 44.65, 43.08, 40.77, 37.51, 36.82, 34.19, 31.58, 31.26, 30.35, 29.22, 28.46, 27.80, 26.17, 25.82, 22.85, 22.47, 17.85, 15.40. HRMS (ESI-MS) *m*/*z* for C_38_H_48_F_3_N_2_O_4_^+^ [M + H]^+^ calc. 653.3561; found 653.3564. Purity determined by ^1^H-qNMR was 94.6 ± 0.1% (n = 3).

#### (20β)-2′-[4-(Difluoromethoxy)­phenyl]-11-Oxo-2′*H*-oleana-2,12-dieno­[3,2-*c*]­pyrazol-29-oic Acid (**5b**)

White solid. Yield 30%. Mp. 240–246 °C. ^1^H NMR (600 MHz, DMSO-*d*_6_) δ_H_ 12.21 (bs, 1H), 7.44 (d, *J* = 8.8 Hz, 2H), 7.36 (s, 1H), 7.32 (s, 1H), 7.28 (d, *J* = 8.7 Hz, 2H), 5.50 (s, 1H), 3.57 (d, *J* = 15.4 Hz, 1H), 2.59 (s, 1H), 2.18 (d, *J* = 15.4 Hz, 1H), 2.10 (td, *J* = 12.6, 4.1 Hz, 2H), 1.99 (s, 1H), 1.83 – 1.75 (m, 2H), 1.75 – 1.66 (m, 3H), 1.54 (d, *J* = 10.7 Hz, 1H), 1.42 (d, *J* = 11.8 Hz, 2H), 1.38 (s, 3H), 1.32 – 1.23 (m, 3H), 1.18 (dd, *J* = 17.2, 10.0 Hz, 2H), 1.11 (s, 3H), 1.10 (s, 3H), 1.06 (s, 3H), 1.01 (s, 3H), 0.97 (s, 3H), 0.78 (s, 3H). ^13^C NMR (151 MHz, DMSO-*d*_6_) δ_C_ 198.57, 177.66, 170.13, 150.94, 145.24, 138.99, 137.86, 130.78, 127.42, 118.41, 118.25, 116.20, 116.20 (q, *J =* 258.20 Hz), 114.14, 113.67, 59.55, 53.42, 48.07, 44.65, 43.07, 40.78, 37.50, 36.87, 34.21, 31.58, 31.27, 30.36, 29.19, 28.46, 27.81, 26.17, 25.82, 22.85, 22.41, 17.86, 15.40. HRMS (ESI-MS) *m*/*z* for C_38_H_49_F_2_N_2_O_4_^+^ [M + H]^+^ calc. 635.3655; found 635.3668. Purity determined by ^1^H-qNMR was 92.9 ± 0.6% (n = 3).

#### (20β)-11-Oxo-2′-[4-(trifluoromethylthio)­phenyl]-2′H-oleana-2,12-dieno­[3,2-*c*]­pyrazol-29-oic Acid (**5c**)

Light yellow solid. Yield 15% (Method A). Mp. > 300 °C. ^1^H NMR (600 MHz, DMSO-*d*_6_) δ_H_ 12.19 (bs, 1H), 7.85 (d, *J* = 8.1 Hz, 2H), 7.57 (d, *J* = 8.2 Hz, 2H), 7.38 (s, 1H), 5.50 (s, 1H), 3.58 (d, *J* = 15.4 Hz, 1H), 2.60 (s, 1H), 2.19 (d, *J* = 15.4 Hz, 1H), 2.10 (dd, *J* = 12.4, 9.1 Hz, 2H), 1.85 – 1.74 (m, 2H), 1.75 – 1.65 (m, 3H), 1.54 (d, *J* = 11.1 Hz, 1H), 1.42 (d, *J* = 12.0 Hz, 2H), 1.38 (s, 3H), 1.35 – 1.15 (m, 5H), 1.11 (s, 3H), 1.10 (s, 3H), 1.06 (s, 3H), 1.00 (s, 3H), 0.97 (s, 3H), 0.78 (s, 3H). ^13^C NMR (151 MHz, DMSO-*d*_6_) δ_C_ 198.55, 177.65 170.15, 145.47, 144.83, 138.43, 136.40, 130.47, 129.52 (q, *J =* 307.95 Hz), 127.41, 124.00, 114.10, 59.52, 53.36, 48.07, 44.64, 43.07, 40.77, 37.49, 36.80, 34.21, 31.57, 31.25, 30.35, 29.26, 28.46, 27.80, 26.16, 25.81, 22.84, 22.52, 17.84, 15.40. HRMS (ESI-MS) *m*/*z* for C_38_H_48_F_3_N_2_O_3_S^+^ [M + H]^+^ calc. 669.3332; found 669.3331. Purity determined by ^1^H-qNMR was 97.6 ± 0.4% (n = 3).

#### (20β)-2′-(4-Cyanophenyl)-11-oxo-2′*H*-oleana-2,12-dieno­[3,2-*c*]­pyrazol-29-oic Acid (**5d**)

Light yellow solid. Yield 24% (Method A), 43% (Method B). Mp. 273–278 °C. ^1^H NMR (600 MHz, DMSO-*d*_6_) δ 12.21 (bs, 1H), 7.99 (d, *J* = 8.4 Hz, 2H), 7.63 (d, *J* = 8.4 Hz, 2H), 7.40 (s, 1H), 5.50 (s, 1H), 3.58 (d, *J* = 15.5 Hz, 1H), 2.60 (s, 1H), 2.19 (d, *J* = 15.6 Hz, 1H), 2.13 – 2.07 (m, 2H), 1.99 (s, 1H), 1.83 – 1.75 (m, 2H), 1.74 – 1.66 (m, 3H), 1.54 (d, *J* = 10.6 Hz, 1H), 1.42 (d, *J* = 11.3 Hz, 2H), 1.38 (s, 3H), 1.32 – 1.22 (m, 3H), 1.11 (s, 3H), 1.10 (s, 3H), 1.06 (s, 3H), 1.00 (s, 3H), 0.97 (s, 3H), 0.78 (s, 3H). ^13^C NMR (151 MHz, DMSO-*d*_6_) δ_C_ 198.54, 177.65, 170.16, 146.11, 145.60, 138.69, 132.96, 130.07, 127.40, 118.14, 114.30, 111.86, 59.52, 53.37, 48.07, 44.64, 43.07, 40.77, 37.48, 36.79, 34.21, 31.57, 31.23, 30.35, 29.32, 28.46, 27.80, 26.17, 25.81, 22.85, 22.61, 17.89, 17.82, 15.41. HRMS (ESI-MS) *m*/*z* for C_38_H_48_N_3_O_3_^+^ [M + H]^+^ calc. 594.3690; found 594.3689. Purity determined by ^1^H-qNMR was 92.7 ± 0.6% (n = 3).

#### (20β)-2′-(4-Carboxyphenyl)-11-oxo-2′*H*-oleana-2,12-dieno­[3,2-*c*]­pyrazol-29-oic Acid (**5e**)

White solid. Yield 23% (Method A), 46% (Method B). Mp. 278–280 °C. ^1^H NMR (600 MHz, DMSO-*d*_6_) δ_H_ 13.03 (bs, 1H), 12.43 (bs, 1H), 8.05 (d, *J* = 8.3 Hz, 2H), 7.51 (d, *J* = 8.3 Hz, 2H), 7.37 (s, 1H), 5.50 (s, 1H), 3.58 (d, *J* = 15.4 Hz, 1H), 2.60 (s, 1H), 2.19 (d, *J* = 15.5 Hz, 1H), 2.14 – 2.06 (m, 3H), 1.79 (dd, *J* = 19.5, 12.3 Hz, 2H), 1.74 – 1.66 (m, 3H), 1.53 (d, *J* = 10.6 Hz, 1H), 1.42 (d, *J* = 11.8 Hz, 2H), 1.38 (s, 3H), 1.22 – 1.16 (m, 2H), 1.11 (s, 3H), 1.10 (s, 3H), 1.06 (s, 3H), 1.01 (s, 3H), 0.97 (s, 3H), 0.78 (s, 3H). ^13^C NMR (151 MHz, DMSO-*d*_6_) δ_C_ 198.58, 177.67, 170.16, 166.58, 145.87, 145.38, 138.27, 131.25, 129.68, 129.20, 127.42, 114.00, 59.55, 53.43, 48.08, 44.66, 40.78, 37.51, 36.85, 34.24, 31.58, 31.26, 30.36, 29.28, 28.47, 27.81, 26.18, 25.82, 22.86, 22.50, 17.87, 15.42. HRMS (ESI-MS) *m*/*z* for C_38_H_49_N_2_O_5_^+^ [M + H]^+^ calc. 613.3636; found 613.3636. Purity determined by ^1^H-qNMR was 96.5 ± 0.8% (n = 3).

#### (20β)-2′-(4-Carbamoylphenyl)-11-oxo-2′*H*-oleana-2,12-dieno­[3,2-*c*]­pyrazol-29-oic Acid (**5f**)

White solid. Yield 33% (Method A), 60% (Method B). Mp. > 300 °C. ^1^H NMR (600 MHz, DMSO-*d*_6_) δ_H_ 12.21 (bs, 1H), 8.04 (m, *J* = 86.6 Hz, 3H), 7.44 (m, 4H), 5.50 (s, 1H), 3.57 (d, *J* = 14.3 Hz, 1H), 2.59 (s, 1H), 2.19 (d, *J* = 13.7 Hz, 1H), 2.10 (s, 2H), 1.92 (s, 1H), 1.75 (d, *J* = 53.5 Hz, 5H), 1.52 (s, 2H), 1.38 (s, 3H), 1.11 (s, 3H), 1.10 (s, 3H), 1.06 (s, 3H), 1.00 (s, 3H), 0.97 (s, 3H), 0.78 (s, 3H). ^13^C NMR (151 MHz, DMSO-*d*_6_) δ_C_ 198.58, 177.63, 170.15, 167.08, 145.28, 144.52, 138.04, 134.66, 128.83, 127.84, 127.40, 113.83, 59.54, 53.43, 48.06, 44.64, 43.06, 40.76, 37.49, 36.87, 34.23, 31.56, 31.25, 30.36, 29.24, 28.46, 27.81, 26.16, 25.81, 22.86, 22.46, 17.86, 15.40. HRMS (ESI-MS) *m*/*z* for C_38_H_50_N_3_O_4_^+^ [M + H]^+^ calc. 612.3796; found 612.3795. Purity determined by ^1^H-qNMR was 94.1 ± 0.6% (n = 3).

#### (20β)-11-Oxo-2′-(4-sulfamoylphenyl)-2′*H*-oleana-2,12-dieno­[3,2-*c*]­pyrazol-29-oic Acid (**5g**)

Beige solid. Yield 27% (Method A), 69% (Method B). Mp. 250–255 °C. ^1^H NMR (600 MHz, DMSO-*d*_6_) δ_H_ 7.93 (d, *J* = 9.1 Hz, 2H), 7.60 (d, *J* = 8.5 Hz, 2H), 7.53 (bs, 2H), 7.37 (s, 1H), 5.50 (s, 1H), 3.58 (d, J = 15.4 Hz, 1H), 2.60 (s, 1H), 2.19 (d, J = 15.5 Hz, 1H), 2.15 – 2.06 (m, 2H), 1.83 – 1.75 (m, 2H), 1.70 (m, 3H), 1.54 (d, J = 10.9 Hz, 1H), 1.42 (d, *J* = 11.4 Hz, 2H), 1.38 (s, 3H), 1.35 – 1.26 (m, 3H), 1.22 – 1.16 (m, 2H), 1.10 (s, 6H), 1.06 (s, 3H), 1.01 (s, 3H), 0.98 (s, 3H), 0.78 (s, 3H). ^13^C NMR (151 MHz, DMSO-*d*_6_) δ_C_ 198.53, 177.64, 170.13, 145.43, 144.88, 144.42, 138.36, 129.66, 127.42, 126.16, 114.07, 67.37, 59.52, 53.40, 48.05, 44.64, 43.06, 40.78, 38.08, 37.49, 36.82, 34.23, 31.56, 31.19, 30.36, 29.81, 29.32, 28.41, 27.81, 26.17, 25.81, 23.21, 22.85, 22.40, 17.90, 17.86, 15.38. HRMS (ESI-MS) *m*/*z* for C_37_H_50_N_3_O_5_S^+^ [M + H]^+^ calc. 648.3466; found 648.3462. Purity determined by ^1^H-qNMR was 92.8 ± 0.6% (n = 3).

### *In Vitro* Studies

#### Inhibitory Activity against PTP1B and TCPTP

The methodology for obtaining the recombinant proteins *h*PTP1B_1–400_, *h*PTP1B_1–285_, and *h*TCPTP_1–415_, as well as the determination of IC_50_ values and enzymatic kinetics for GA derivatives and positive controls, was conducted following the procedures established in previous studies performed by our group.^14,34–36,96,97^ Briefly, the newly synthesized compounds and positive controls were dissolved in DMSO or Tris buffer solution (50 mM, pH 6.8). Aliquots of 0–10 μL of the test compounds (triplicate) were taken and incubated for 15 min at 37 °C with 85 μL of Tris buffer solution (50 mM, pH 6.8) containing PTP1B enzyme and 5 μL of *p*-nitrophenyl phosphate substrate (pNPP, 10 mM). The absorbance of the hydrolysis product, *p*-nitrophenol (pNP), was measured at 405 nm using a 96-microplate absorbance reader (Accuris SmartReader 96). The inhibitory activity was determined according to eq 1.

$$\%\mathrm{enzyme}=\left(\frac{A_{405b}}{A_{405c}}\right)\times 100$$ 1

Where: % enzyme is the percentage of inhibition for PTP1B or TCPTP

A_405c_: Is the corrected absorbance of the blank (A_405initial_ – A_405final_)

A_405b_: Is the corrected absorbance of the compounds (A_405control_ – A_405compound_)

The IC_50_ was calculated using regression analysis, eq 2:

$$\%\mathrm{inhibition}=\frac{A_{100}}{1+{\left(\frac{I}{{\mathrm{IC}}_{50}}\right)}^{s}}$$ 2

Where: A_100_: Maximum inhibition

I: Inhibitor concentration

IC_50_: Concentration required to inhibit enzymatic activity by 50% ± SD

s: Hill slope

Assays to determine enzyme kinetic parameters and inhibition mechanisms against PTP1B_1–400_ were conducted under the same experimental conditions as the IC_50_ assays, but with variable concentrations of pNPP ranging from 0.1 mM to 0.5 mM in increments of 0.1 mM. Five increasing concentrations of each inhibitor were tested, according to their previously determined IC_50_ values. The negative control was prepared by omitting the inhibitor in the presence of the enzyme and substrate. The Supporting Information includes detailed eqs (E1–E5) used to determine the kinetic parameters and mechanisms involved in PTP1B inhibition.

#### Cytotoxic Assessment against HFF-1 Cells

Normal Human Foreskin Fibroblast HFF-1 cells (ATCC: SCRC-1041) were seeded (3 × 104 cells/well) in 96-well flat-bottomed microplates with DMEM culture medium supplemented with culture medium supplemented with 10% heat-inactivated FBS and 100 U/mL of penicillin plus 100 mg/mL of streptomycin (Invitro, Mexico City, Mexico) and allowed to adhere for 24 h at 37 °C in 5% CO_2_. Cells were exposed to compounds at a concentration range of 0.78 μM to 100 μM, followed by an additional 48-h incubation. All assays were carried out in triplicate. Cell proliferation was determined by the Sulforhodamine B assay, and the half-maximal cytotoxic concentration (CC_50_) was determined by Probit analysis.

### Insulin-Resistant HepG2 Cell Assay

#### Cell Culture

HepG2 cells were obtained directly from ATCC (HB-8065) and cultured in DMEM with low glucose, supplemented with fetal bovine serum, 100 U/mL penicillin G, and 100 mg/mL streptomycin. The media was freshly prepared and sterilized by filtration. Cells were maintained with a maximum of 10 passages before being discarded. For cell counting, the cells were washed twice with phosphate-buffered saline (PBS), trypsinized, and counted using a Neubauer chamber. For the insulin-resistant model, 75,000 cells per well were seeded in 6-well plates at 37 °C in 5% CO_2_ and cultured for 72 h. Subsequently, insulin (0.1 μM) was added to induce insulin resistance for 24 h. After this period, inhibitors were incubated for 24 h, followed by a final 30 min insulin stimulation (0.1 μM) before protein extraction. For the normal model, 75,000 cells per well were seeded in 6-well plates at 37 °C in 5% CO_2_ and cultured for 96 h. After that, the cells were incubated with the inhibitors for 24 h, followed by protein extraction.

#### Cell Viability of HepG2 with Crystal Violet

A 96-well plate was seeded with 3,000 HepG2 cells per well and incubated for 72 h. Subsequently, the cells were treated with test compounds at various concentrations for 24, 48, and 72 h. After incubation, the medium was removed, and 100 μL of 1% glutaraldehyde was added for 15 min. The glutaraldehyde was then removed, and 50 μL of 0.5% crystal violet (dissolved in 25% methanol) was added for 30 min. The crystal violet staining solution was subsequently removed, and the plate was thoroughly rinsed with tap water. The precipitate was dissolved in a 10% acetic acid solution, mixed for 15 min, and the absorbance was measured at 590 nm using a plate reader (xMark Microplate Spectrophotometer, Bio-Rad).

#### Protein Extraction and Western Blotting

Treated cells were washed twice with 1 mL of PBS, and proteins were extracted using 300 μL of a buffer solution containing 10% sodium deoxycholate in 10% TEAB, supplemented with a protease and phosphatase inhibitor cocktail. The extracts were heated at 80 °C for 5 min and dissociated by repeated passages through an insulin syringe. The cell extracts were centrifuged at 15,000 rpm for 15 min at 4 °C, and the supernatant was collected. Protein quantification was performed using the bicinchoninic acid assay, and aliquots were prepared for Western blotting. SDS-PAGE was conducted, and proteins were transferred onto a nitrocellulose membrane. The membrane was blocked with 10% skim milk in TBST for 1 h and incubated overnight with primary antibodies (1:400 dilution). Secondary antibodies conjugated to horseradish peroxidase were diluted 1:2,000, and the membranes were incubated for 1 h at room temperature. Blots were visualized using the ChemiDoc MP (Bio-Rad), and densitometric analysis was performed with ImageJ (U.S. National Institutes of Health). Statistical analyses were conducted using GraphPad Prism 8.0.

#### GC-MS Untargeted Metabolomic Analysis

The treated HepG2 cells were washed twice with 1 mL of PBS, and the liquid was entirely removed by pipetting. Posteriorly, 1 mL of ice-cold methanol containing 2.5 μL of internal standard, consisting of tridecanoic acid (0.1 mg/mL), was added, and the mixture was bath sonicated for 2 min. All the extract was recovered in microcentrifuge tubes, thoroughly vortexed, and then centrifuged for 10 min at 15,000 rpm at 4 °C. The supernatant was recovered and dried overnight using a SpeedVac (SPD 121P, Thermo Scientific). 40 μL of 20 mg/mL methoxamine hydrochloride was added under nitrogen flow and incubated for 90 min at 37 °C. Posteriorly, the tube was centrifuged, and the supernatant was fully recovered and transferred to an insert tube with 40 μL of MBSTFA 1% TMCS, vortexed and incubated for 30 min. One μL of this extract was injected into a GC/MS (Agilent 5977*A*/7890B, Santa Clara, CA, USA) system with HP5-MS (Agilent) column with helium 99.9999% purity under the following parameters: splitless, flow 1 mL/min, electron ionization, with a range of 50–500 *m*/*z*. The chromatography consisted of a 1 min hold at 60 °C, followed by a ramp of 10 °C/min to 325 °C, and a final hold time of 10 min. Raw data was transformed with Agilent Mass Hunter to mz data, and deconvolution and alignment were performed with Mzmine2.0. Univariate and multivariate analyses were performed with Metaboanalyst 6.0 and GraphPad.

### *In Vivo* Studies

#### Animals

Healthy six-week-old male CD-1 mice, weighing between 30 and 40 g, were maintained at a temperature of 25 ± 2 °C, under a 12-h light/dark cycle and 45–65% humidity throughout the experimental period. The mice were provided with a rodent pelleted diet (Harlan Laboratories, Indianapolis, USA) and had access to water *ad libitum*. Sixteen hours before the experiment, all mice were fasted but still had free access to water. All animal procedures were conducted in accordance with the Mexican Federal Regulations for Animal Experimentation and Care (SAGARPA, NOM-062-ZOO-1999, Mexico) and received approval from the Institutional Animal Care and Use Committee, following guidelines outlined in the US National Institutes of Health publication (No. 85–23, revised 1985).

#### Induction of Experimental Diabetes

Streptozotocin (STZ) was dissolved in citrate buffer (pH 4.5), while nicotinamide was dissolved in a normal physiological saline solution. The noninsulin-dependent diabetes (NIDD) mouse model was induced in overnight-fasted mice through a single intraperitoneal injection of STZ (100 mg/kg).^98^ Nicotinamide was administered 15 min prior, at a dose of 20 mg/kg via intraperitoneal injection. Hyperglycemia was confirmed 1 week later by measuring blood glucose levels with a glucometer (Accu-Chek Performa; Roche). Mice exhibiting blood glucose concentrations higher than 140 mg/dL were selected for antidiabetic screening.

#### Antidiabetic Assay

Animals were categorized into five groups, each consisting of four mice (n = 4). The experimental groups received an oral suspension of compounds **4b**, **4g**, and **5g**, prepared in a 10% Tween 20/water solution, at a dosage of 50 mg/kg body weight. A control group was treated with only the 10% Tween 20 solution. The final group was administered pioglitazone at a dose of 45 mg/kg, which is recognized as a reference drug for insulin sensitization. To evaluate glucose levels, blood samples were collected from the tails of the mice at 0, 30, 60, 90, and 120 min after the administration of the vehicle, test compound, or drug. Blood glucose concentration was measured using a commercial glucometer (Accu-Chek, Performa; Roche) and the enzymatic glucose dehydrogenase method. The percentage variation in glycemia was calculated by comparing the selected postadministration glycemia (G_*x*_) with the initial value (G_0_) using the following formula:

$$\%\mathrm{variation}\;\mathrm{of}\;\mathrm{glycemia}=[(G_{x}-G_{0})/G_{0}]\times 100$$

where G_0_ represents the initial glycemia values and Gx indicates the glycemia values at +30, + 60, + 90, and +120 min, respectively.

#### Oral Glucose Tolerance Test (OGTT)

To determine the hypoglycemic effect of the compounds on nondiabetic animals, the compounds **4b**, **4g**, and **5g** were administered intragastrically. Pioglitazone was used as a positive control. After 30 min, five mice/group (CD-1) were subjected to OGTT. The test was performed using glucose (2 g/kg) as substrate.

#### Insulin Tolerance Test (ITT)

To evaluate the ability of GA derivatives to enhance insulin sensitivity in STZ-induced diabetic CD-1 mice, compounds **4b**, **4g**, and **5g** were tested. Glycyrrhetinic acid (GA) and Pioglitazone served as positive controls. The GA derivatives and the positive controls were administered to the diabetic animals, followed by the administration of human rapid-acting insulin (0.75 IU) after 30 min. Subsequently, glucose levels were measured every 15 min.

#### Statistical Analysis

The statistical analysis was performed using GraphPad 8.0 Prism (GraphPad Software 8, San Diego, Ca, USA). Data were presented as mean ± SEM. Studen’s *t* test were used to assess between-group differences, and Multiple-group comparisons were performed with one wat analysis of variance followed by Tukey Kramer’s post hoc test. The results were considered statistically significant at *p* < 0.05.

### *In Silico* Studies

#### Molecular Modeling of GA Derivatives

The pNPP and GA derivatives **4a**, **4b**, **4e**, **4g** and **5g** were constructed in AVOGADRO^99,100^ (version 1.2.0) (http://avogadro.cc/). The GA derivatives were constructed by systematically modifying the structure of GA (with deposition number 1169430 ^101^), which was retrieved from the Cambridge Crystallographic Data Base (https://www.ccdc.cam.ac.uk/). Subsequently, the protonation states of all compounds were fixed, assuming a pH of 7.4, and the 3D geometries were optimized using the Universal Force Field (UFF) in AVOGADRO and then saved as *.sdf files. Afterward, a single *.sdf file, which contains all the GA ligands, was created in DataWarrior^102^ (version 05.05.00) (https://openmolecules.org/datawarrior/). Consensus Log *P* values were calculated with SwissADME^40,41^ (http://www.swissadme.ch/).

#### PTP1B_1–400_-pNPP Model

This model was constructed according to our previous work.^103^ Briefly, the 3D structure of PTP1B_1–400_ was retrieved from the AlphaFold Protein Structure Database, developed by DeepMind and EMBL-EBI (https://alphafold.ebi.ac.uk/) with the UniProt code P18031 and AlphaFold code AF–P18031-F1. Subsequently, the 3D structure was submitted to MDS using the AMBER11 ^60^ (also known as AMBER ff99sb*-ILDN) force field to obtain a folding with biological relevance of the unstructured C-terminal zone. To do so, 270 ns of MDS were performed for PTP1B_1–400_. Afterward, the model was refined by running the *md_refine* macro, which uses the YASARA2 ^59^ force field. The snapshot with the minimum energy and maximum quality score was selected. For the construction of the PTP1B_1–400_-pNPP complex, utilized as the uncompetitive model for docking simulations, the ligand pNPP was first docked into the catalytic site of the PTP1B_1–400_ structure using Vina,^104^ which is integrated into YASARA Structure. A cube simulation cell with an extension of 6 Å was created around residue Cys^215^, and the resulting file was saved in *.sce format. Concurrently, the pNPP ligand was constructed as described in the Molecular Docking section. The ligand was then saved in *.sdf format. With the PTP1B_1–400_ structure and the pNPP ligand prepared, molecular docking was performed using the modified *dock_run* macro set for 500 runs. The resulting structure with the best binding energy and the largest population was selected. With the newly created 3D model of the PTP1B_1–400_-pNPP complex, a new MDS was conducted using the AMBER11 force field, with a simulation time of 200 ns. Afterward, the structure was further refined using the *md_refine macro*. The snapshot with the minimum energy and maximum quality score was selected. Lastly, the quality of the PTP1B_1–400‑_pNPP complex was assessed with Molprobity^105^ server (http://molprobity.biochem.duke.edu/) and Swiss Model Structure Assessment^106^ server (https://swissmodel.expasy.org/assess)

#### Molecular Docking

Molecular docking was conducted using AutoDock and Vina, both of which are integrated within the YASARA Structure suite (version 24.10.5). Additionally, GOLD software (version 2024.3.0) was utilized in this study.

For the blind docking simulations, a simulation cell or grid box encompassing the entire PTP1B_1–400_-pNPP protein, with an additional 5 Å extension, was employed. The protein and simulation cell were saved as a *.sce file. Subsequently, molecular docking was performed using Autodock and Vina with the protein (*.sce file) and GA ligands (*.sdf file) through the *dock_runscreening* macro, which had been modified to include 200 runs of the Lamarckian Genetic Algorithm. The resulting binding poses indicated that the compounds primarily bind to three specific sites. Consequently, a site-specific molecular docking was conducted at these identified sites using the following coordinates: x = 10.414, y = −1.707, z = 14.01 (for site 1); x = −8.84, y = 18.65, z = 0.33 (for site 2), and x = −19.56, y = 12.91, z = 35.65 (for site 3). The simulation cell, or grid box, had an extension of 6 Å, resulting in grid dimensions of 60 × 60 × 60 Å, with a spacing of 0.375 Å for all three sites. The search was conducted using the *.sdf file of GA ligands and the *dock_runscreening* macro, which was modified to include 100 runs of the Lamarckian Genetic Algorithm for Autodock. For Vina, 200 runs were performed. Finally, each ligand with the best cluster size and the lowest binding energy was selected for further analysis.

The PTP1B_1–400_-pNPP-ligand complexes generated in YASARA Structure were then exported to GOLD software. Using the GOLD wizard, the proteins were prepared by adding hydrogens and extracting the ligands, which were then further docked at the catalytic site or allosteric site within a 6 Å radius sphere, carried out using the following parameters: 100 genetic algorithm runs and 125,000 operations. CHEMPLP fitness was chosen as the main scoring function, whereas GoldScore fitness was selected as the rescoring function. The dockings were ranked according to the value of the CHEMPLP and GoldScore fitness function.

The binding poses shown in Figures 3 and S44–S47 were derived from the results obtained by Autodock. However, it is important to note that the binding poses obtained from the Vina and GOLD programs were similar.

#### Molecular Dynamics Simulations (MDS)

The protein–ligand complexes were submitted to MDS with YASARA Structure^57,59^ version 24.10.5. The simulations started with an optimization of the hydrogen bonding network to increase the solute stability and a p*K*_a_ prediction to fine-tune the protonation states of protein residues at the chosen pH of 7.4, then NaCl ions were added with a physiological concentration of 0.9%, with an excess of either Na^+^ or Cl^–^ to neutralize the cell. After the steepest descent and simulated annealing minimizations to remove clashes, the simulation was run for 300 ns using the AMBER11 ^60^ force field for the solute, GAFF2 ^107^ and AM1BCC^108^ for ligands and TIP3P^109^ for water. The cutoff was 8 Å for van der Waals forces (the default used by AMBER); no cutoff was applied to electrostatic forces (using the Particle Mesh Ewald algorithm^110^). The equations of motion were integrated using the YASARA default parameters: a multiple-time step of 1.25 fs for bonded interactions and 2.5 fs for nonbonded interactions at a temperature of 310 K and a pressure of 1 atm (NPT ensemble), employing algorithms described in detail previously.^111^ After inspecting the solute RMSD as a function of simulation time, the first 100 ps were considered equilibration time and excluded from further analysis. The binding energy study using the MM-PBSA method was performed by running *the md_analyzebindingenergy* macro, which was previously modified with PBS method at a temperature of 310 K. The RMSD, RMSF, and binding energy plots were generated using the codes available at https://github.com/Franciscoqfb87/MDS-graphs.git

## Supplementary Material



## Notes

Experimental details: ^1^H and ^13^C NMR as well as ESI-MS for compounds **4a**–**g** and **5a**-**g**; binding modes from molecular docking simulations; cell viability of HepG2 cells; western blotting and additional figures and tables as noted in the text (PDF)

## Abbreviations

GC-MS: gas chromatography–mass spectrometry
HFF-1: human foreskin fibroblast
IRS-1: insulin receptor substrate 1
ITT: insulin tolerance test
LepR: leptin receptor
pNPP: *p*-nitrophenylphosphate
PI3K: phosphoinositide 3-kinase
POMPC: proopiomelanocortin
PPAR−γ: peroxisome proliferator-activated receptor gamma
PTP1B: protein tyrosine phosphatase 1B
STAT3: signal transducer and activator of transcription 3
SOCS3: suppressor of cytokine signaling 3
TCPTP: T-cell protein tyrosine phosphatase
SGLT2: sodium-glucose cotransporter 2
T2DM: Type 2 diabetes mellitus
TLC: thin layer chromatography
WAT: white adipose tissue
