# Supplementary material for: Optimization of Indole- and Pyrazole-fused Glycyrrhetinic Acid Derivatives as Potent PTP1B Inhibitors: In Silico, In Vitro, In Vivo, and Metabolomic Studies
Source: ACS Bio Med Chem Au. 2025 Oct 31;6(1):15–35. doi: 10.1021/acsbiomedchemau.5c00164 (PMC12921516; doi:10.1021/acsbiomedchemau.5c00164)
Supplement: Supplementary file 1 [file bg5c00164_si_001.pdf]

Supporting information for

Optimization of Indole- and Pyrazole-fused Glycyrrhetinic Acid Derivatives as Potent PTP1B Inhibitors: In Silico, In Vitro, In Vivo, and Metabolomic Studies

Mitzi López-Sánchez[a,b,c,d], Hannya Mendoza-Mota[b,‡], Ledy De-la-Cruz-Martínez[a,b,f,‡], Félix Matadamas-Martínez[e], Diana Laura Torres-Chacón[f], Rosendo Martínez-Arellano[b,d], Juan Francisco Palacios-Espinosa[b], Jaime Pérez-Villanueva[b], Martin González-Andrade[c,*] José Carlos Páez-Franco[d,*], Julio César Almanza-Pérez[f,*], Francisco Cortés-Benítez[b,*]

[a] Maestría y Doctorado en Ciencias Farmacéuticas, División de Ciencias Biológicas y de la Salud, Universidad Autónoma Metropolitana – Unidad Xochimilco, Ciudad de México 04960, Mexico.

[b] Laboratorio de Síntesis y Aislamiento de Sustancias Bioactivas, Departamento de Sistemas Biológicos, División de Ciencias Biológicas y de la Salud, Universidad Autónoma Metropolitana – Unidad Xochimilco, Ciudad de México 04960, Mexico.

[c] Laboratorio de Biosensores y Modelaje Molecular, Departamento de Bioquímica, Facultad de Medicina, Universidad Nacional Autónoma de México, Ciudad de México 04510, Mexico.

[d] Red de Apoyo a la Investigación, Universidad Nacional Autónoma de México e Instituto Nacional de Ciencias Médicas y Nutrición Salvador Zubirán, Ciudad de México 14080, Mexico.

[e] Unidad de Investigación Médica en Enfermedades Infecciosas y Parasitarias, UMAE Hospital de Pediatría, Centro Médico Nacional Siglo XXI, Instituto Mexicano del Seguro Social, Ciudad de México 06720, Mexico.

[f] Laboratorio de Farmacología, Departamento de Ciencias de la Salud, D.C.B.S., Universidad Autónoma Metropolitana - Unidad Iztapalapa, Ciudad de México 09340, Mexico.

Table of contents

**Figures S1 to S28.** ^{1}H NMR and ^{13}C NMR spectra of compounds **4a-5g**	S1 to S14
**Figure S29 to S42.** Mass spectrum formula report of compounds **4a-5g**	S15 to S21
**Figure S43.** IC_{50} values for compounds **4a-5g** against *h*$PTP1B_{1\text{-}400}$	S22
**Table S1.** Docking scores of GA derivatives against the $PTP1B_{1\text{-}400}$-pNPP complex	S23
**Figure S44.** Predicted binding modes of GA derivatives	S24
**Figure S45.** 2D diagram interaction of compounds **4a**, **4b**, **4e**, **4g**, and **5g** within the site 1 of the $PTP1B_{1\text{-}400}$-pNPP complex	S25
**Figure S46.** 2D diagram interaction of compounds **4a**, **4b**, **4e**, **4g**, and **5g** within the site 2 of the $PTP1B_{1\text{-}400}$-pNPP complex	S25
**Figure S47.** 2D diagram interaction of compounds **4a**, **4b**, **4e**, **4g**, and **5g** within the site 3 of the $PTP1B_{1\text{-}400}$-pNPP complex	S26
**Figure S48.** Cell viability assessment by crystal violet staining on HepG2 cells	S26
**Figure S49.** Effects **4b**, **4g** and **5g**, metformin and ursolic acid on the levels of PTP1B, p-AKT, p-IRS1 and p-STAT3 in HepG2 cells	S27
**Figure S50**. Effects **4b**, **4g** and **5g**, metformin and ursolic acid on the levels of PTP1B, p-AKT, p-IRS1 and p-STAT3 in an insulin resistance model in HepG2 cells.	S28
**Table S2.** Purity of compounds **4a-5g** determined by ^{1}H-qNMR	S30
Equations used to determine the kinetic parameters of PTP1B inhibition	S31

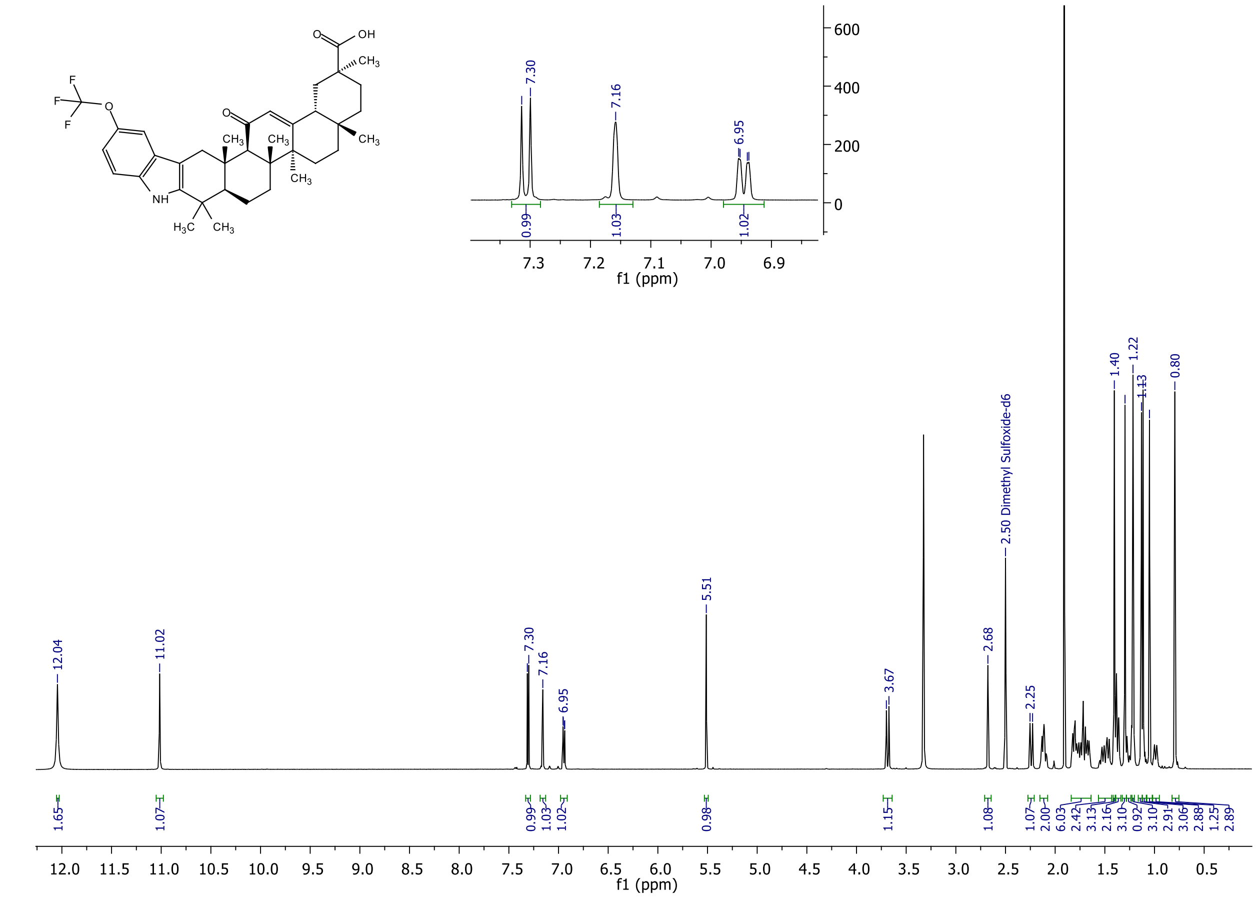

Figure S1. ^{1}H NMR (600 MHz) of compound 4a in DMSO-d_6

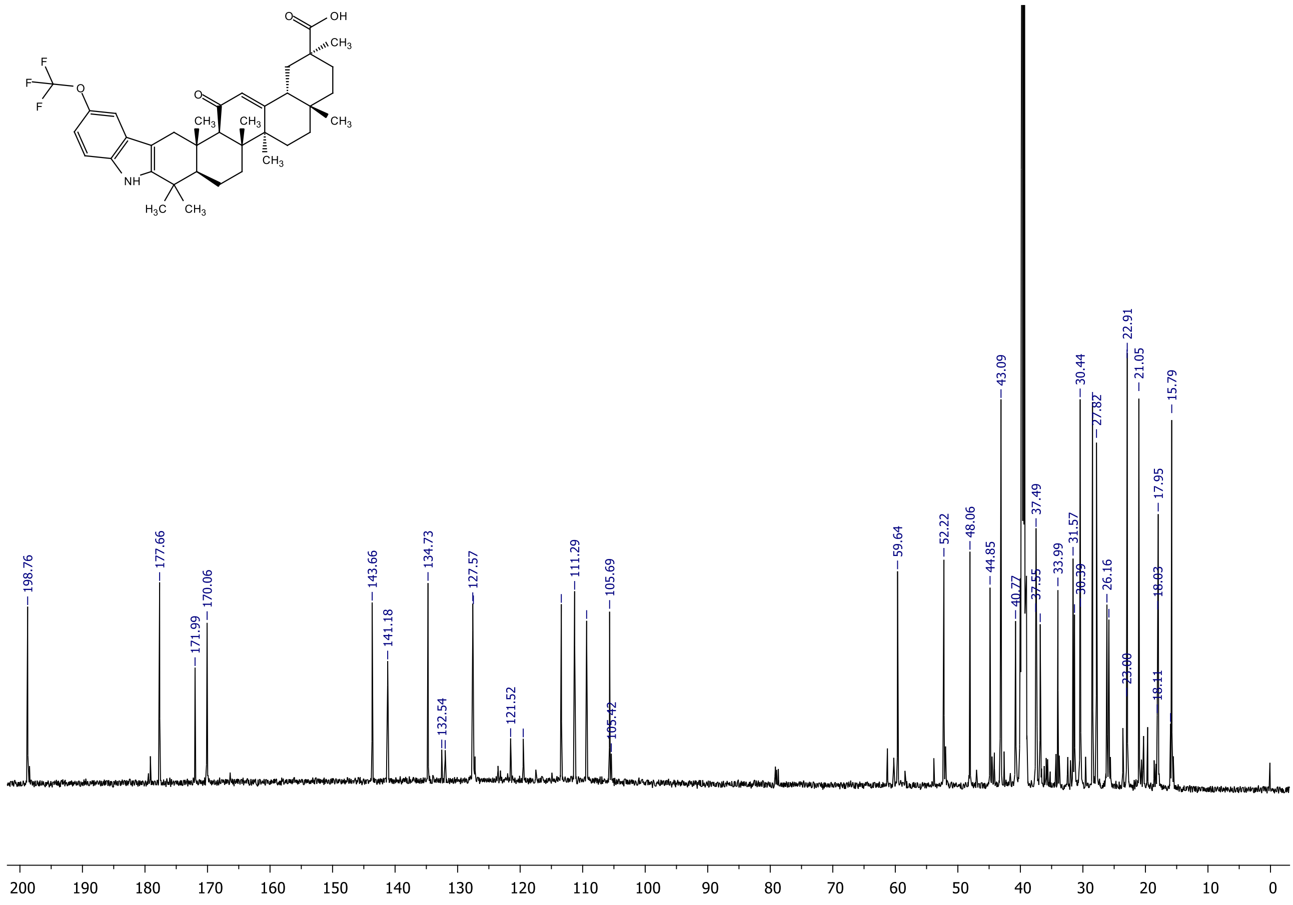

Figure S2. ^{13}C NMR (151 MHz) of compound 4a in DMSO-d_6

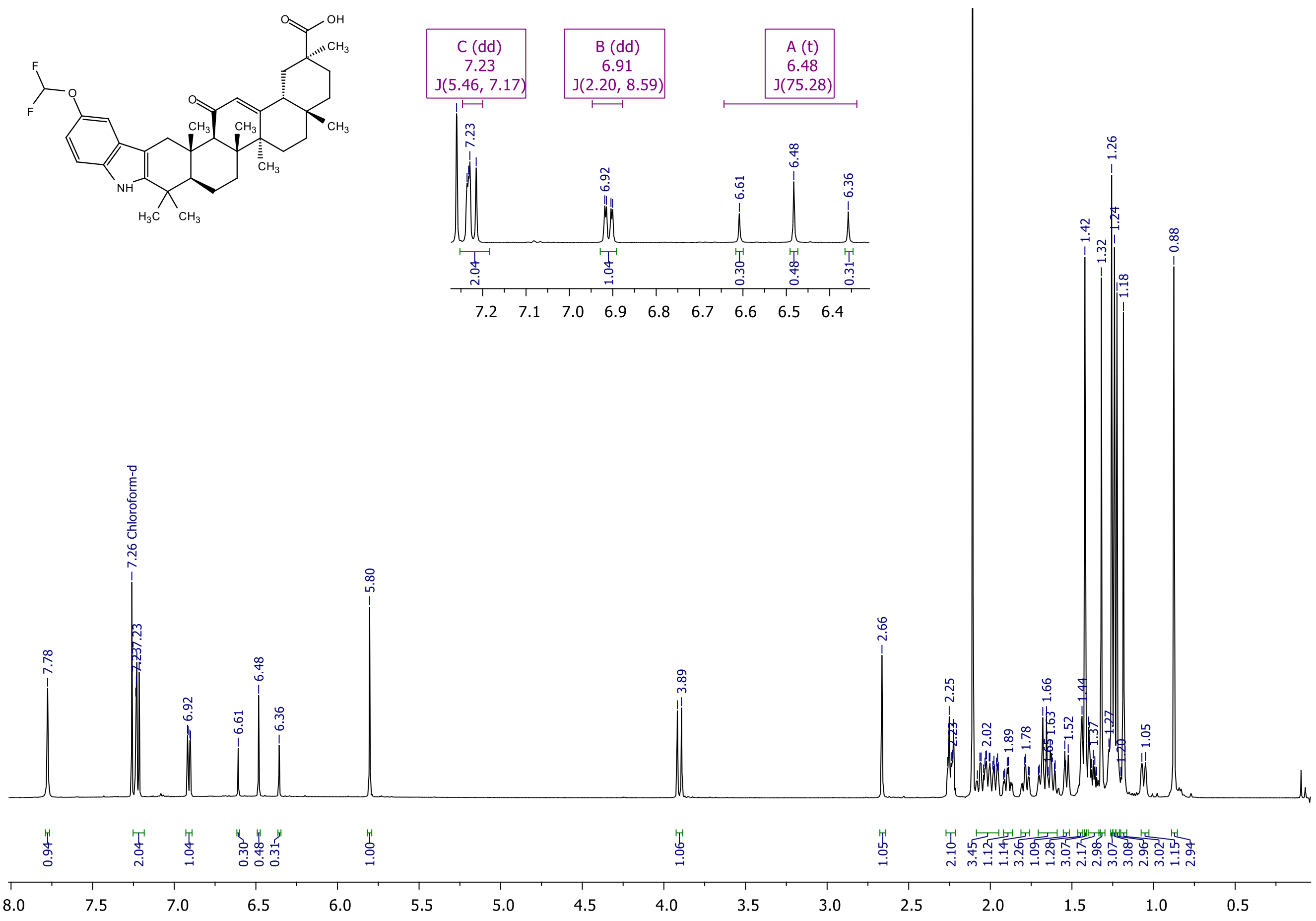

Figure S3. ^{1}H NMR (600 MHz) of compound 4b in $CDCl_3$

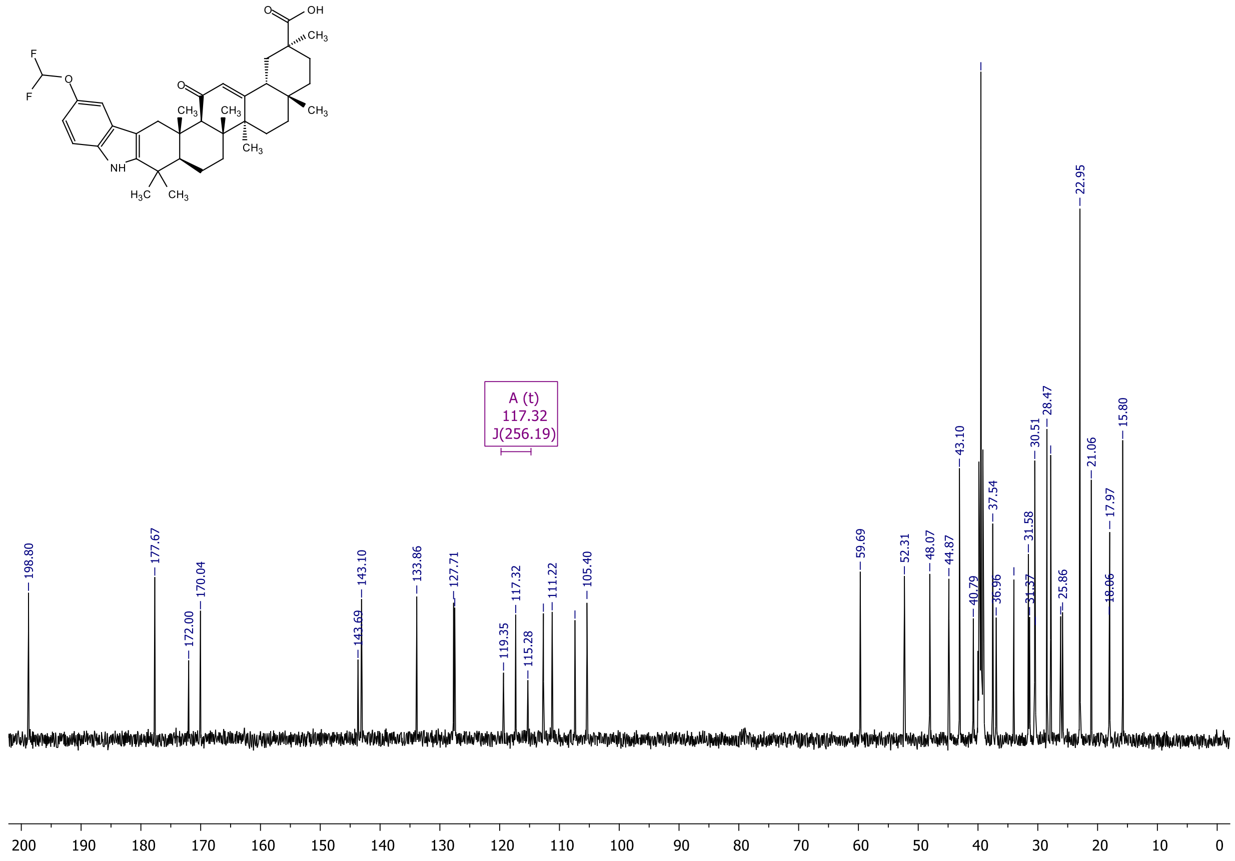

Figure S4. ^{13}C NMR (151 MHz) of compound 4b in DMSO-d_6

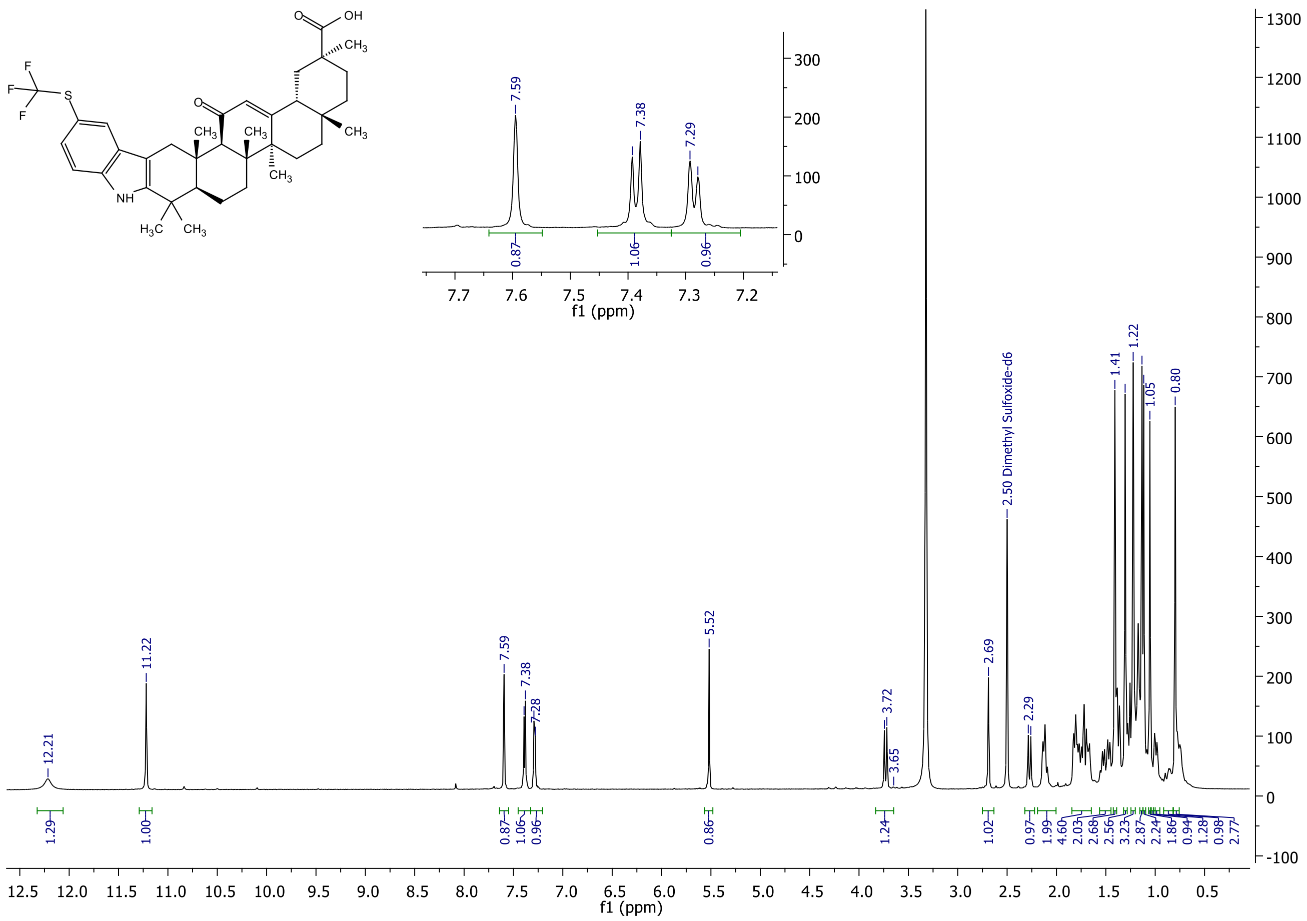

Figure S5. ^{1}H NMR (600 MHz) of compound 4c in DMSO-d$_6$

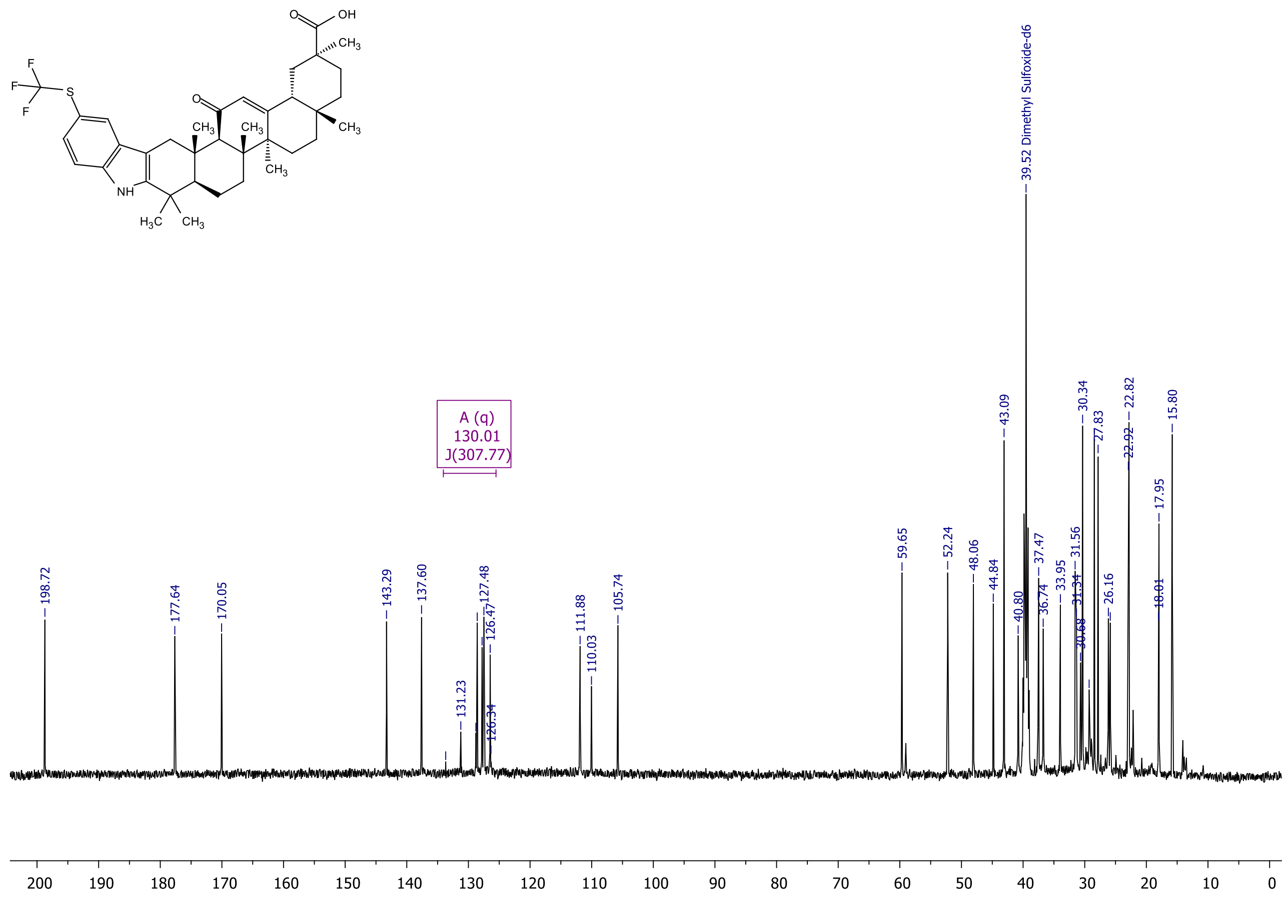

Figure S6. ^{13}C NMR (151 MHz) of compound 4c in DMSO-d$_6$

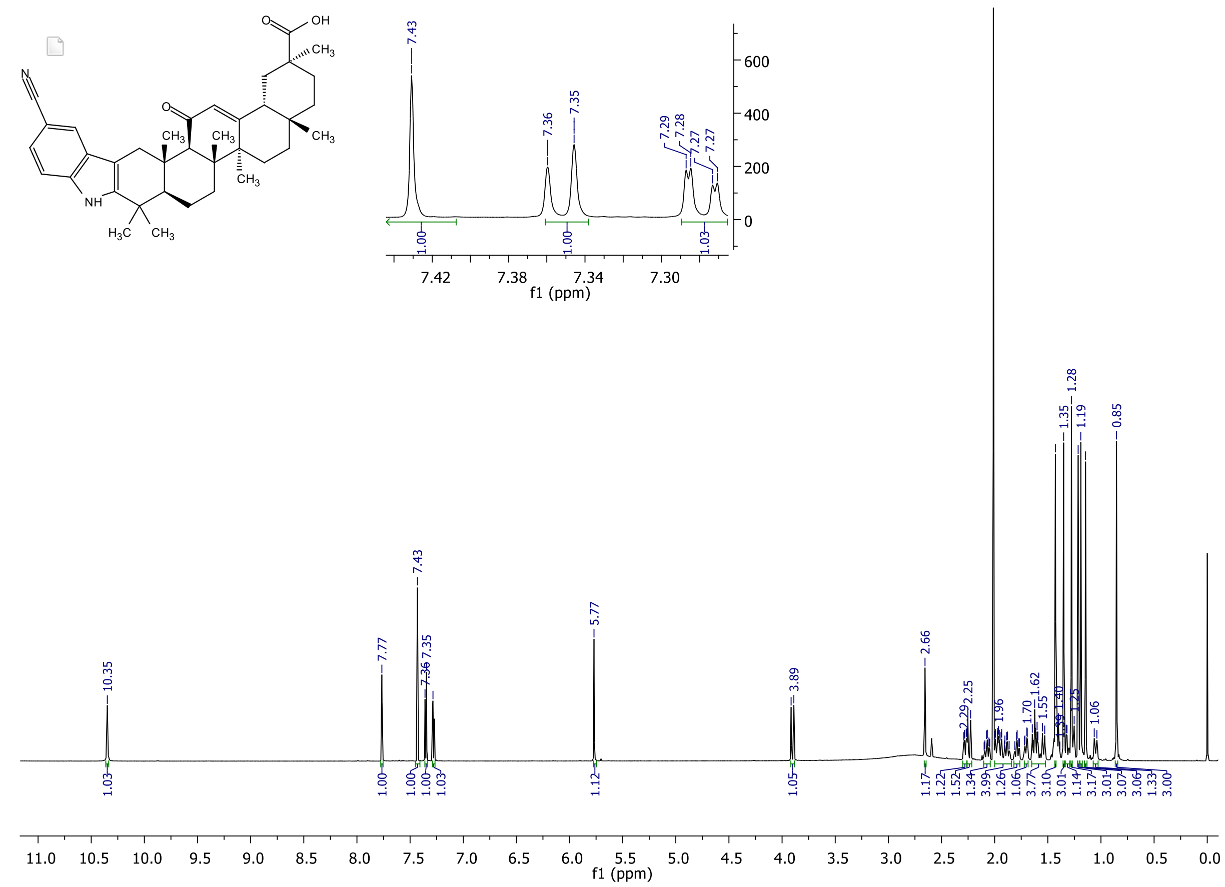

Figure S7. ^{1}H NMR (600 MHz) of compound 4d in DMSO-d_6

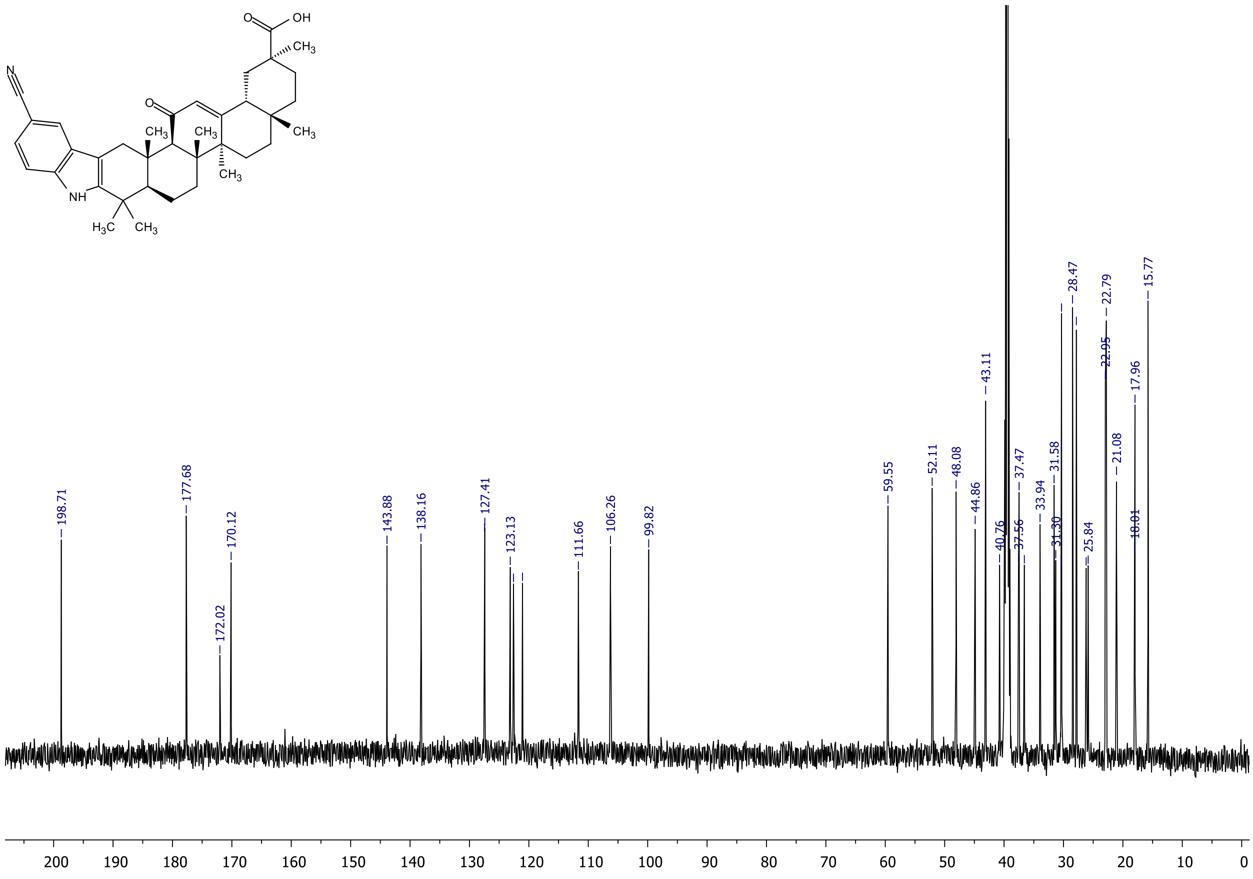

Figure S8. ^{13}C NMR (151 MHz) of compound 4d in DMSO-d_6

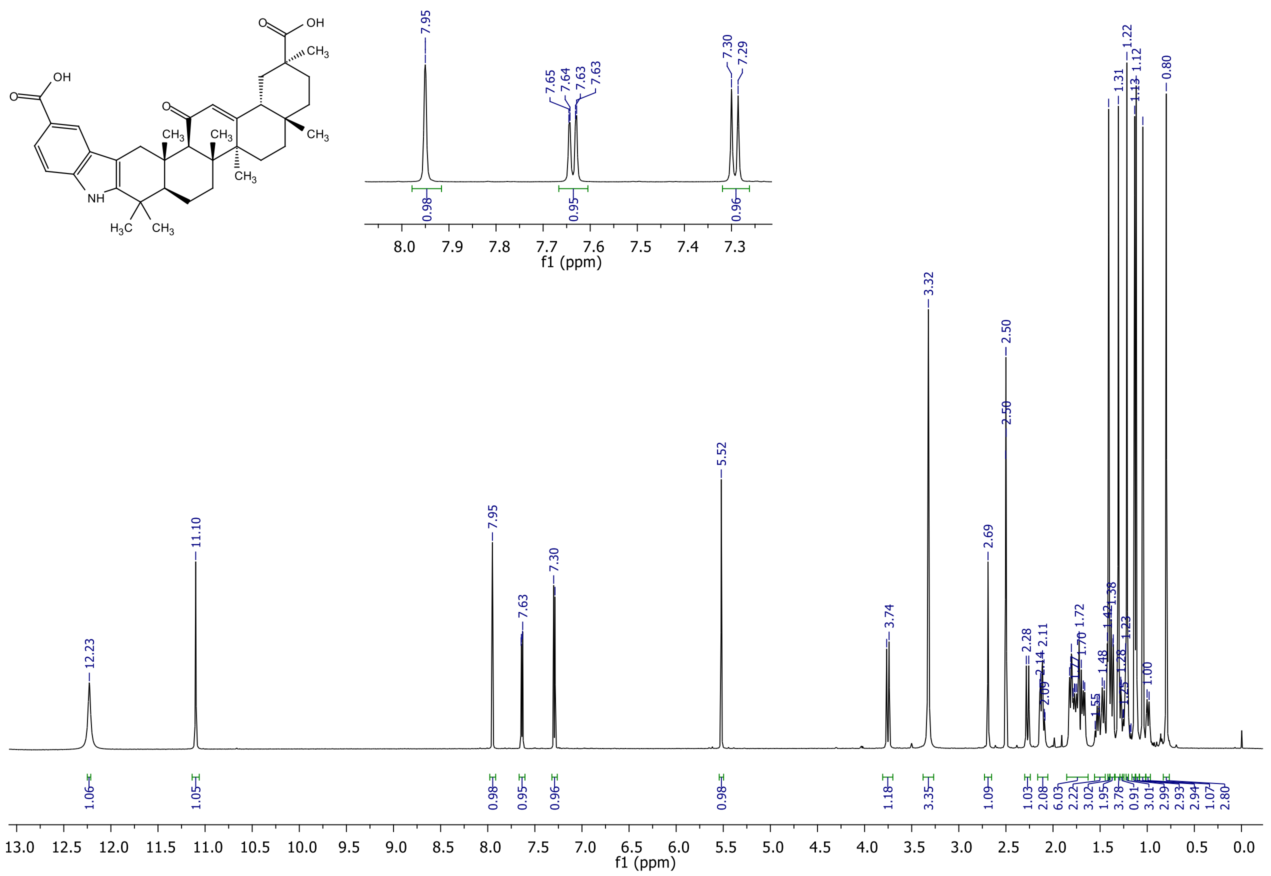

Figure S9. ^{1}H NMR (600 MHz) of compound 4e in DMSO-d_6

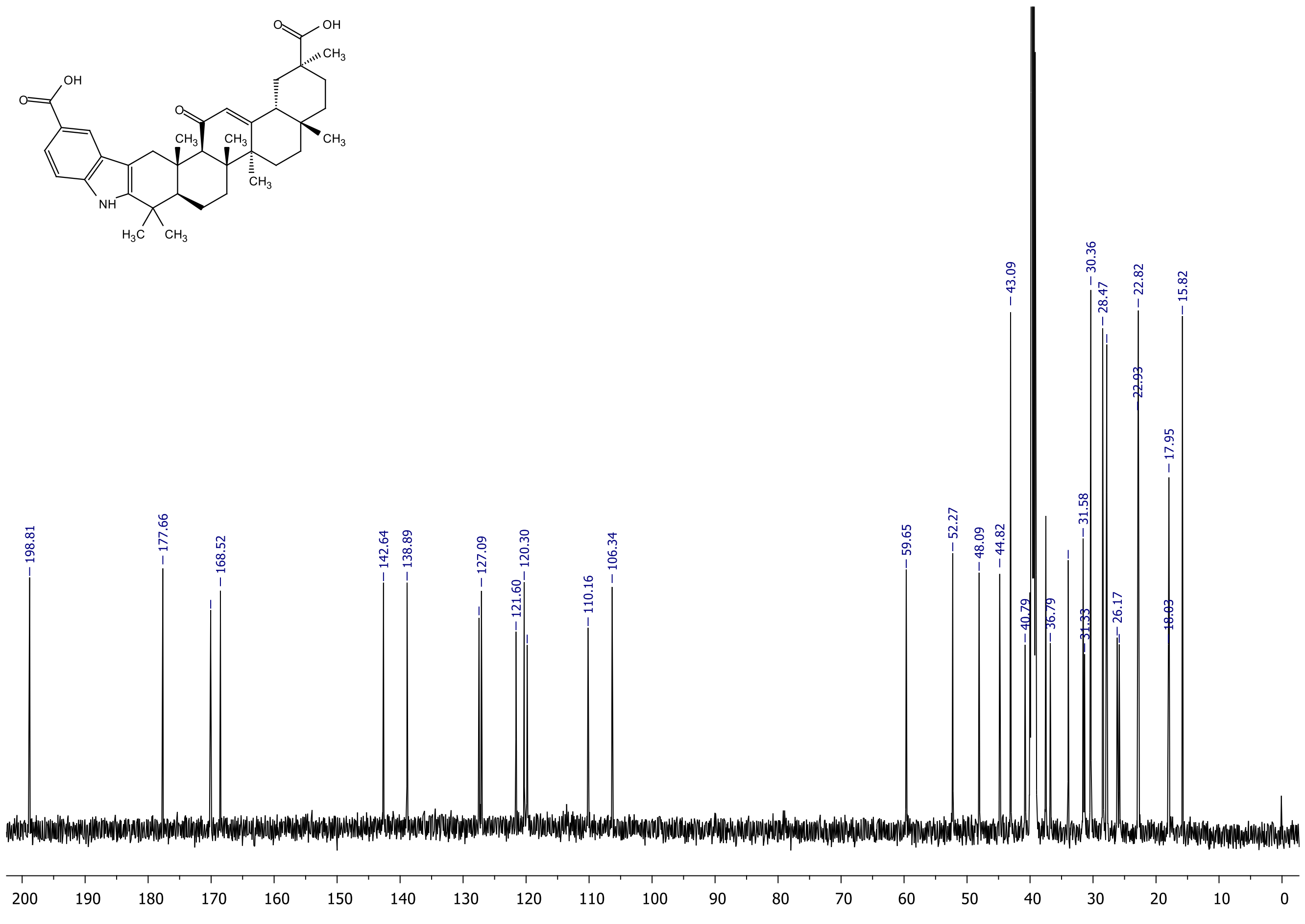

Figure S10. ^{13}C NMR (151 MHz) of compound 4e in DMSO-d_6

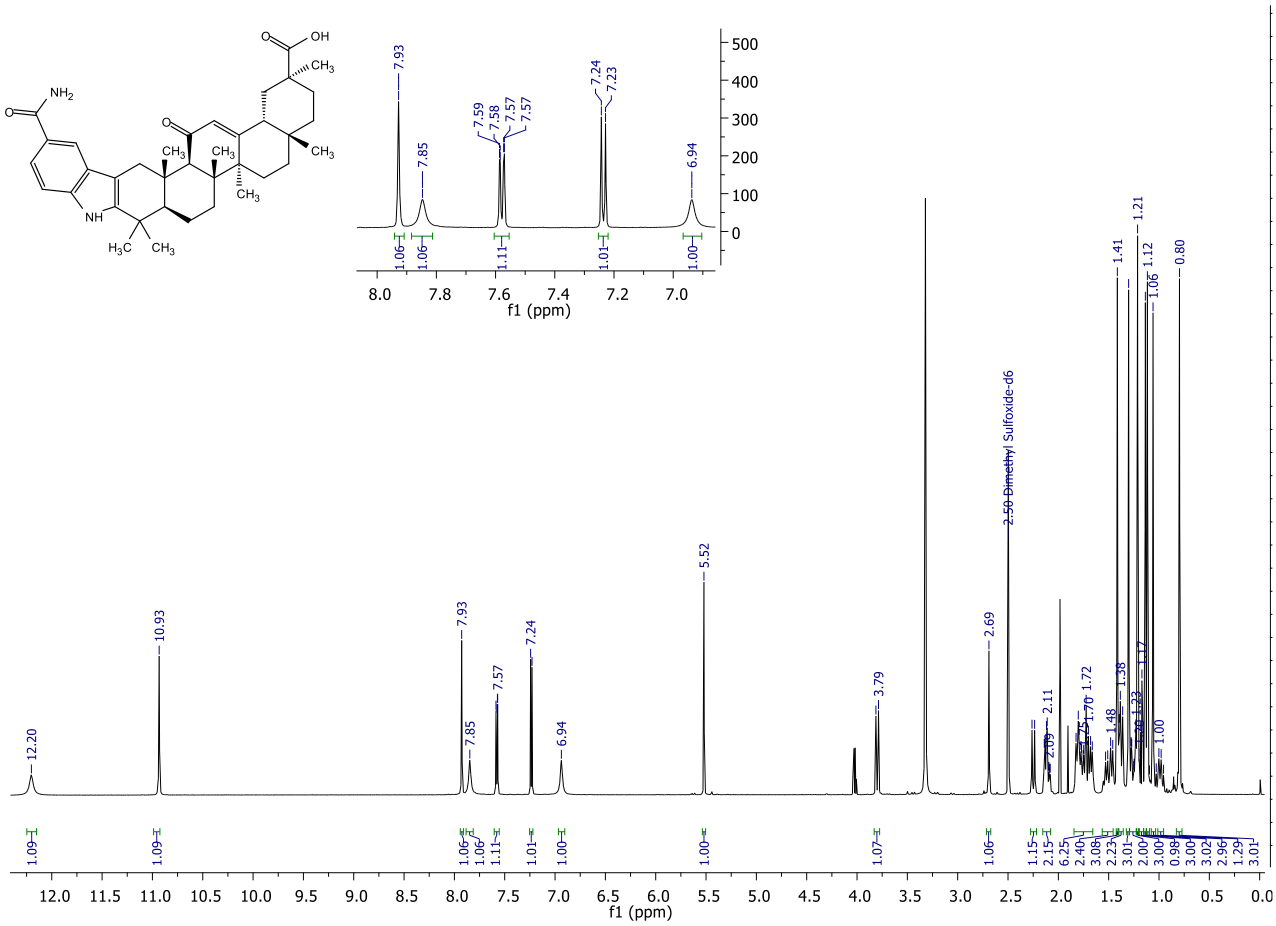

Figure S11. ^{1}H NMR (600 MHz) of compound 4f in DMSO-d_6

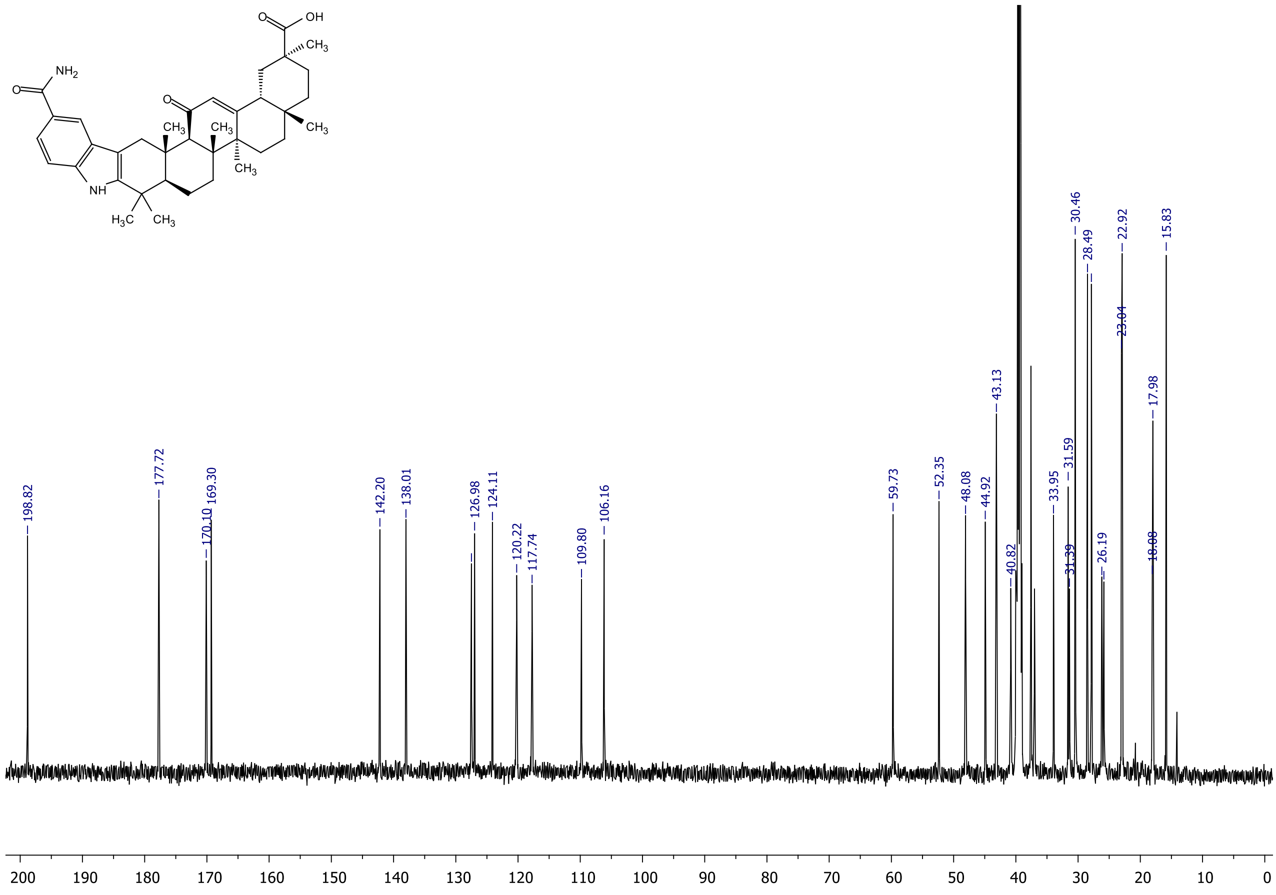

Figure S12. ^{13}C NMR (151 MHz) of compound 4f in DMSO-d_6

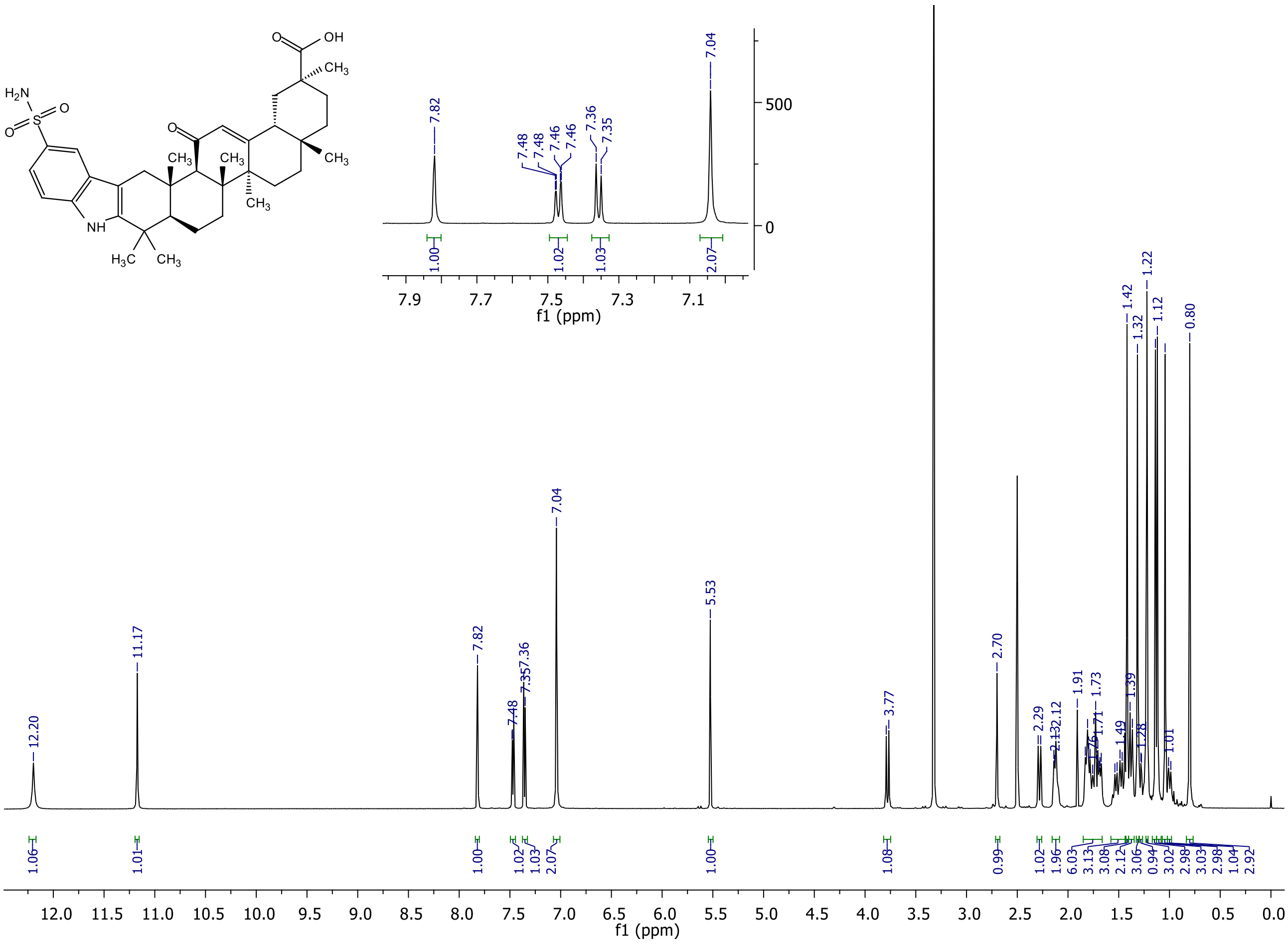

Figure S13. ^{1}H NMR (600 MHz) of compound 4g in DMSO-d_6

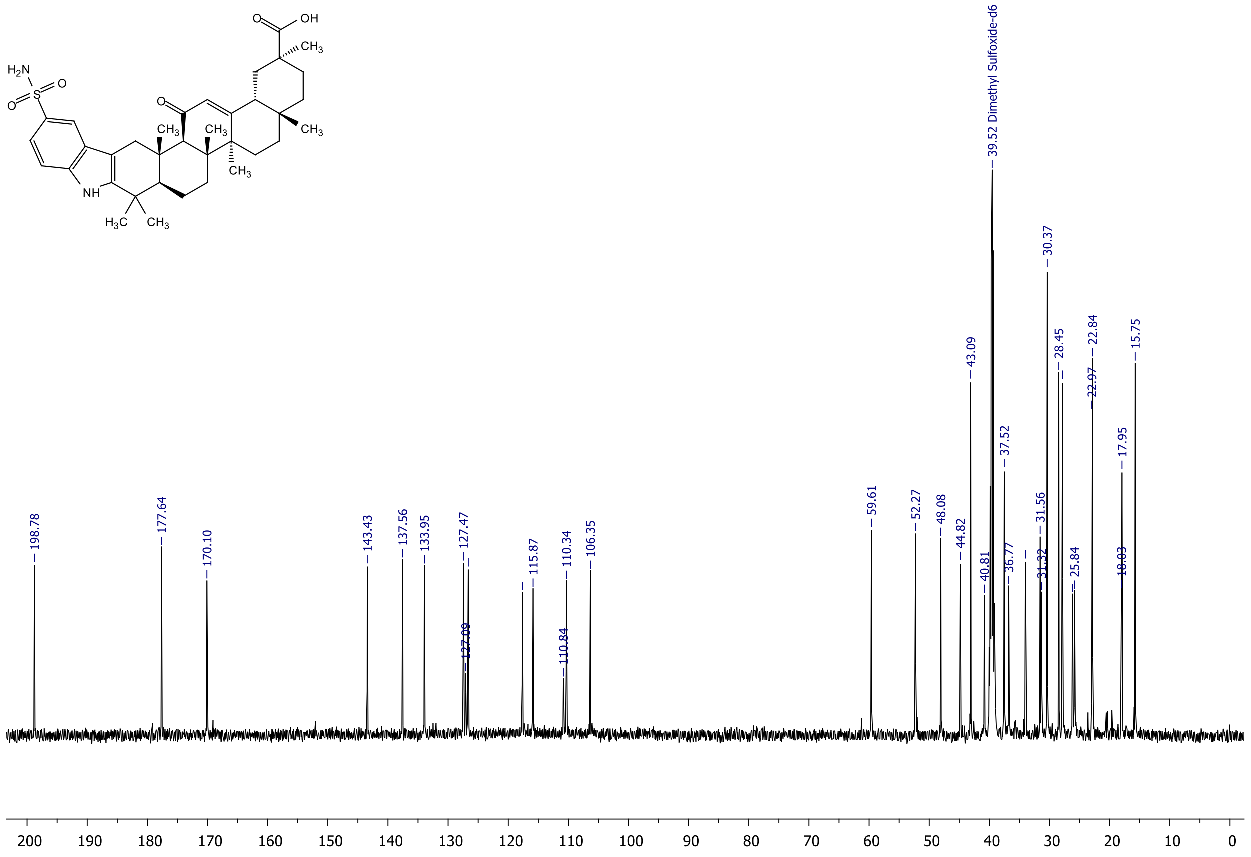

Figure S14. ^{13}C NMR (151 MHz) of compound 4g in DMSO-d_6

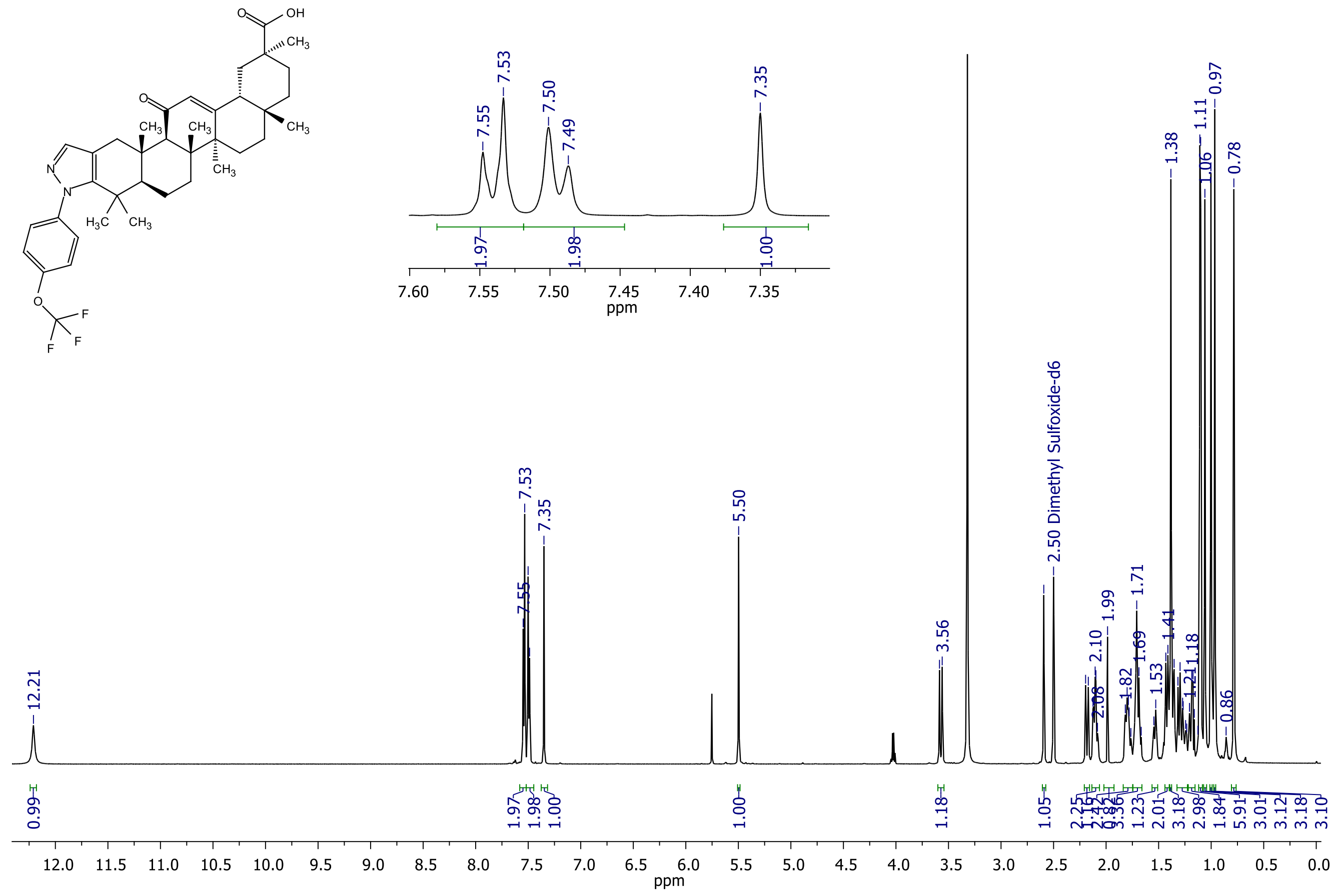

Figure S15. ^{1}H NMR (600 MHz) of compound 5a in DMSO-d_6

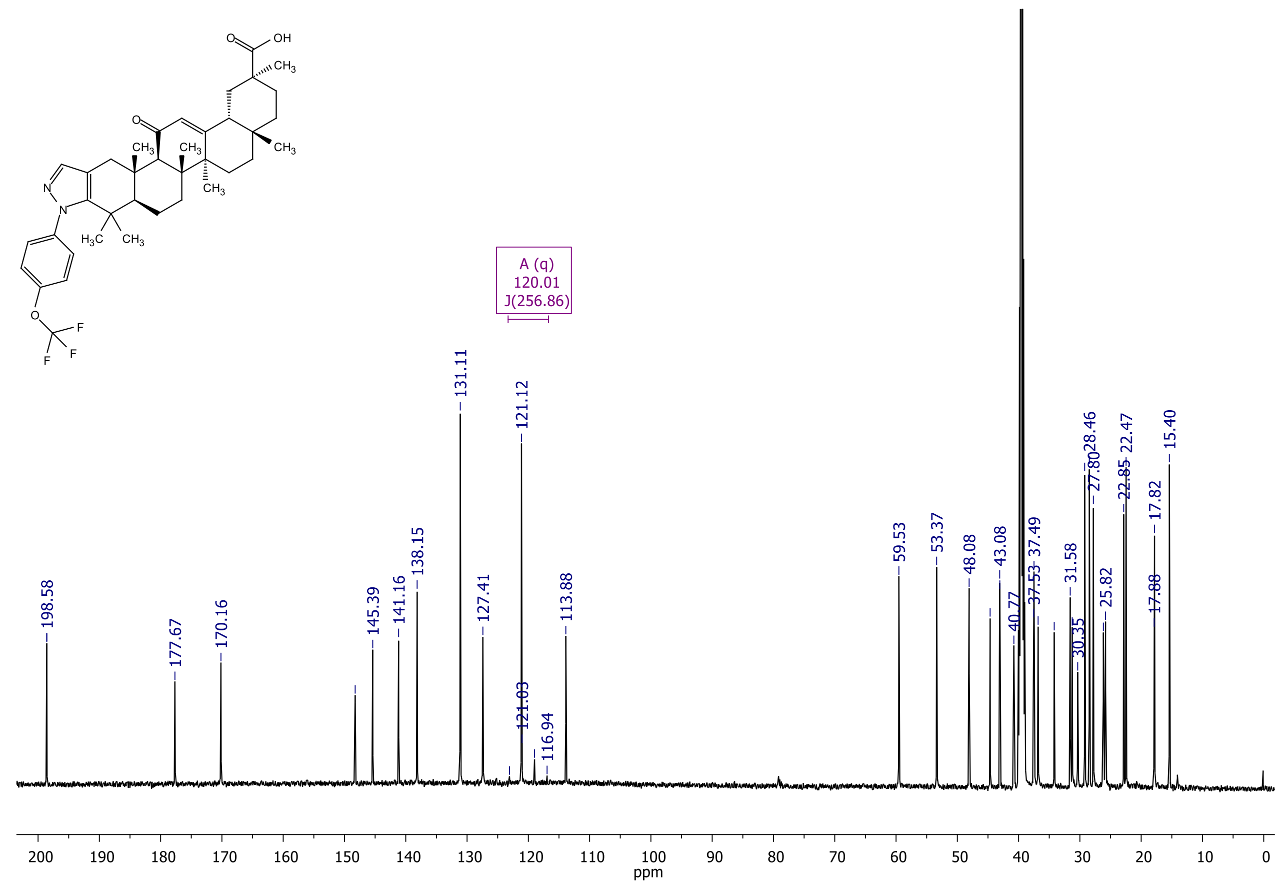

Figure S16. ^{13}C NMR (151 MHz) of compound 5a in DMSO-d_6

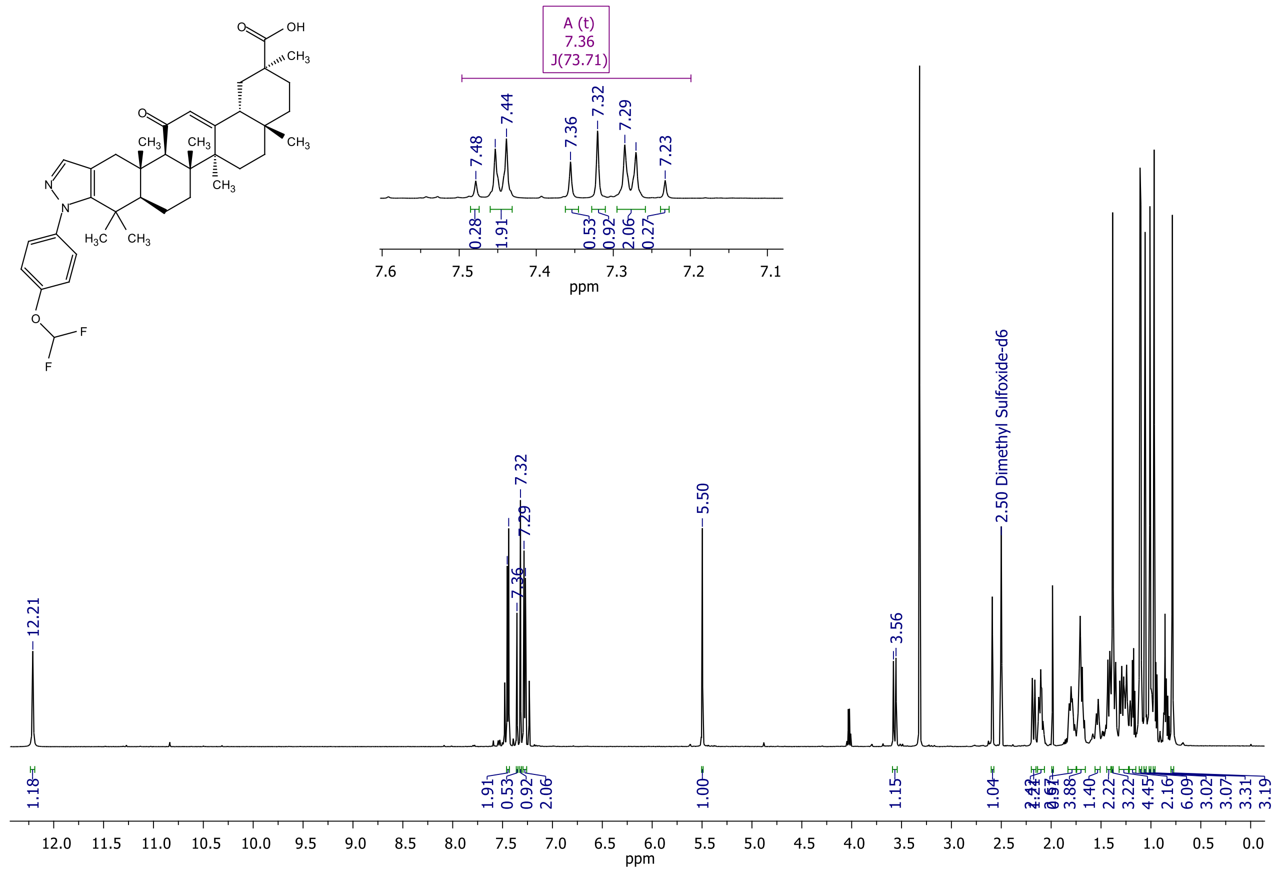

Figure S17. ^{1}H NMR (600 MHz) of compound 5b in DMSO-d_6

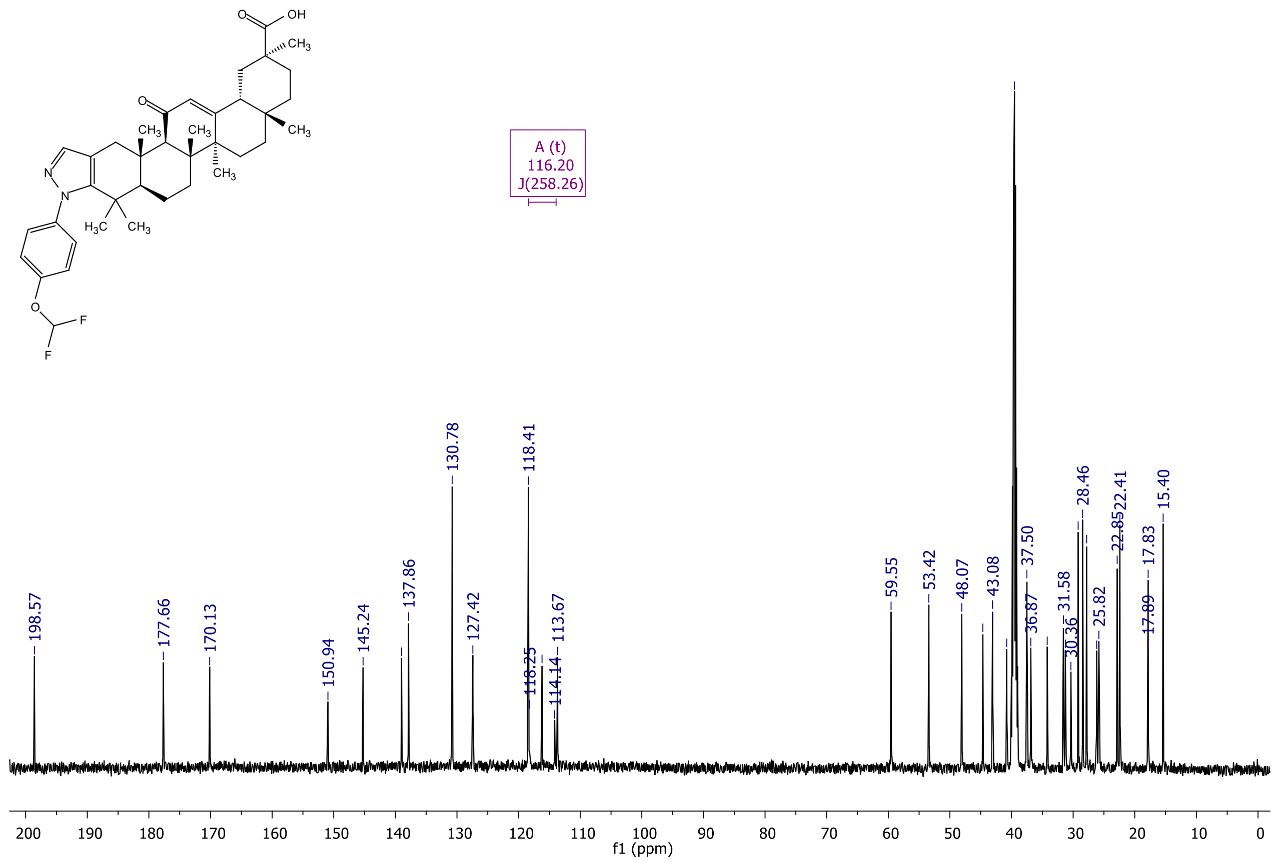

Figure S18. ^{13}C NMR (151 MHz) of compound 5b in DMSO-d_6

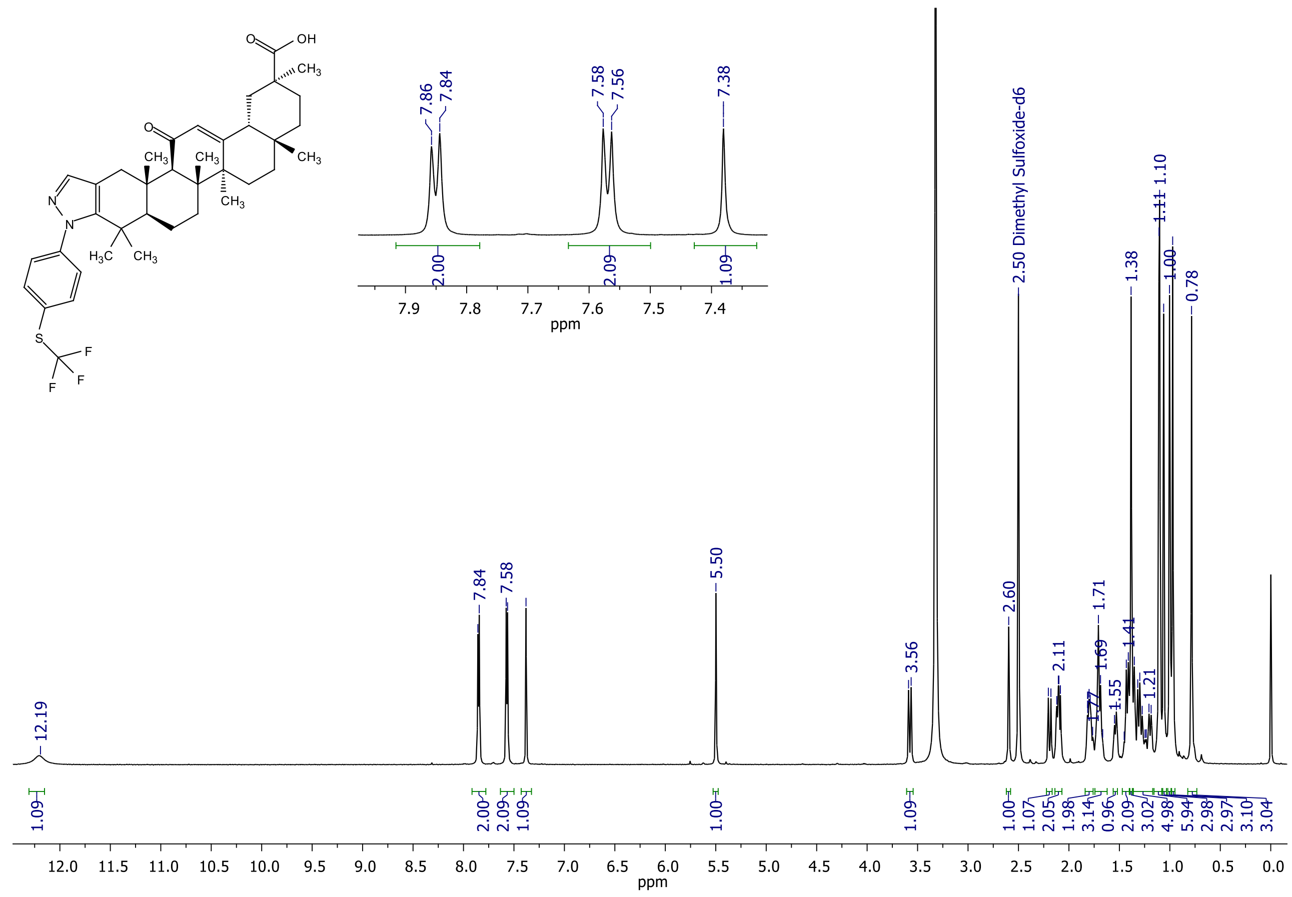

Figure S19. ^{1}H NMR (600 MHz) of compound 5c in DMSO-d_6

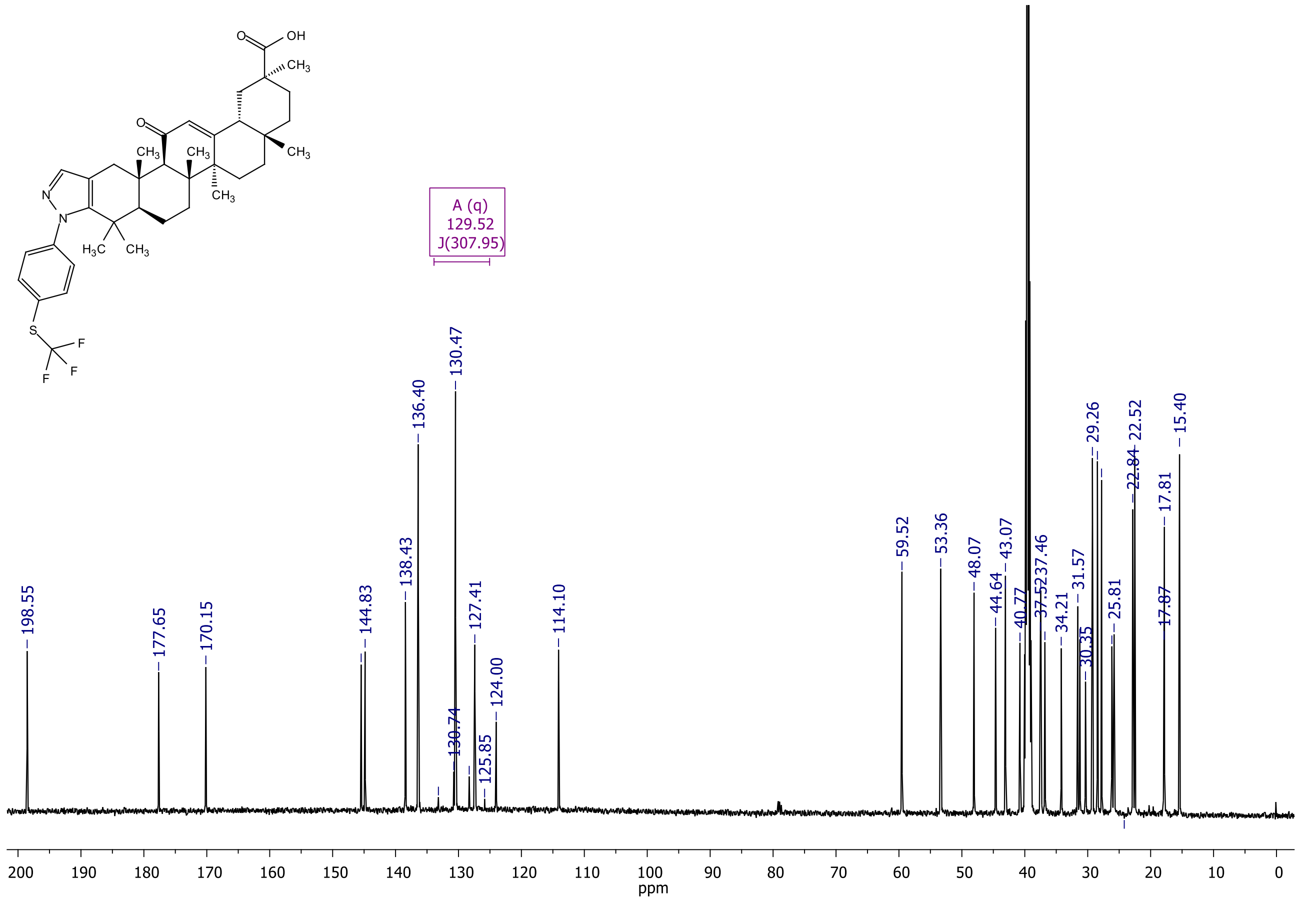

Figure S20. ^{13}C NMR (151 MHz) of compound 5c in DMSO-d_6

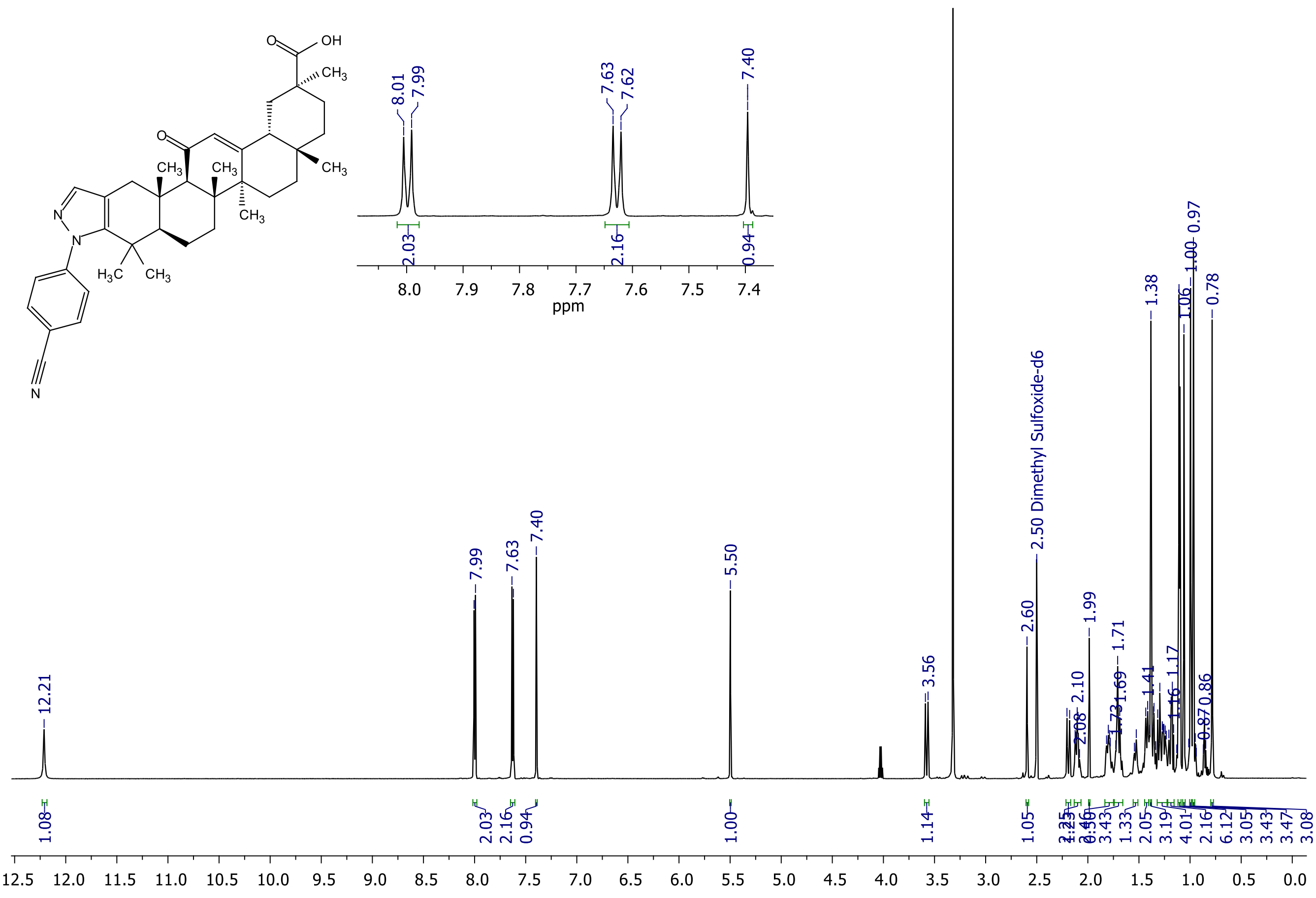

Figure S21. ^{1}H NMR (600 MHz) of compound 5d in DMSO-d_6

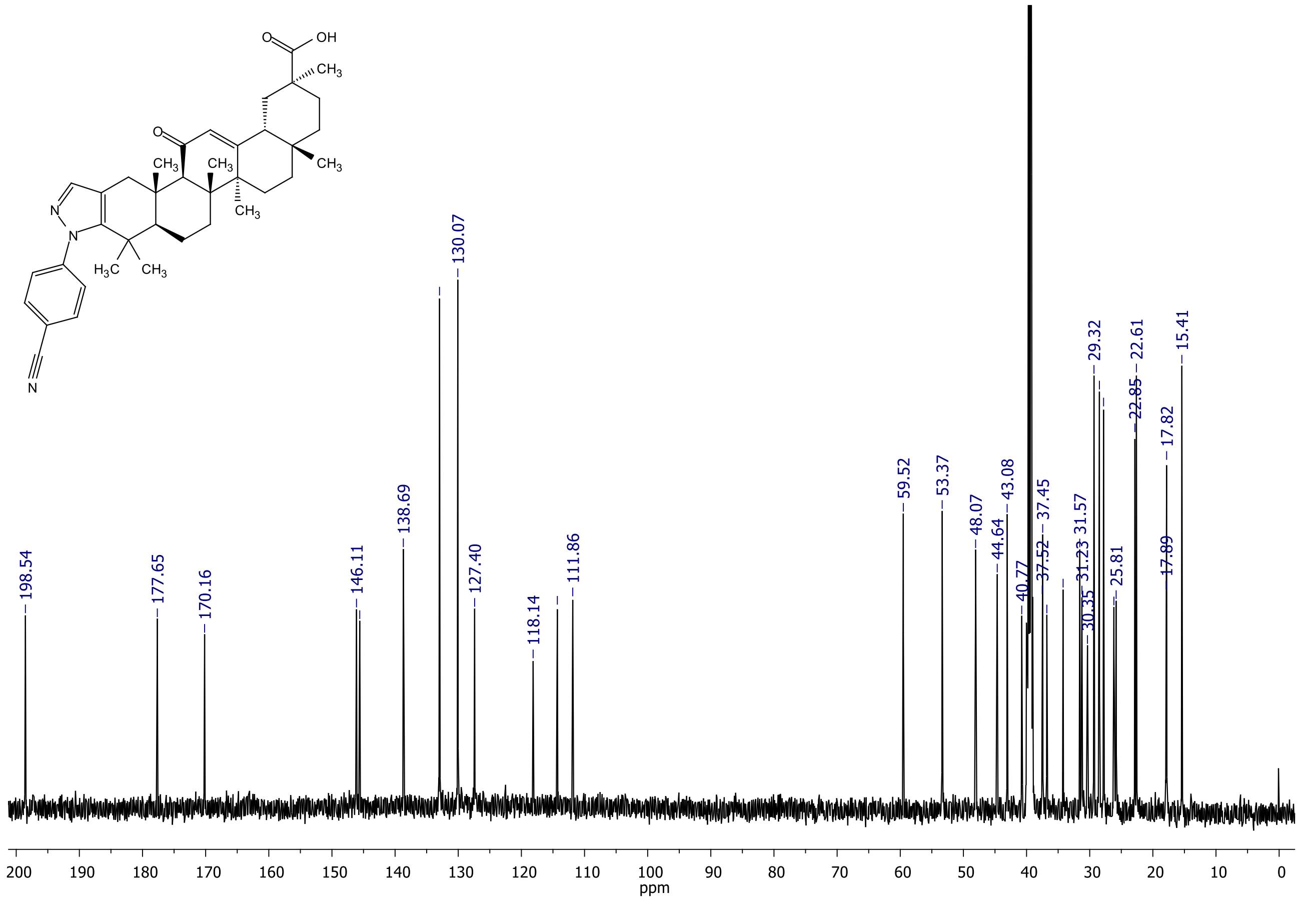

Figure S22. ^{13}C NMR (151 MHz) of compound 5d in DMSO-d_6

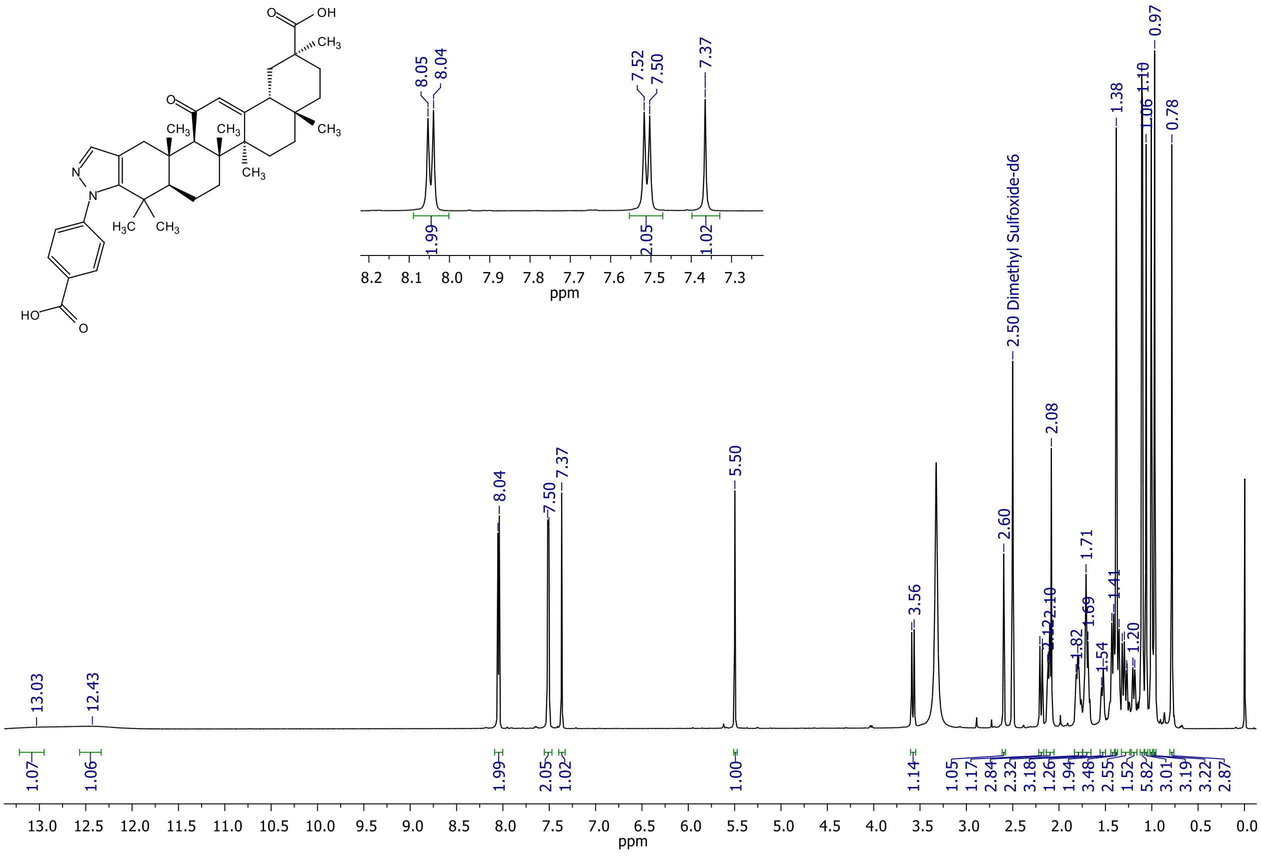

Figure S23. ^{1}H NMR (600 MHz) of compound 5e in DMSO-d_6

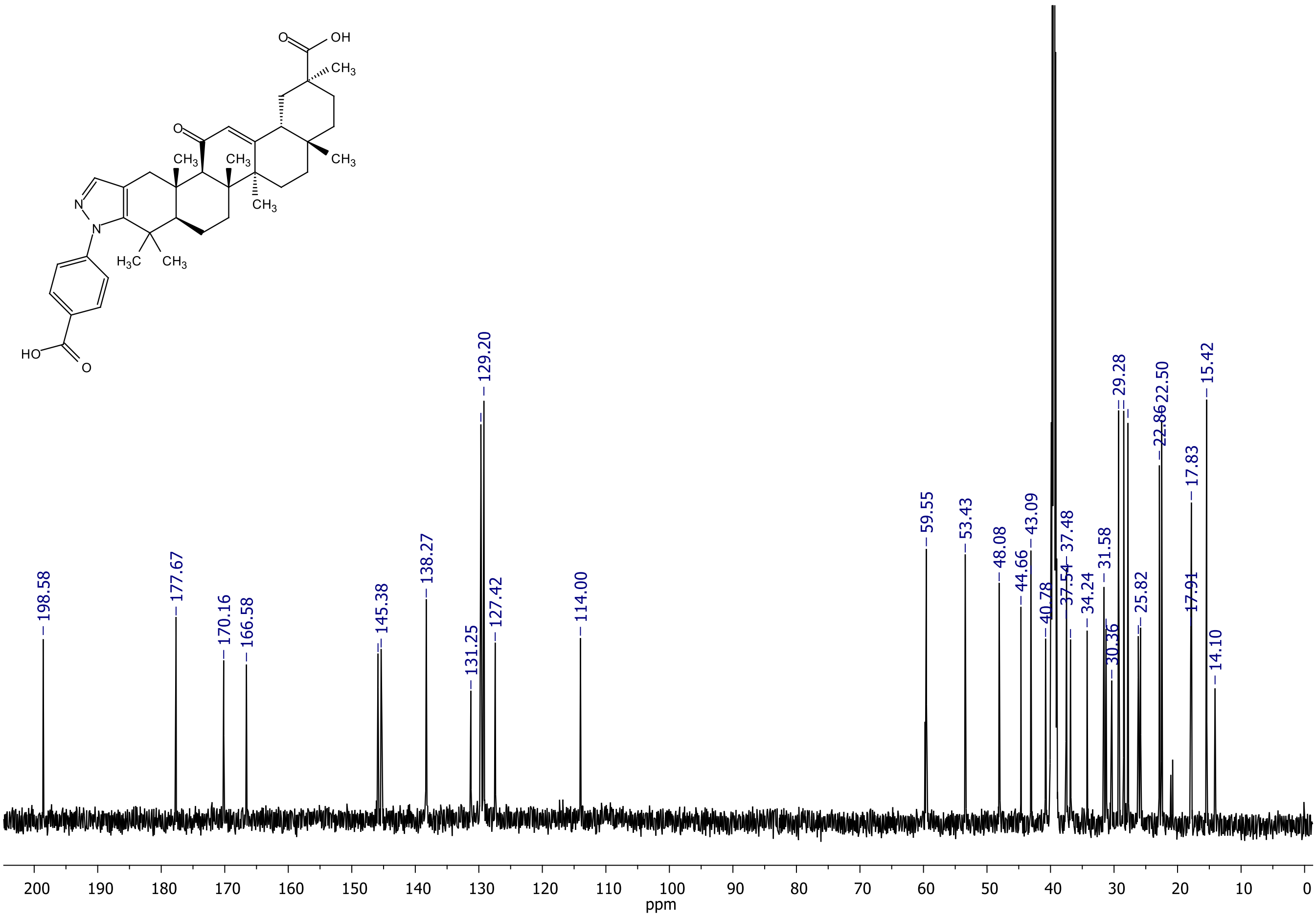

Figure S24. ^{13}C NMR (151 MHz) of compound 5e in DMSO-d_6

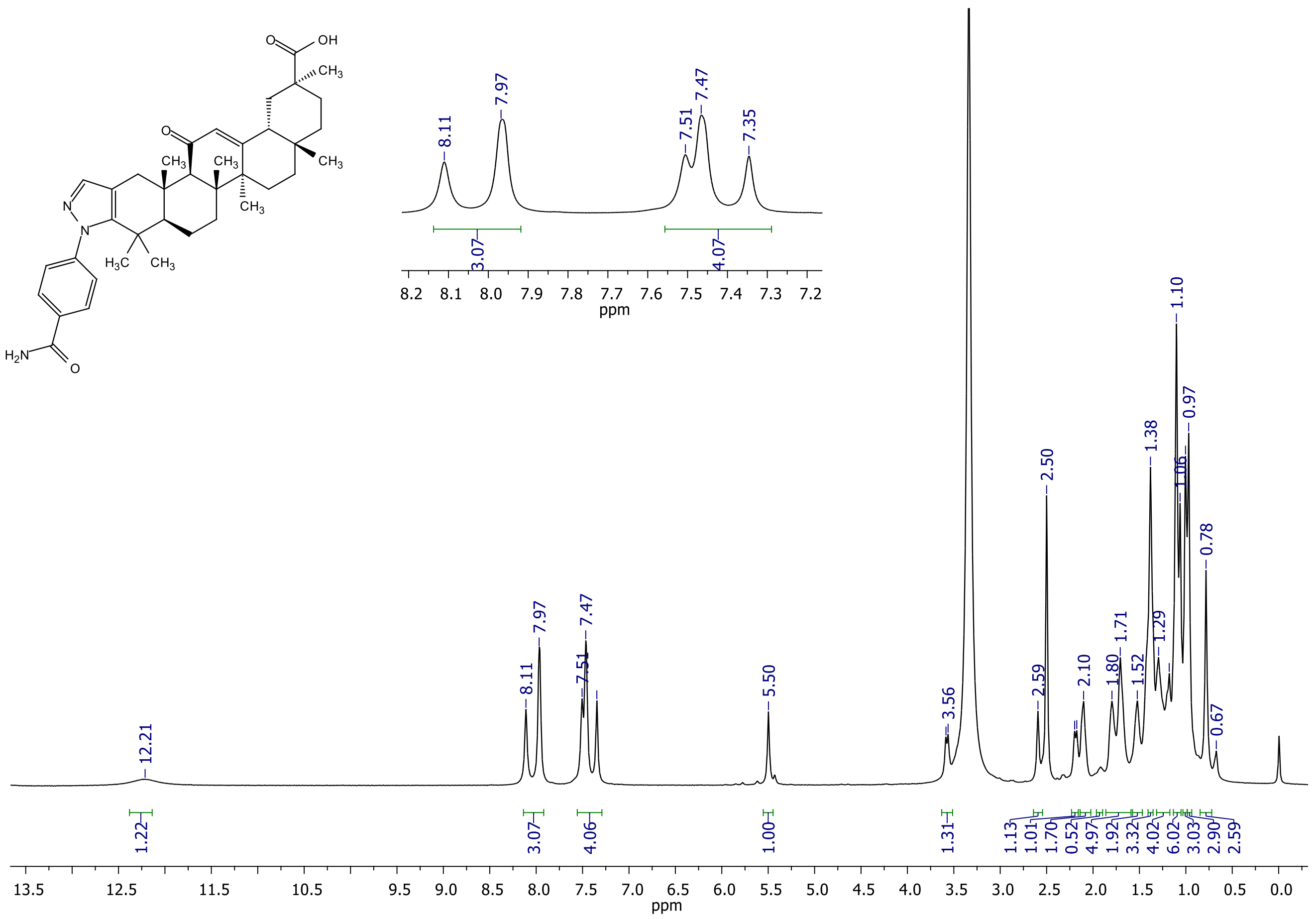

Figure S25. ^{1}H NMR (600 MHz) of compound 5f in DMSO-d_6

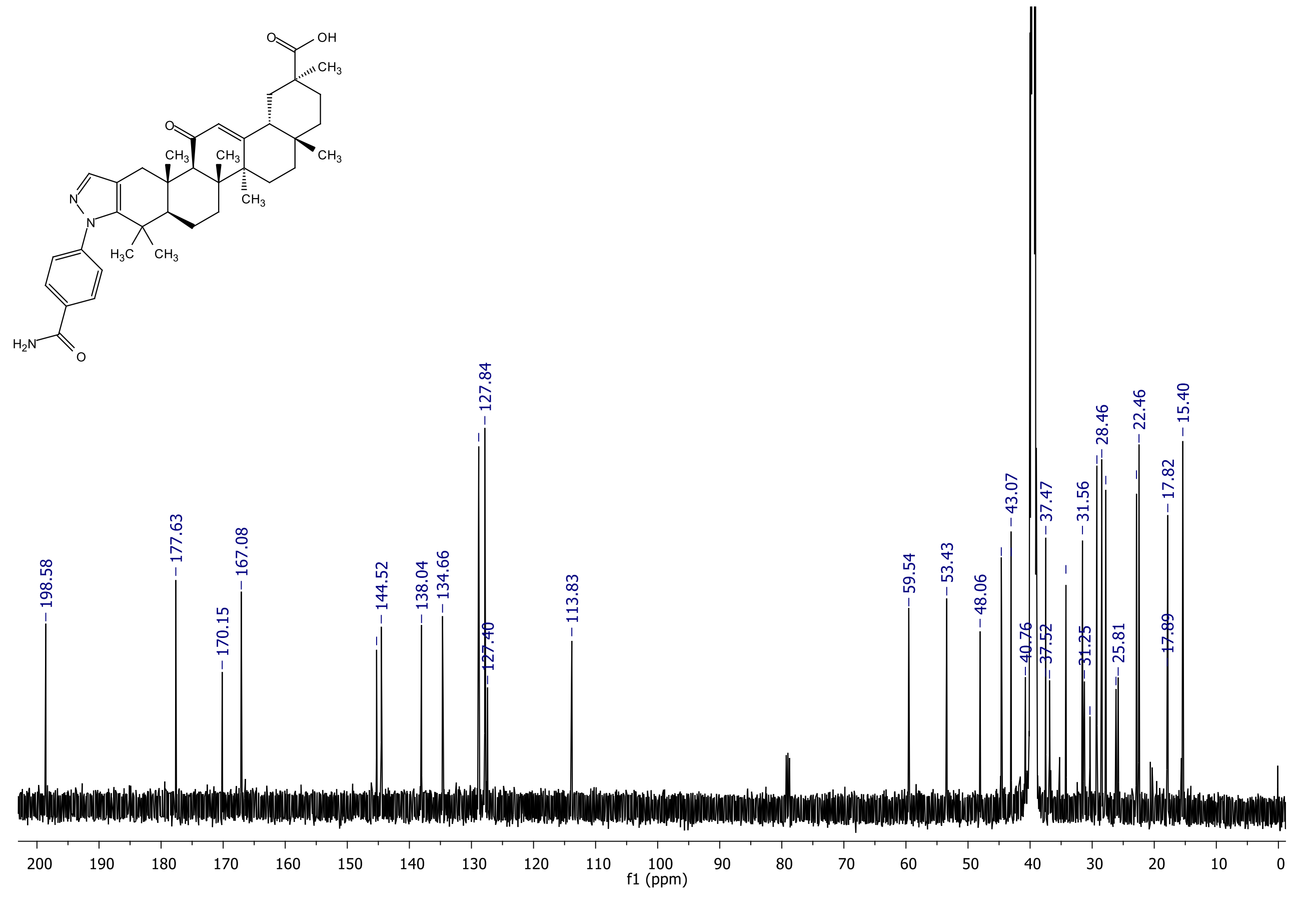

Figure S26. ^{13}C NMR (151 MHz) of compound 5f in DMSO-d_6

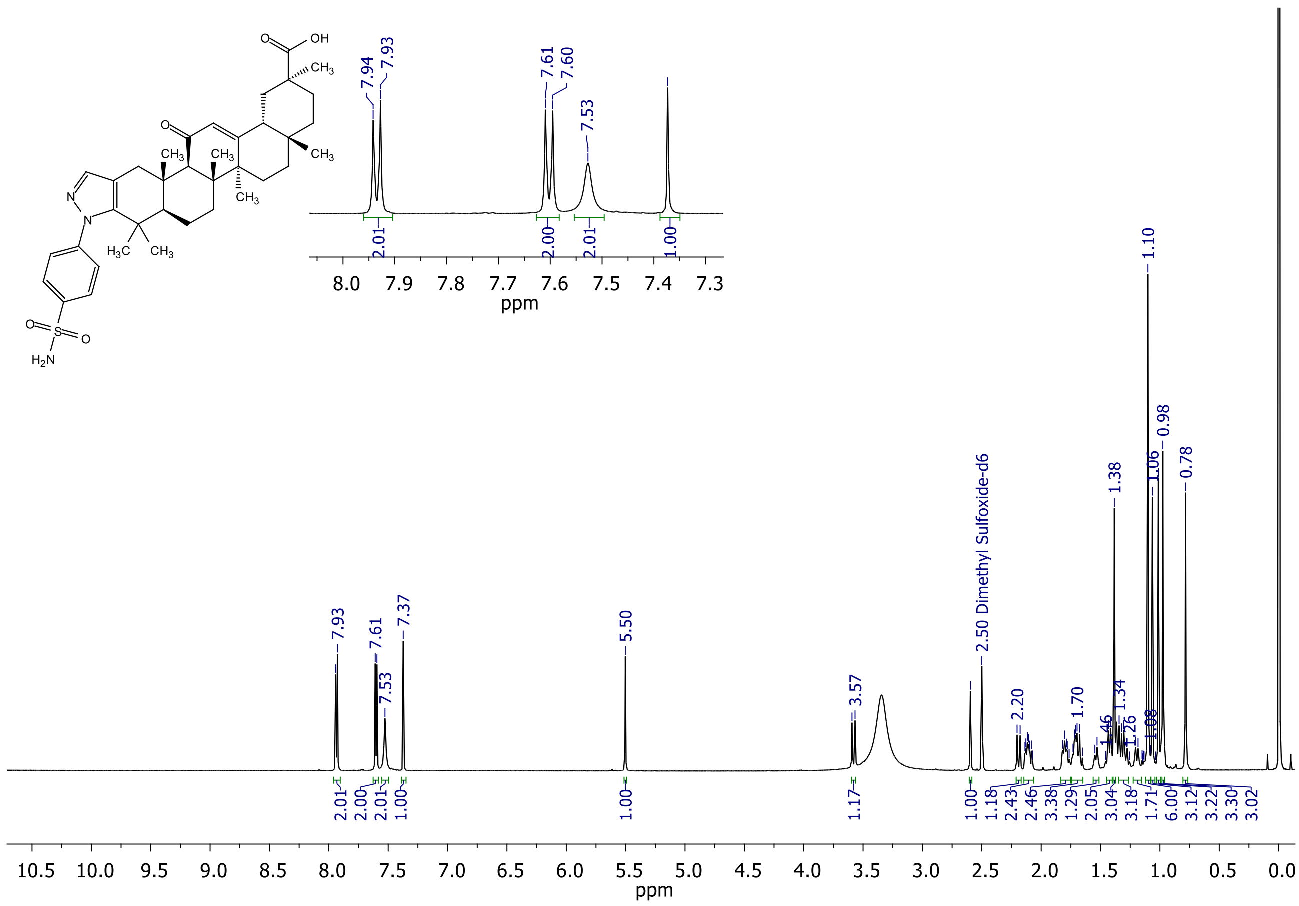

Figure S27. ^{1}H NMR (600 MHz) of compound 5g in DMSO-d_6

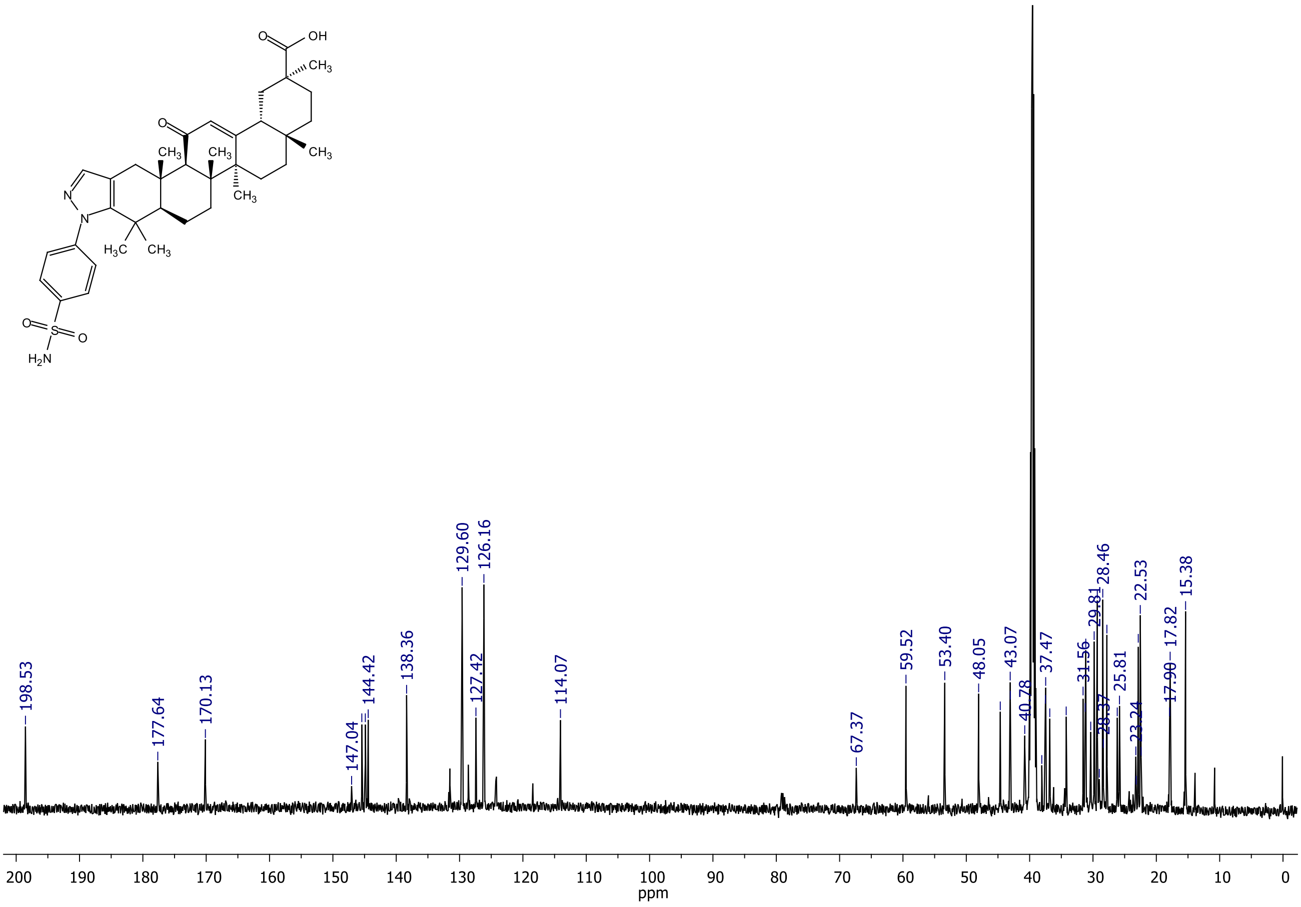

Figure S28. ^{13}C NMR (151 MHz) of compound 5g in DMSO-d_6

Mass Spectrum SmartFormula Report

Analysis Info

		Acquisition Date	8/21/2024 6:12:41 AM
Analysis Name	D:\Data\Monica Rincon\Francisco Cortes Benitez\20240819_C-72_r2.d		
Method	Tune Low extendido.m	Operator	Admin
Sample Name	20240819_C-72_r2	Instrument	micrOTOF 213750.00410
Comment			

Acquisition Parameter

Source Type	ESI	Ion Polarity	Positive	Set Nebulizer	0.5 Bar
Focus	Active			Set Dry Heater	150 °C
Scan Begin	50 m/z	Set Capillary	4500 V	Set Dry Gas	4.0 l/min
Scan End	3000 m/z	Set End Plate Offset	-500 V	Set Divert Valve	Waste

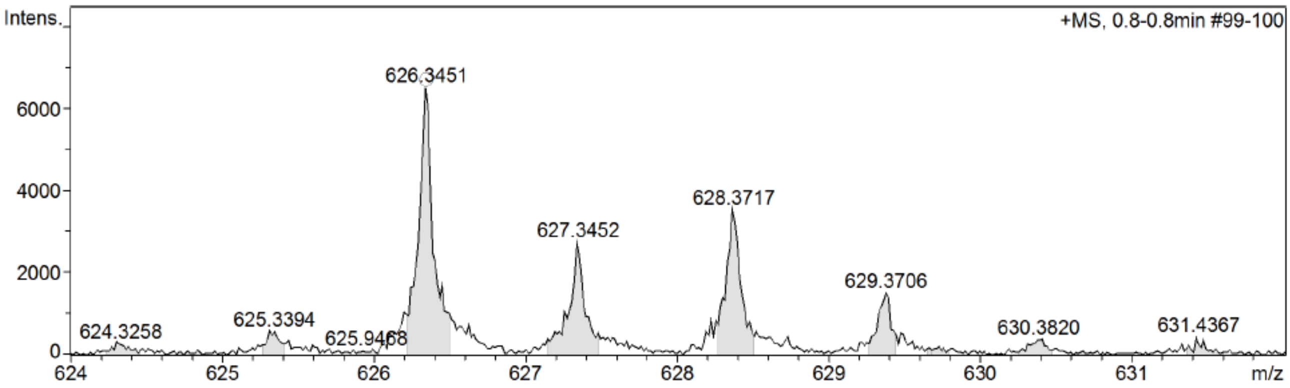

Meas. m/z	#	Ion Formula	Sum Formula	m/z	err [ppm]	|err| [mDa]	z	mSigma	# mSigma	e⁻ Conf
626.3451	1	C37H47F3NO4	C37H46F3NO4	626.3452	0.1	0.1	1+	46.1	1	even
648.3294	1	C37H46F3NNaO4		648.3271	-3.5	2.3	1+	60.8	1	even

Figure S29. Mass spectrum formula report of compound 4a.

Mass Spectrum SmartFormula Report

Analysis Info

		Acquisition Date	8/21/2024 6:01:14 AM
Analysis Name	D:\Data\Monica Rincon\Francisco Cortes Benitez\20240819_C-73_.d		
Method	Tune Low extendido.m	Operator	Admin
Sample Name	20240819_C-73_	Instrument	micrOTOF 213750.00410
Comment			

Acquisition Parameter

Source Type	ESI	Ion Polarity	Positive	Set Nebulizer	0.5 Bar
Focus	Active			Set Dry Heater	150 °C
Scan Begin	50 m/z	Set Capillary	4500 V	Set Dry Gas	4.0 l/min
Scan End	3000 m/z	Set End Plate Offset	-500 V	Set Divert Valve	Waste

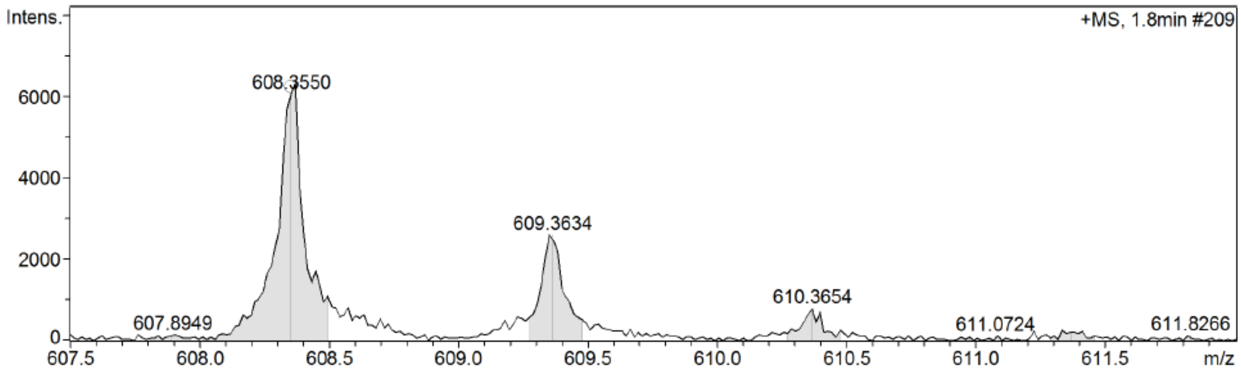

Meas. m/z	#	Ion Formula	Sum Formula	m/z	err [ppm]	|err| [mDa]	z	mSigma	# mSigma	e⁻ Conf
608.3550	1	C37H48F2NO4	C37H47F2NO4	608.3546	-0.7	0.4	1+	24.7	2	even

Figure S30. Mass spectrum formula report of compound 4b.

Mass Spectrum SmartFormula Report

Analysis Info

		Acquisition Date	8/22/2024 1:35:46 AM
Analysis Name	D:\Data\Monica Rincon\Francisco Cortes Benitez\20240820_C-74.d		
Method	Tune Low extendido.m	Operator	Admin
Sample Name	20240820_C-74	Instrument	micrOTOF 213750.00410
Comment			

Acquisition Parameter

Source Type	ESI	Ion Polarity	Positive	Set Nebulizer	0.5 Bar
Focus	Active			Set Dry Heater	150 °C
Scan Begin	50 m/z	Set Capillary	4500 V	Set Dry Gas	4.0 l/min
Scan End	3000 m/z	Set End Plate Offset	-500 V	Set Divert Valve	Waste

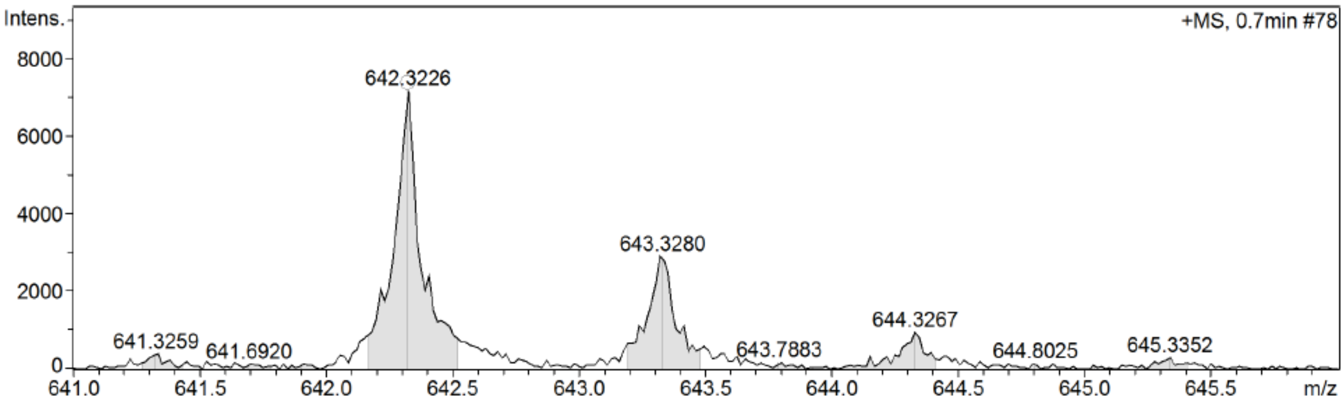

Meas. m/z	#	Ion Formula	Sum Formula	m/z	err [ppm]	|err| [mDa]	z	mSigma	# mSigma	e⁻ Conf
642.3226	1	C37H47F3NO3S	C37H46F3NO3S	642.3223	-0.5	0.3	1+	7.3	3	even

Figure S31. Mass spectrum formula report of compound 4c.

Mass Spectrum SmartFormula Report

Analysis Info

		Acquisition Date	8/22/2024 1:54:39 AM
Analysis Name	D:\Data\Monica Rincon\Francisco Cortes Benitez\20240820_C-75.d		
Method	Tune Low extendido.m	Operator	Admin
Sample Name	20240820_C-75	Instrument	micrOTOF 213750.00410
Comment			

Acquisition Parameter

Source Type	ESI	Ion Polarity	Positive	Set Nebulizer	0.5 Bar
Focus	Active			Set Dry Heater	150 °C
Scan Begin	50 m/z	Set Capillary	4500 V	Set Dry Gas	4.0 l/min
Scan End	3000 m/z	Set End Plate Offset	-500 V	Set Divert Valve	Waste

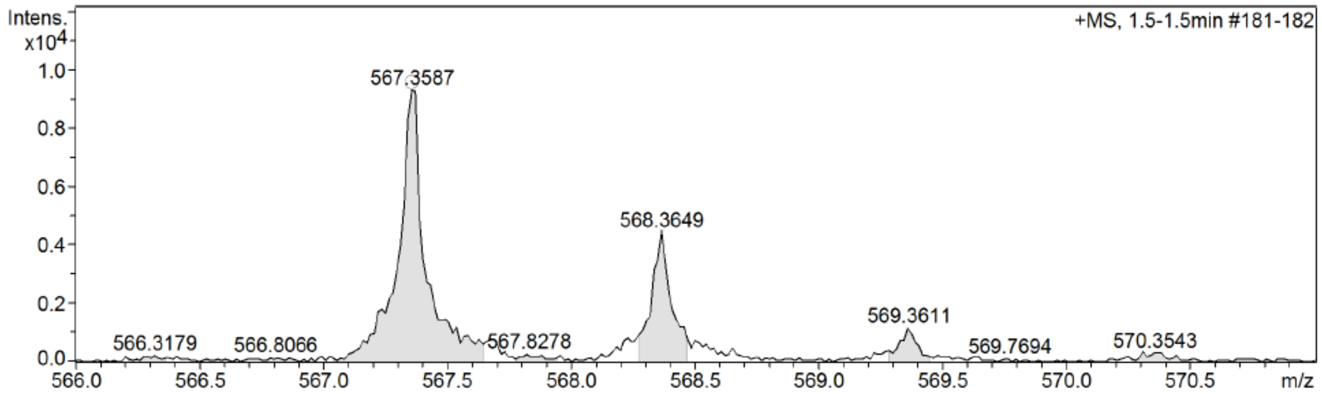

Meas. m/z	#	Ion Formula	Sum Formula	m/z	err [ppm]	|err| [mDa]	z	mSigma	# mSigma	e⁻ Conf
567.3587	1	C37H47N2O3	C37H46N2O3	567.3581	-1.0	0.6	1+	36.4	1	even
589.3388	1	C37H46N2NaO3		589.3401	2.2	1.3	1+	227.2	2	even

Figure S32. Mass spectrum formula report of compound 4d.

Mass Spectrum SmartFormula Report

Analysis Info

		Acquisition Date	8/22/2024 3:12:35 AM
Analysis Name	D:\Data\Monica Rincon\Francisco Cortes Benitez\20240820_C-76_.d		
Method	Tune Low extendido.m	Operator	Admin
Sample Name	20240820_C-76_	Instrument	micrOTOF 213750.00410
Comment			

Acquisition Parameter

Source Type	ESI	Ion Polarity	Positive	Set Nebulizer	0.5 Bar
Focus	Active			Set Dry Heater	150 °C
Scan Begin	50 m/z	Set Capillary	4500 V	Set Dry Gas	4.0 l/min
Scan End	3000 m/z	Set End Plate Offset	-500 V	Set Divert Valve	Waste

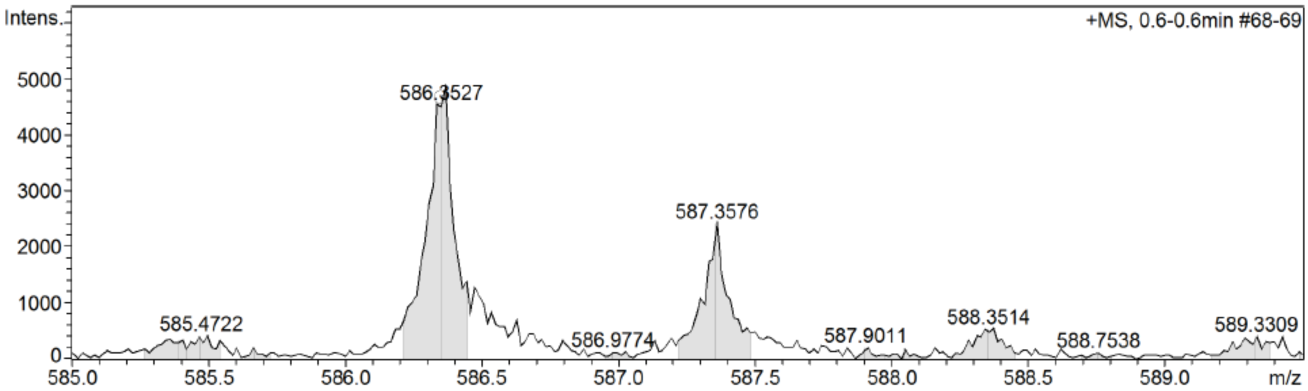

Meas. m/z	#	Ion Formula	Sum Formula	m/z	err [ppm]	|err| [mDa]	z	mSigma	# mSigma	e¯ Conf
586.3527	1	C37H48NO5	C37H47NO5	586.3527	-0.0	0.0	1+	62.4	1	even
608.3358	1	C37H47NNaO5		608.3346	-1.9	1.2	1+	58.5	1	even
624.3131	1	C37H47KNO5		624.3086	-7.2	4.5	1+	21.6	1	even

Figure S33. Mass spectrum formula report of compound 4e.

Mass Spectrum SmartFormula Report

Analysis Info

		Acquisition Date	8/22/2024 2:11:50 AM
Analysis Name	D:\Data\Monica Rincon\Francisco Cortes Benitez\20240820_C-77.d		
Method	Tune Low extendido.m	Operator	Admin
Sample Name	20240820_C-77	Instrument	micrOTOF 213750.00410
Comment			

Acquisition Parameter

Source Type	ESI	Ion Polarity	Positive	Set Nebulizer	0.5 Bar
Focus	Active			Set Dry Heater	150 °C
Scan Begin	50 m/z	Set Capillary	4500 V	Set Dry Gas	4.0 l/min
Scan End	3000 m/z	Set End Plate Offset	-500 V	Set Divert Valve	Waste

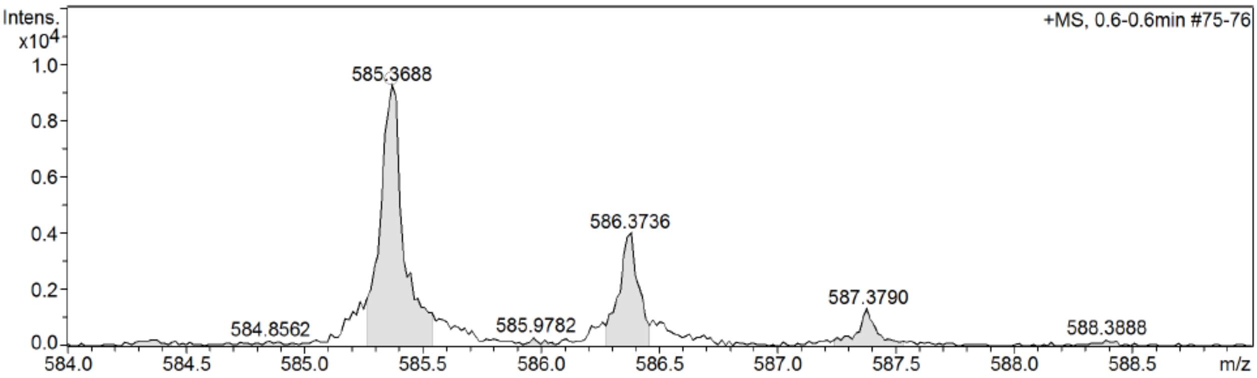

Meas. m/z	#	Ion Formula	Sum Formula	m/z	err [ppm]	|err| [mDa]	z	mSigma	# mSigma	e¯ Conf
585.3688	1	C37H49N2O4	C37H48N2O4	585.3687	-0.2	0.1	1+	28.0	1	even
607.3511	1	C37H48N2NaO4		607.3506	-0.8	0.5	1+	100.2	1	even
623.3289	1	C37H48KN2O4		623.3246	-6.9	4.3	1+	78.7	4	even

Figure S34. Mass spectrum formula report of compound 4f.

Mass Spectrum SmartFormula Report

Analysis Info

Analysis Name	D:\Data\Monica Rincon\Francisco Cortes Benitez\20240820_C-78.d	Acquisition Date	8/22/2024 3:30:02 AM
Method	Tune Low extendido.m	Operator	Admin
Sample Name	20240820_C-78	Instrument	micrOTOF 213750.00410
Comment			

Acquisition Parameter

Source Type	ESI	Ion Polarity	Positive	Set Nebulizer	0.5 Bar
Focus	Active			Set Dry Heater	150 °C
Scan Begin	50 m/z	Set Capillary	4500 V	Set Dry Gas	4.0 l/min
Scan End	3000 m/z	Set End Plate Offset	-500 V	Set Divert Valve	Waste

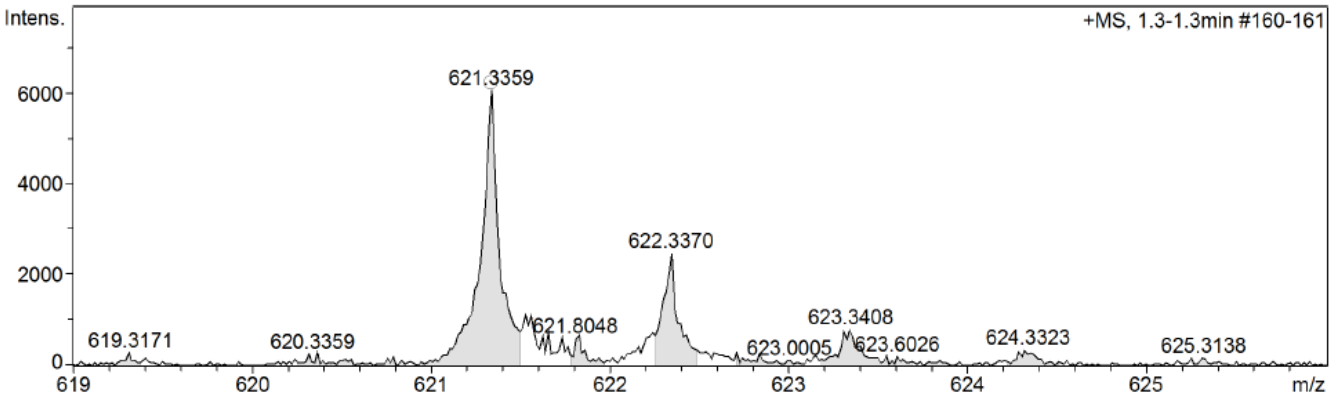

Meas. m/z	#	Ion Formula	Sum Formula	m/z	err [ppm]	|err| [mDa]	z	mSigma	# mSigma	e⁻ Conf
621.3359	1	C36H49N2O5S	C36H48N2O5S	621.3357	-0.4	0.2	1+	12.7	1	even
643.3166	1	C36H48N2NaO5S		643.3176	1.5	1.0	1+	31.1	2	even

Figure S35. Mass spectrum formula report of compound 4g.

Mass Spectrum SmartFormula Report

Analysis Info

Analysis Name	D:\Data\Monica Rincon\Francisco Cortes Benitez\20240819_C-71.d	Acquisition Date	8/21/2024 5:13:33 AM
Method	Tune Low extendido.m	Operator	Admin
Sample Name	20240819_C-71	Instrument	micrOTOF 213750.00410
Comment			

Acquisition Parameter

Source Type	ESI	Ion Polarity	Positive	Set Nebulizer	0.5 Bar
Focus	Active			Set Dry Heater	150 °C
Scan Begin	50 m/z	Set Capillary	4500 V	Set Dry Gas	4.0 l/min
Scan End	3000 m/z	Set End Plate Offset	-500 V	Set Divert Valve	Waste

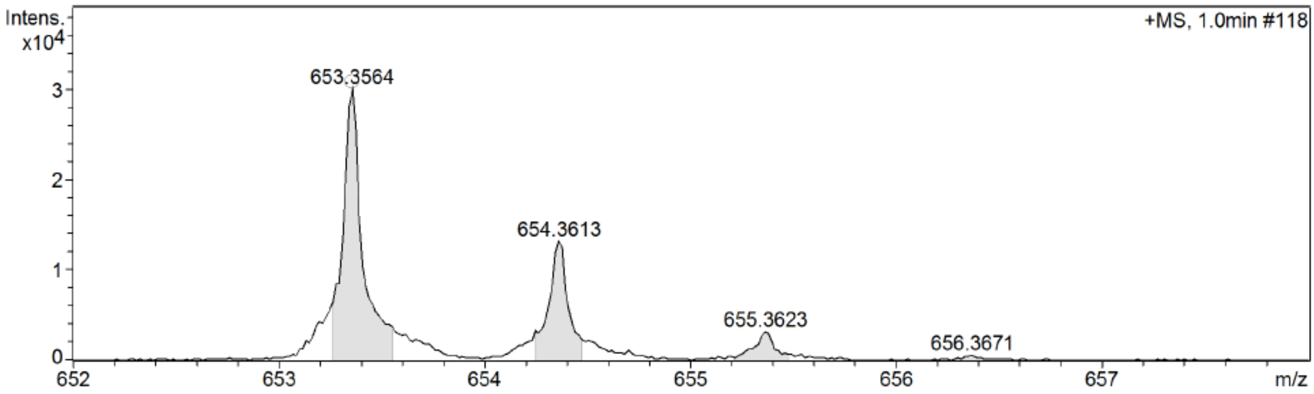

Meas. m/z	#	Ion Formula	Sum Formula	m/z	err [ppm]	|err| [mDa]	z	mSigma	# mSigma	e⁻ Conf
653.3564	1	C38H48F3N2O4	C38H47F3N2O4	653.3561	-0.5	0.3	1+	8.5	1	even

Figure S36. Mass spectrum formula report of compound 5a.

Mass Spectrum SmartFormula Report

Analysis Info

		Acquisition Date	8/21/2024 4:55:55 AM
Analysis Name	D:\Data\Monica Rincon\Francisco Cortes Benitez\20240819_C-70.d		
Method	Tune Low extendido.m	Operator	Admin
Sample Name	20240819_C-70	Instrument	micrOTOF 213750.00410
Comment			

Acquisition Parameter

Source Type	ESI	Ion Polarity	Positive	Set Nebulizer	0.5 Bar
Focus	Active			Set Dry Heater	150 °C
Scan Begin	50 m/z	Set Capillary	4500 V	Set Dry Gas	4.0 l/min
Scan End	3000 m/z	Set End Plate Offset	-500 V	Set Divert Valve	Waste

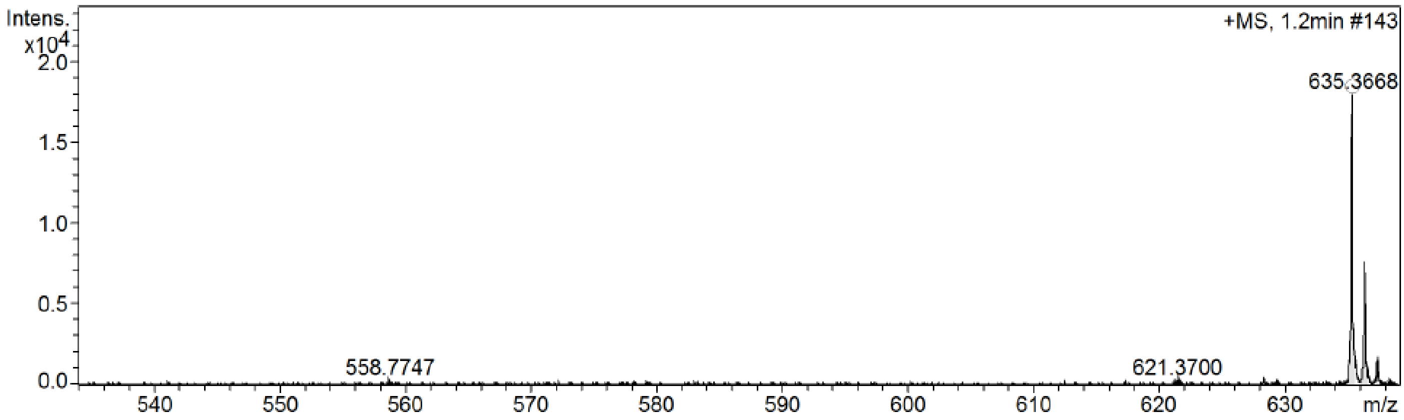

Meas. m/z	#	Ion Formula	Sum Formula	m/z	err [ppm]	|err| [mDa]	z	mSigma	# mSigma	e⁻ Conf
635.3668	1	C38H49F2N2O4	C38H48F2N2O4	635.3655	-2.0	1.3	1+	8.5	1	even

Figure S37. Mass spectrum formula report of compound 5b.

Mass Spectrum SmartFormula Report

Analysis Info

		Acquisition Date	8/23/2024 4:33:48 AM
Analysis Name	D:\Data\Monica Rincon\Francisco Cortes Benitez\20240822_C-91.d		
Method	Tune Low extendido.m	Operator	Admin
Sample Name	20240822_C-91	Instrument	micrOTOF 213750.00410
Comment			

Acquisition Parameter

Source Type	ESI	Ion Polarity	Positive	Set Nebulizer	0.5 Bar
Focus	Active			Set Dry Heater	150 °C
Scan Begin	50 m/z	Set Capillary	4500 V	Set Dry Gas	4.0 l/min
Scan End	3000 m/z	Set End Plate Offset	-500 V	Set Divert Valve	Waste

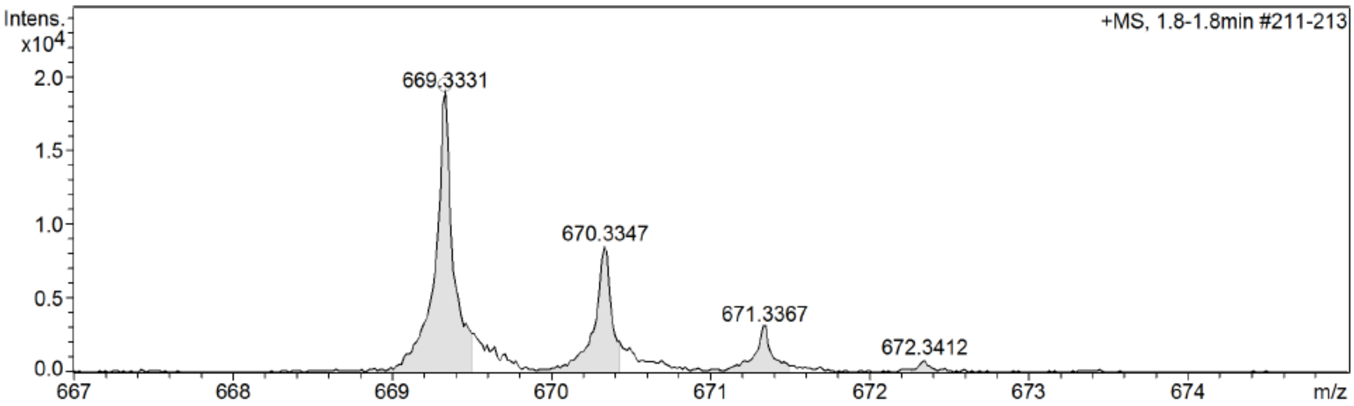

Meas. m/z	#	Ion Formula	Sum Formula	m/z	err [ppm]	|err| [mDa]	z	mSigma	# mSigma	e⁻ Conf
669.3331	1	C38H48F3N2O3S	C38H47F3N2O3S	669.3332	0.2	0.1	1+	16.4	1	even

Figure S38. Mass spectrum formula report of compound 5c.

Mass Spectrum SmartFormula Report

Analysis Info

Analysis Name	D:\Data\Monica Rincon\Francisco Cortes Benitez\20240820_C-86.d	Acquisition Date	8/22/2024 4:03:36 AM
Method	Tune Low extendido.m	Operator	Admin
Sample Name	20240820_C-86	Instrument	micrOTOF 213750.00410
Comment			

Acquisition Parameter

Source Type	ESI	Ion Polarity	Positive	Set Nebulizer	0.5 Bar
Focus	Active			Set Dry Heater	150 °C
Scan Begin	50 m/z	Set Capillary	4500 V	Set Dry Gas	4.0 l/min
Scan End	3000 m/z	Set End Plate Offset	-500 V	Set Divert Valve	Waste

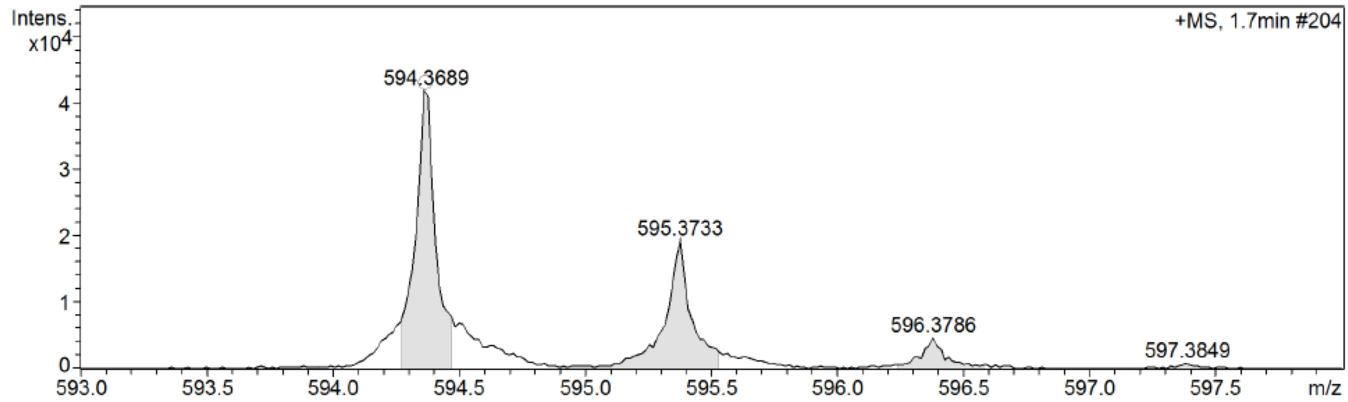

Meas. m/z	#	Ion Formula	Sum Formula	m/z	err [ppm]	|err| [mDa]	z	mSigma	# mSigma	e⁻ Conf
594.3689	1	C38H48N3O3	C38H47N3O3	594.3690	0.2	0.1	1+	18.3	1	even

Figure S39. Mass spectrum formula report of compound 5d.

Mass Spectrum SmartFormula Report

Analysis Info

Analysis Name	D:\Data\Monica Rincon\Francisco Cortes Benitez\20240822_C-89.d	Acquisition Date	8/23/2024 3:59:24 AM
Method	Tune Low extendido.m	Operator	Admin
Sample Name	20240822_C-89	Instrument	micrOTOF 213750.00410
Comment			

Acquisition Parameter

Source Type	ESI	Ion Polarity	Positive	Set Nebulizer	0.5 Bar
Focus	Active			Set Dry Heater	150 °C
Scan Begin	50 m/z	Set Capillary	4500 V	Set Dry Gas	4.0 l/min
Scan End	3000 m/z	Set End Plate Offset	-500 V	Set Divert Valve	Waste

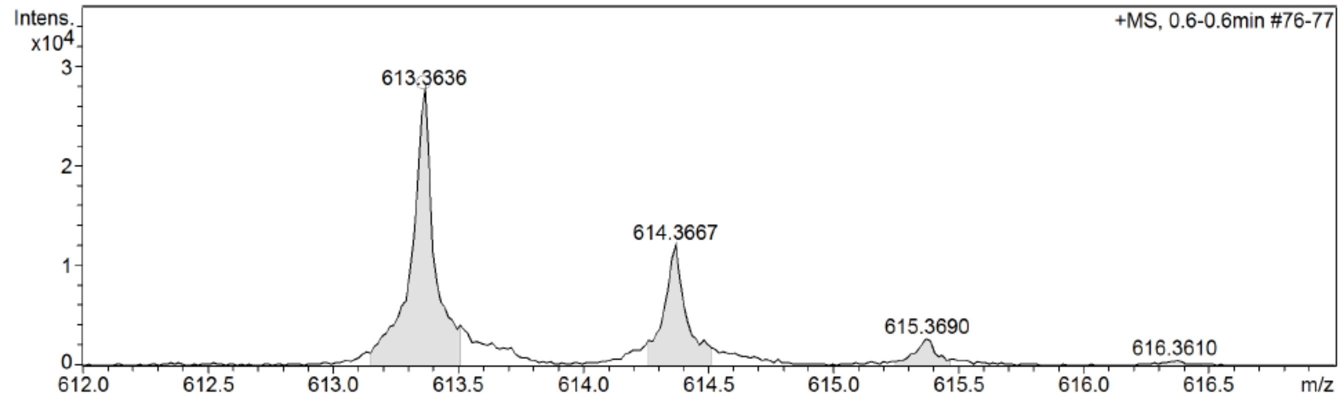

Meas. m/z	#	Ion Formula	Sum Formula	m/z	err [ppm]	|err| [mDa]	z	mSigma	# mSigma	e⁻ Conf
613.3636	1	C38H49N2O5	C38H48N2O5	613.3636	-0.1	0.0	1+	10.4	1	even

Figure S40. Mass spectrum formula report of compound 5e.

Mass Spectrum SmartFormula Report

Analysis Info

		Acquisition Date	8/22/2024 4:16:29 AM
Analysis Name	D:\Data\Monica Rincon\Francisco Cortes Benitez\20240820_C-88.d		
Method	Tune Low extendido.m	Operator	Admin
Sample Name	20240820_C-88	Instrument	micrOTOF 213750.00410
Comment			

Acquisition Parameter

Source Type	ESI	Ion Polarity	Positive	Set Nebulizer	0.5 Bar
Focus	Active			Set Dry Heater	150 °C
Scan Begin	50 m/z	Set Capillary	4500 V	Set Dry Gas	4.0 l/min
Scan End	3000 m/z	Set End Plate Offset	-500 V	Set Divert Valve	Waste

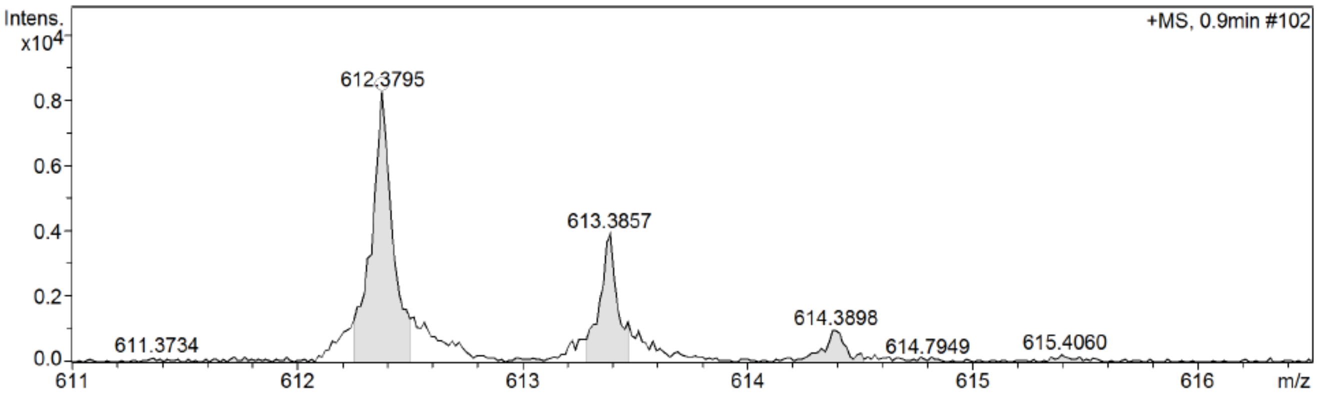

Meas. m/z	#	Ion Formula	Sum Formula	m/z	err [ppm]	|err| [mDa]	z	mSigma	# mSigma	e⁻ Conf
612.3795	1	C38H50N3O4	C38H49N3O4	612.3796	0.2	0.1	1+	26.5	1	even

Figure S41. Mass spectrum formula report of compound 5f.

Mass Spectrum SmartFormula Report

Analysis Info

		Acquisition Date	8/22/2024 3:46:28 AM
Analysis Name	D:\Data\Monica Rincon\Francisco Cortes Benitez\20240820_C-84.d		
Method	Tune Low extendido.m	Operator	Admin
Sample Name	20240820_C-84	Instrument	micrOTOF 213750.00410
Comment			

Acquisition Parameter

Source Type	ESI	Ion Polarity	Positive	Set Nebulizer	0.5 Bar
Focus	Active			Set Dry Heater	150 °C
Scan Begin	50 m/z	Set Capillary	4500 V	Set Dry Gas	4.0 l/min
Scan End	3000 m/z	Set End Plate Offset	-500 V	Set Divert Valve	Waste

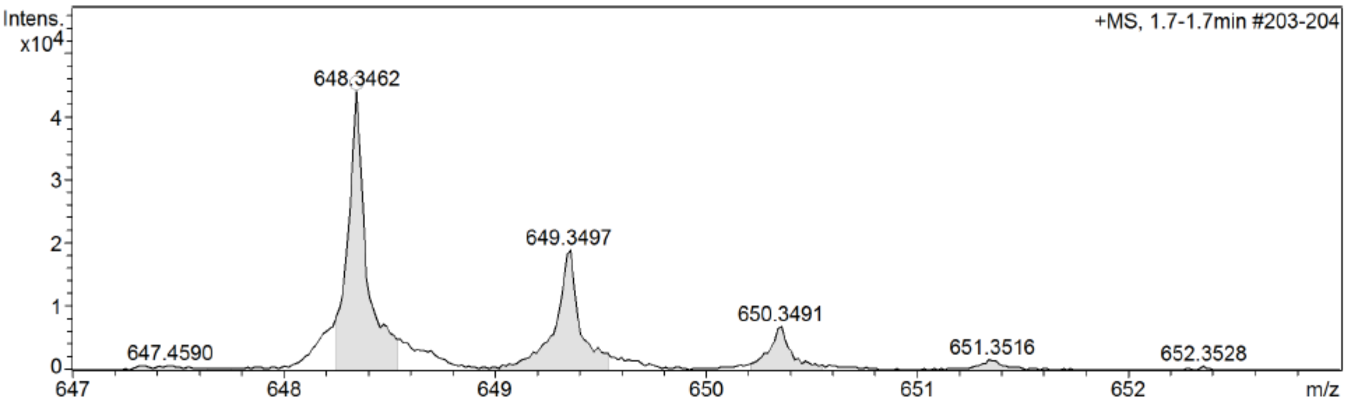

Meas. m/z	#	Ion Formula	Sum Formula	m/z	err [ppm]	|err| [mDa]	z	mSigma	# mSigma	e⁻ Conf
648.3462	1	C37H50N3O5S	C37H49N3O5S	648.3466	0.5	0.3	1+	7.9	2	even
1295.6805	1	C74H99N6O10S2		1295.6859	4.1	5.4	1+	89.0	5	even

Figure S42. Mass spectrum formula report of compound 5g.

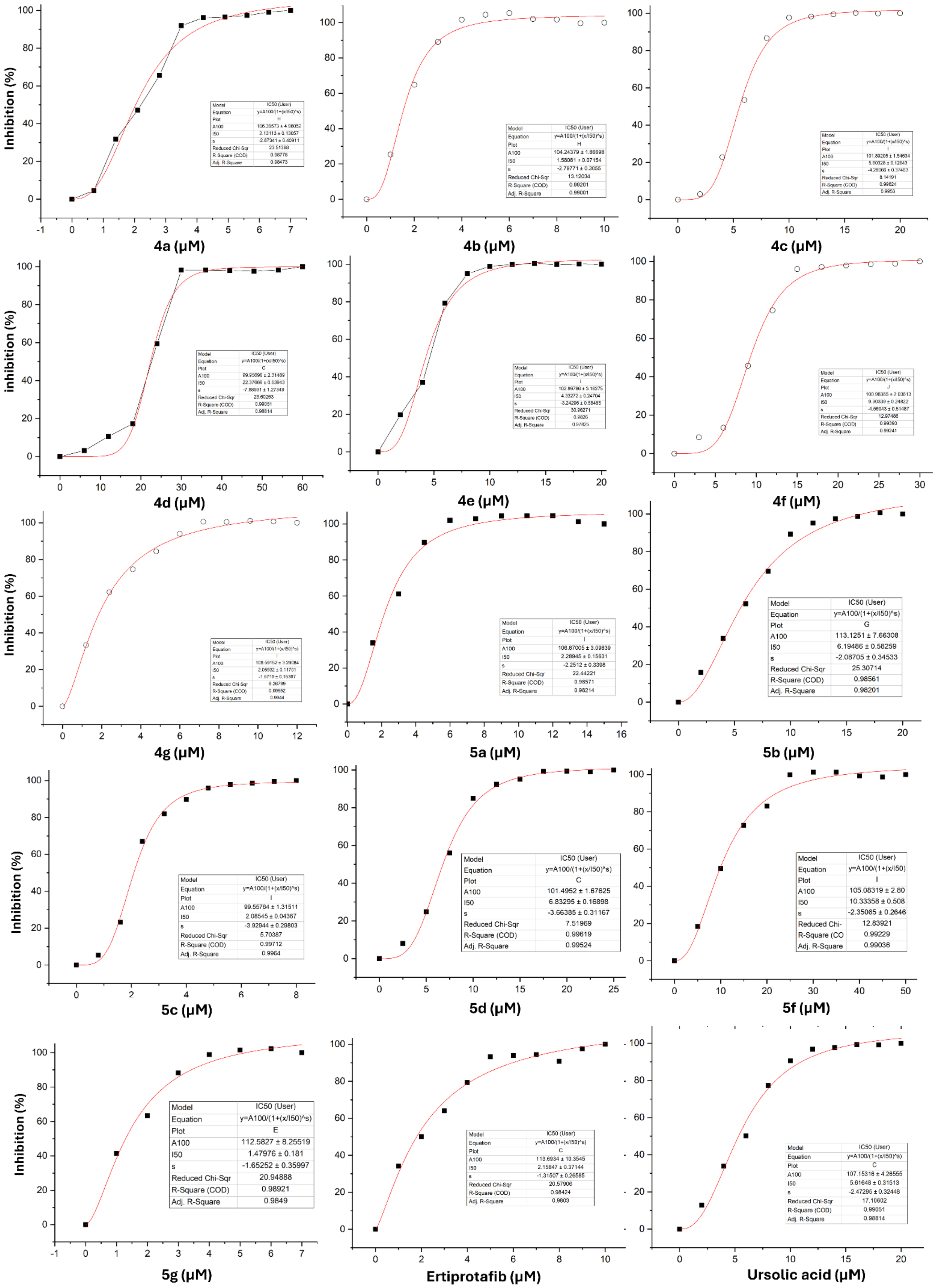

Figure S43. IC_{50} values for compounds 4a-5b against *h*$PTP1B_{1-400}$.

Table S1. Docking scores of GA derivatives against $PTP1B_{1-400}$-pNPP complex

Compound	Site 1				Site 2				Site 3			
	kcal/mol[a]	kcal/mol[b]	CS[c]	GS[d]	kcal/mol[a]	kcal/mol[b]	CP[c]	GS[d]	kcal/mol[a]	kcal/mol[b]	CP[c]	GS[d]
4a	-10.40	-10.32	50.33	47.13	-10.80	-9.73	50.81	43.48	-10.81	-9.51	59.51	36.81
4b	-10.36	-10.06	54.01	49.32	-10.85	-9.28	55.30	45.77	-10.81	-9.36	60.91	38.14
4e	-9.73	-10.41	49.02	48.99	-10.92	-8.98	44.58	55.71	-10.29	-9.25	48.09	39.18
4g	-10.90	-10.83	59.45	41.0	-9.46	-9.62	40.94	54.77	-9.86	-9.18	49.97	32.77
5g	-10.39	-10.83	50.96	7.88	-10.18	-9.53	45.93	5.79	-9.57	-8.72	48.16	25.93

[a]Binding energy values were retrieved from AutoDock; [b]binding energy values were retrieved from Vina; [c]CHEMPLP fitness score and [d]GoldScore values were retrieved from GOLD.

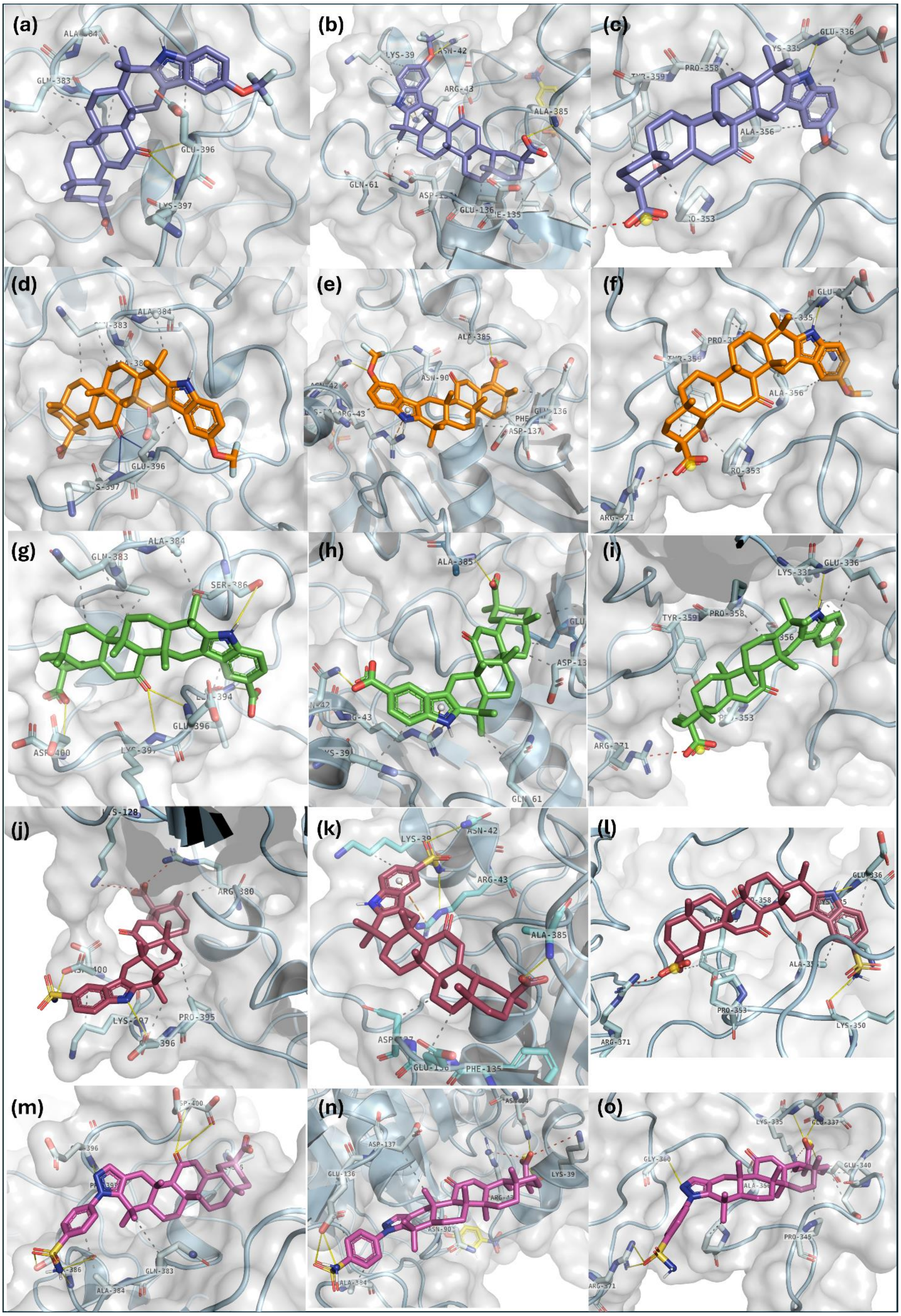

Figure S44. Predicted binding modes of GA derivatives **4a** (blue), **4b** (orange), **4e** (green), **4g** (raspberry), and **5g** (magenta), after docking against PTP1B$_{1\text{-}400}$-pNPP complex (pale cyan with gray surface). The pNPP substrate is highlighted in yellow. Predicted binding mode of **4a** within Site 1 (**a**), site 2 (**b**) and Site 3 (**c**). Predicted binding mode of **4b** within Site 1 (**d**), site 2 (**e**) and Site 3 (**f**). Predicted binding mode of **4e** within Site 1 (**g**), site 2 (**h**) and Site 3 (**i**). Predicted binding mode of **4g** within Site 1 (**j**), site 2 (**k**) and Site 3 (**l**). Predicted binding mode of **5g** within Site 1 (**m**), site 2 (**n**) and Site 3 (**o**). Solid yellow lines indicate hydrogen bond interactions, while red, orange, and gray dotted lines represent salt bridge interactions, π-cation interactions, and hydrophobic contacts, respectively.

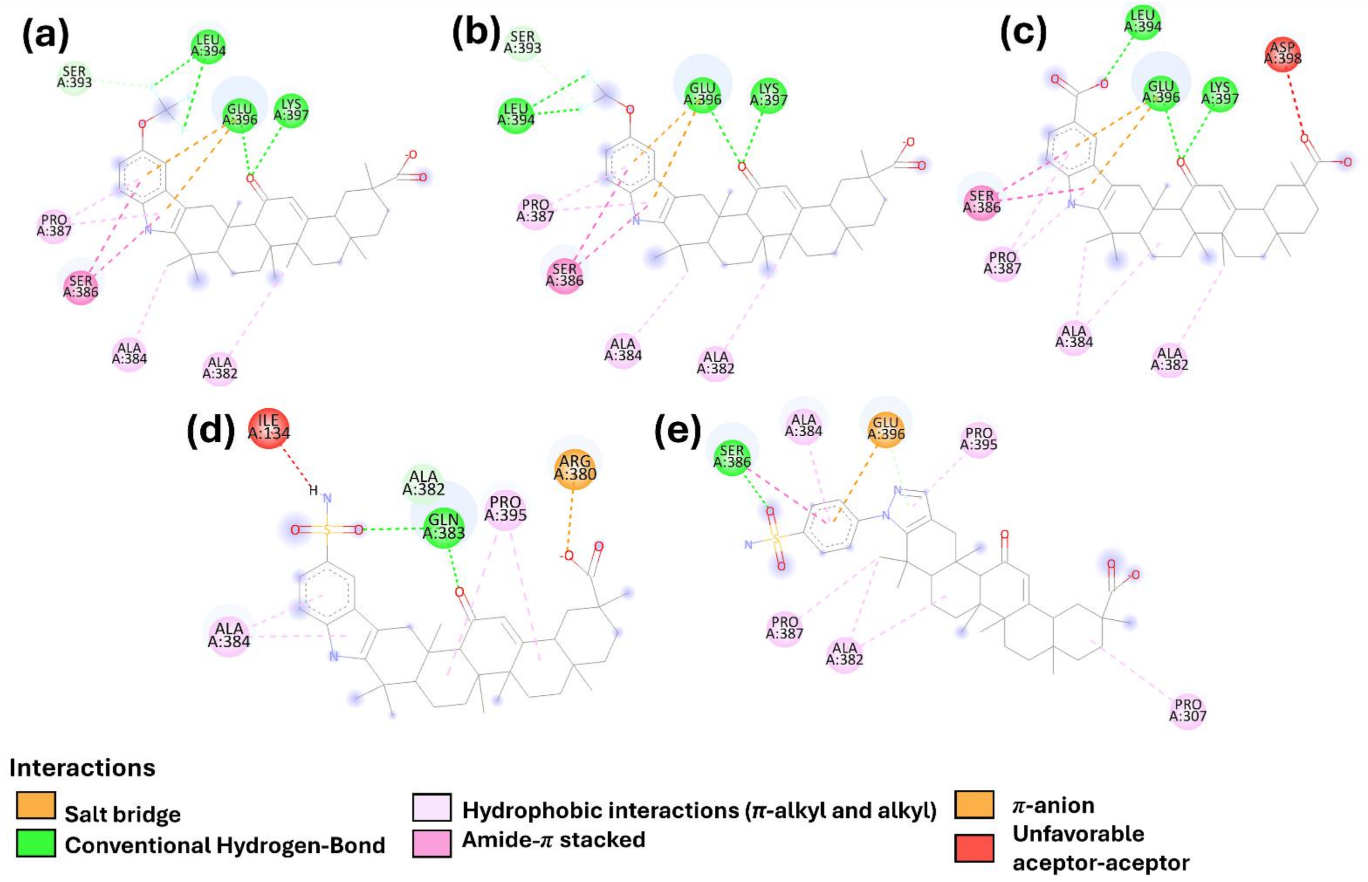

Figure S45. 2D diagram interaction of compounds 4a (a), 4b (b), 4e (c), 4g (d) and 5g (e) within the site 1 of PTP1B$_{1\text{-}400}$-pNPP complex.

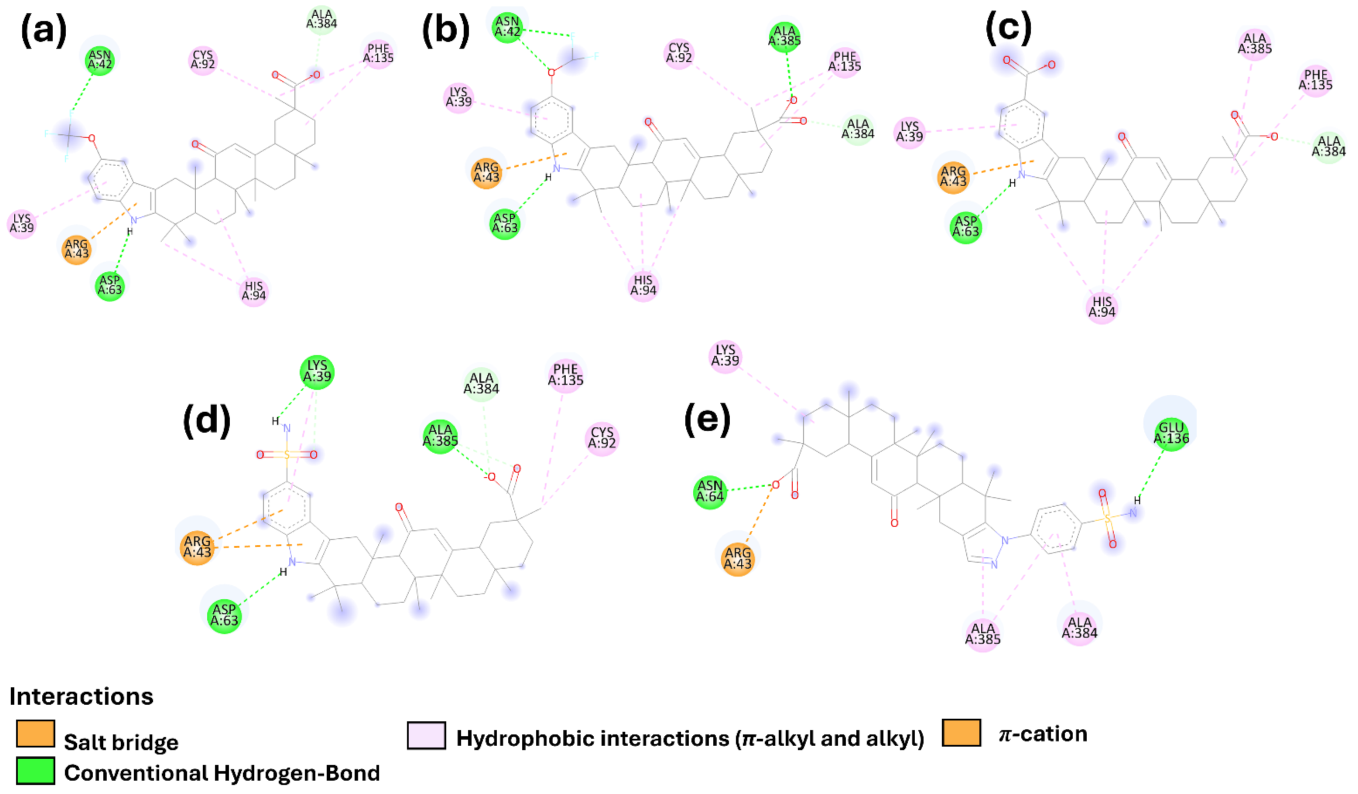

Figure S46. 2D diagram interaction of compounds 4a (a), 4b (b), 4e (c), 4g (d) and 5g (e) within the site 2 of PTP1B$_{1\text{-}400}$-pNPP complex.

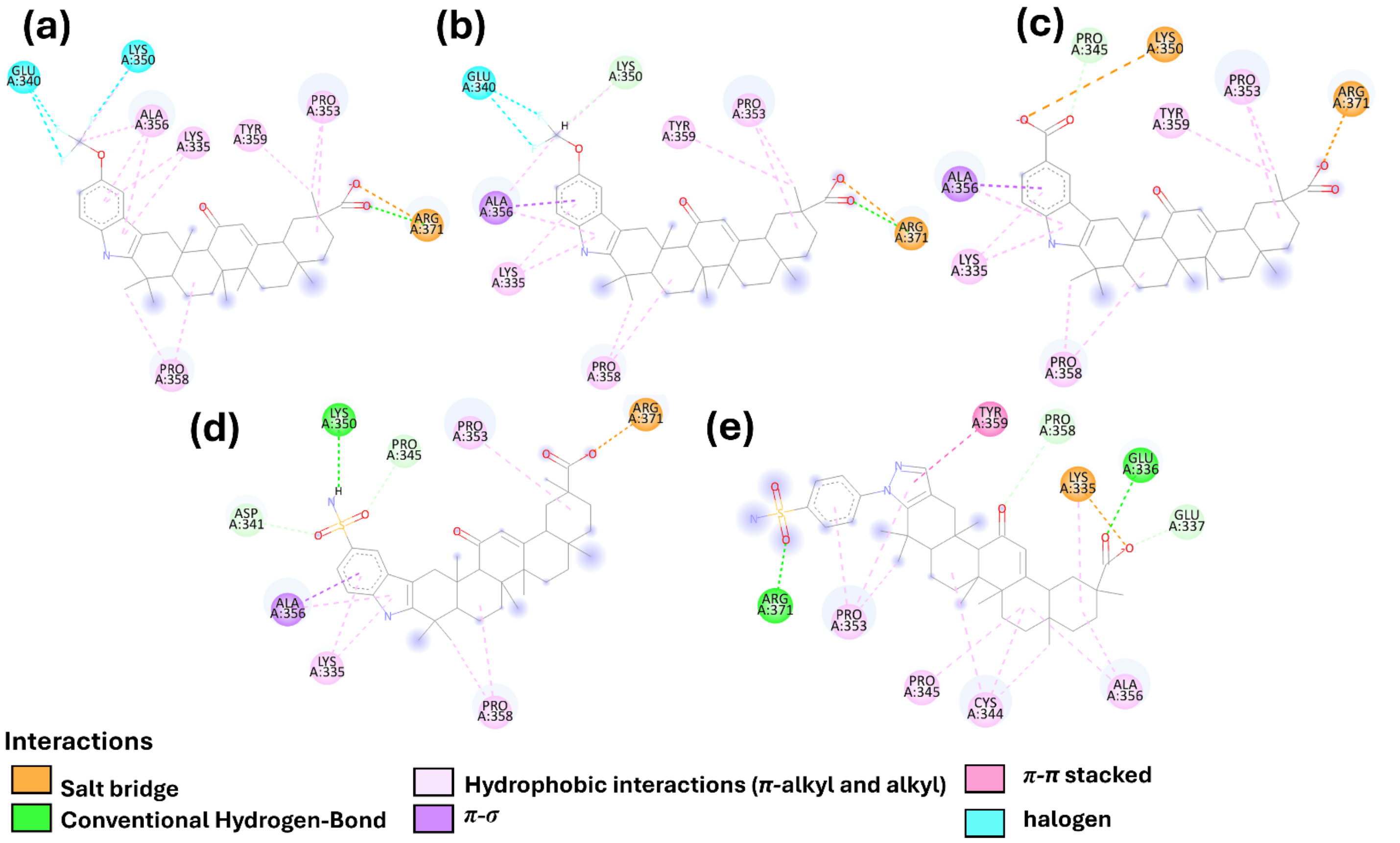

Figure S47. 2D diagram interaction of compounds 4a (a), 4b (b), 4e (c), 4g (d) and 5g (e) within the site 3 of PTP1B$_{1-400}$-pNPP complex.

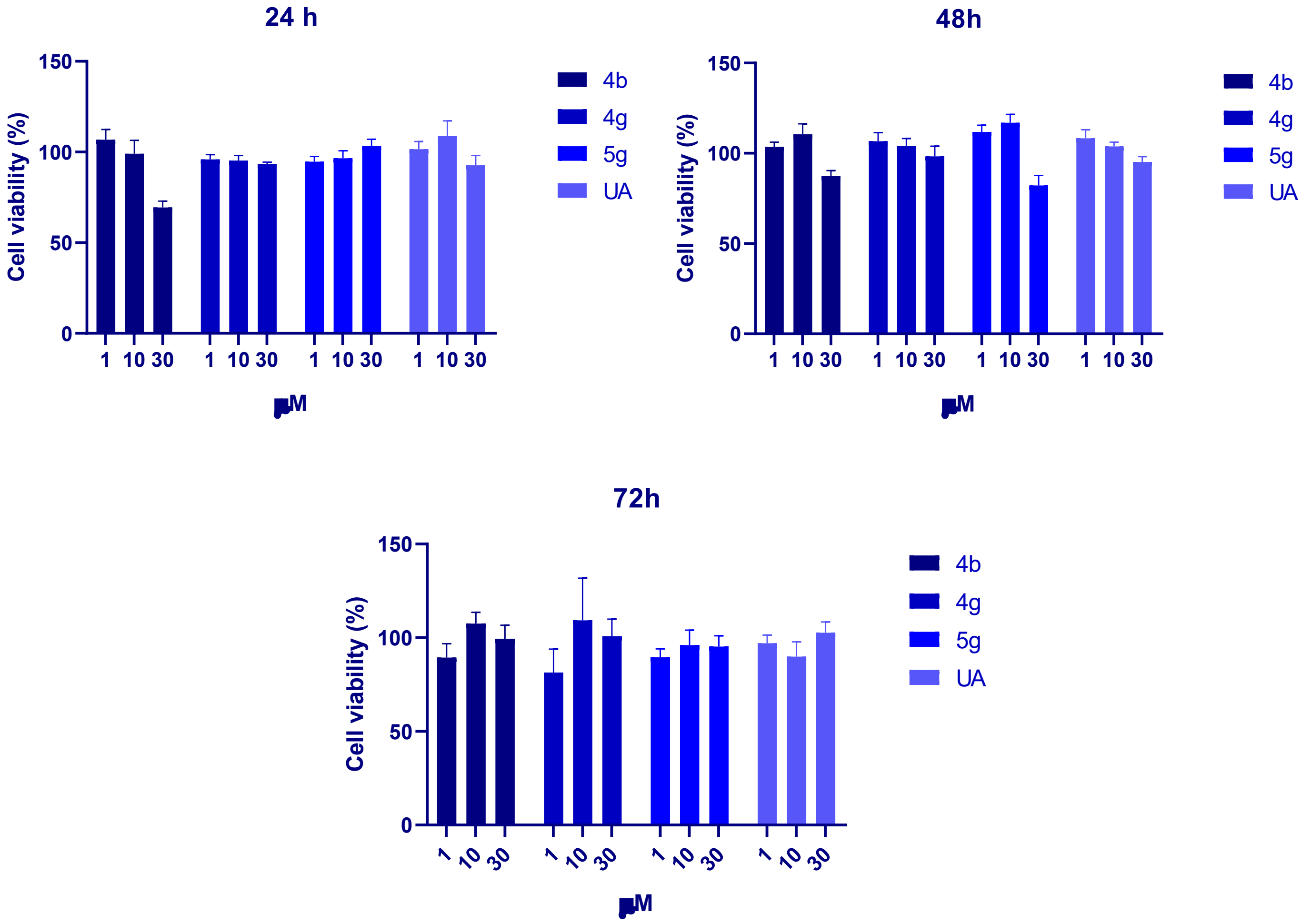

Figure S48. Cell viability assessment by crystal violet staining. HepG2 cell viability was evaluated after treatment with the respective compounds at concentrations of 1, 10, and 30 µM for 24, 48 and 72 hours. Bar graphs represent mean ± SEM. No significant differences were detected using ANOVA.

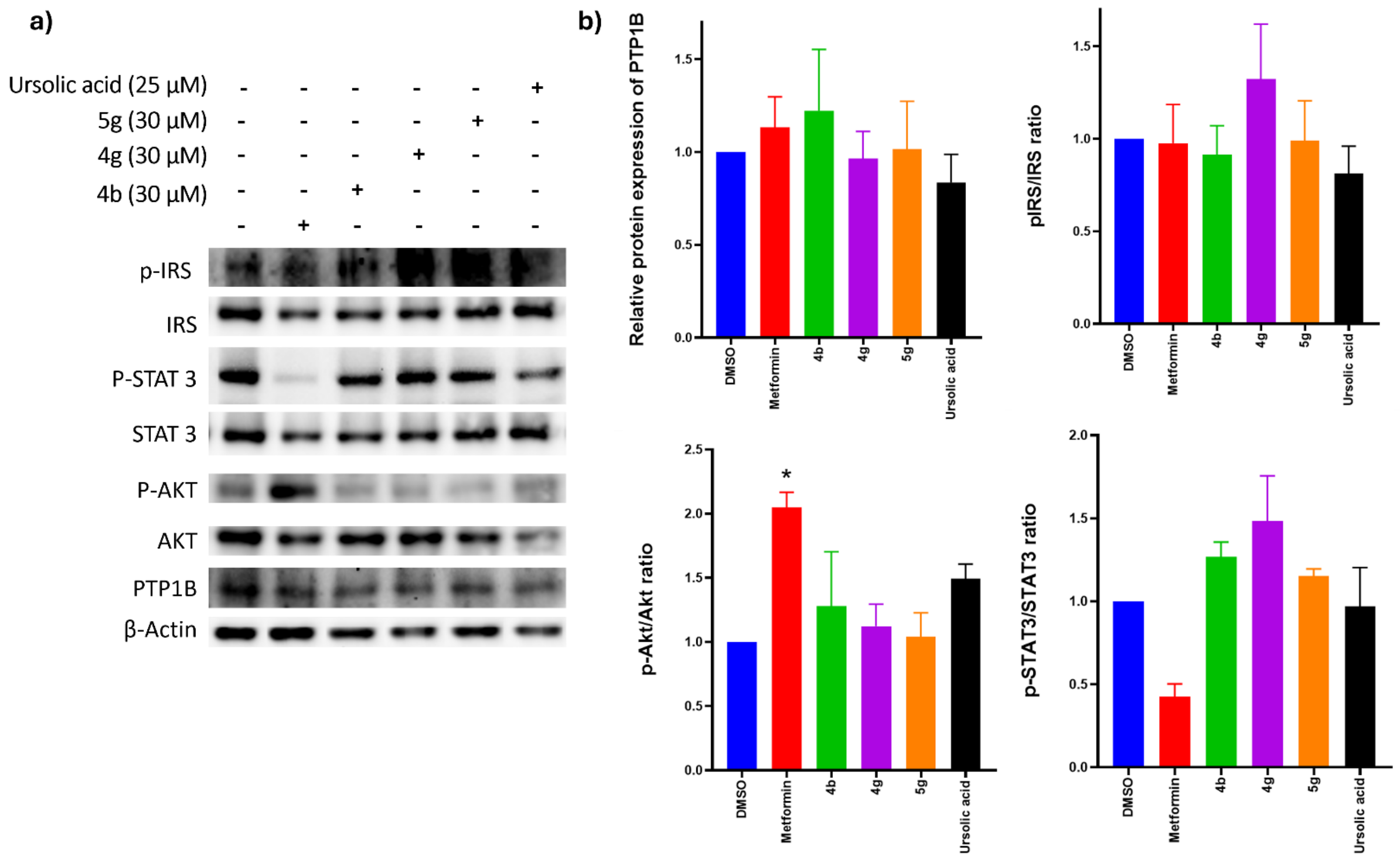

Figure S49. Effects 4b, 4g and 5g, metformin and ursolic acid on the levels of PTP1B, p-AKT, p-IRS1 and p-STAT3 in HepG2 cells. **a**) Western blotting representative analysis (n=3) of PTP1B, p-AKT, p-IRS1 and p-STAT3 levels of HepG2 cells treated with the compounds at 24 h of treatment. β-actin was employed as loading control. b) Densitometric analysis of western blot images employing ImageJ analysis software. Bar graphs represent the mean ± SEM of normalized values. The control levels were set to 1. Asterisks indicate statistical significance according to the Dunn test. $p^* < 0.05$

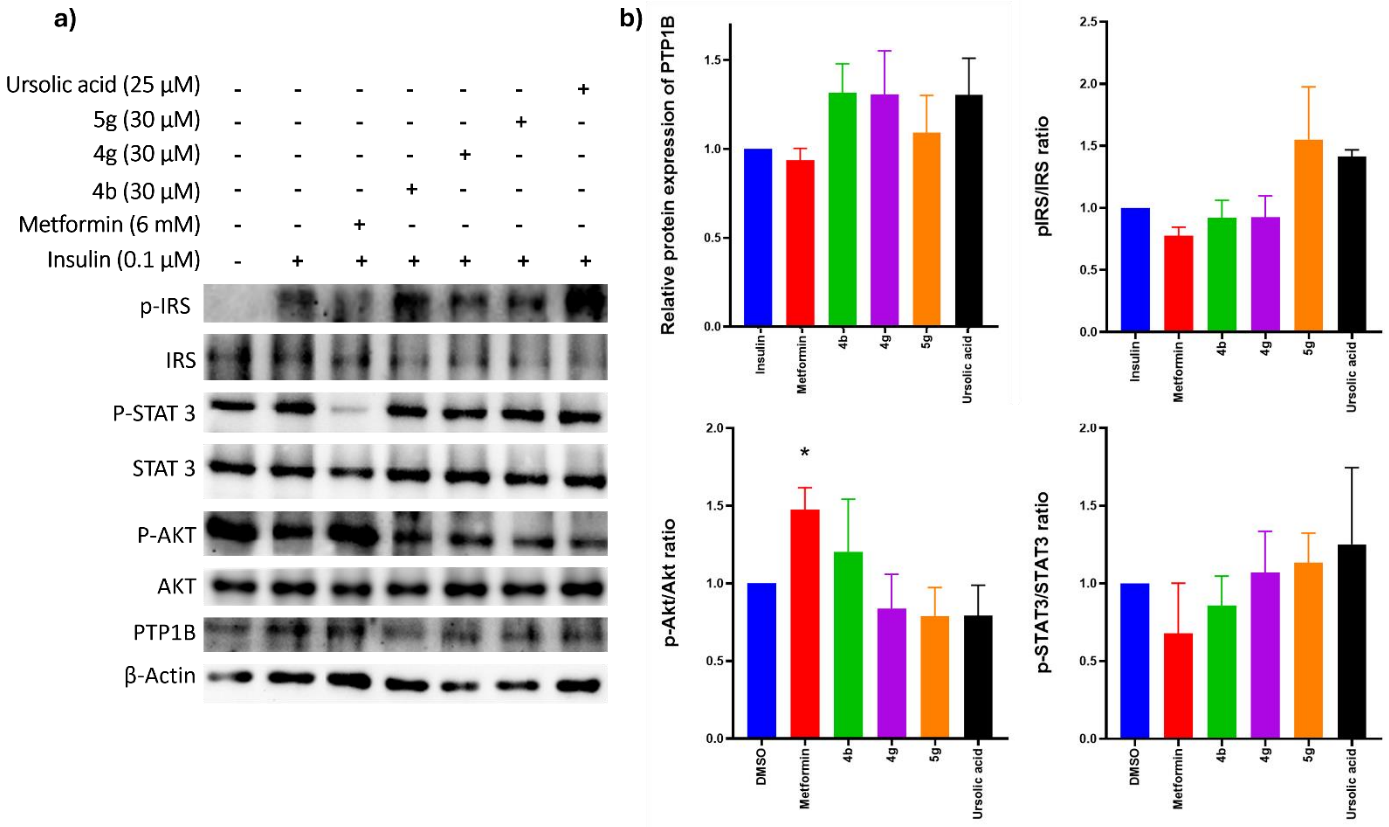

Figure S50. Effects 4b, 4g and 5g, metformin and ursolic acid on the levels of PTP1B, p-AKT, p-IRS1 and p-STAT3 in an insulin resistance model in HepG2 cells. **a**) Western blotting representative analysis (n=3) of PTP1B, p-AKT, p-IRS1 and p-STAT3 levels of HepG2 cells treated with the compounds at 24 h of treatment. β-actin was employed as loading control. b) Densitometric analysis of western blot images employing ImageJ analysis software. Bar graphs represent the mean ± SEM of normalized values. The control levels were set to 1. Asterisks indicate statistical significance according to the Dunn test. $p^* < 0.05$.

Table S2. Purity of compounds **4a-5g** determined by ^{1}H-qNMR using DSS as internal calibrant (IC)

Cmpd	m_{IC}	m_S	MW_{IC}	MW_t	P_{IC}	Int_{IC}	n_{IC}	Int_{t1}	n_{t1}	Int_{t2}	n_{t2}	Int_{t3}	n_{t3}	P(%)±S.D.
4a	2.2	18.6	218.33	625.35	97	2.9918	9	1.0084	1	1.0037	1	1.0059	1	95.9±0.2
4b	2.2	17.8	218.33	607.35	97	3.1410	9	1.0254	1	1.0291	1	1.0190	1	97.9±0.5
4c	2.2	15.2	218.33	641.32	97	4.9048	9	1.0622	1	1.0324	1	1.0662	1	98.0±0.4
4d	2.0	16.8	218.33	566.36	97	3.0068	9	2.1185	2	1.0764	1	1.0736	1	95.9±0.8
4e	0.9	4.5	218.33	585.35	97	4.6085	9	1.0227	1	1.051	1	1.0240	1	96.8±0.5
4f	3.2	15.9	218.33	584.36	97	1.1452	2	1.0200	1	1.0259	1	1.0280	1	93.5±0.4
4g	2.6	16.9	218.33	620.32	97	4.7791	9	1.0354	1	1.0307	1	1.0299	1	95.3±0.6
5a	2.0	14.1	218.33	654.35	97	3.9567	9	1.0072	1	1.0092	1	1.0080	1	94.6±0.1
5b	2.1	15.7	218.33	634.35	97	3.6344	9	0.9900	1	1.0024	1	0.9929	3	92.9±0.6
5c	1.1	3.2	218.33	668.33	97	9.8097	9	2.0763	2	2.0801	2	1.0457	1	97.6±0.4
5d	2.0	14.5	218.33	593.37	97	3.5723	9	2.0374	2	2.0137	2	1.0101	1	92.7±0.6
5e	2.2	16.2	218.33	613.35	97	3.4669	9	2.0313	2	2.0050	2	0.9999	1	96.5±0.8
5f	2.4	16.7	218.33	611.38	97	3.7574	9	0.9982	1	1.0102	1	1.0091	1	94.1±0.6
5g	2.0	20.0	218.33	647.35	97	2.9798	9	1.064	1	1.0773	1	1.0639	1	92.8±0.7

The purity (P) was calculated as follows:

$$P(\%) = \frac{n_{IC} * Int_{tn} * MW_t * m_{IC}}{n_{tn} * Int_{IC} * MW_{IC} * m_S} * P_{IC}$$

Where: *mIC = weight (mass in mg) of the internal calibrant (IC)*

mS = weight mass of the sample (mg)

Int_{IC} = area (integral) of the IC resonance signal being used for quantification

Int_{tn}= area (integral) of the target analyte (t) for each resonance signal (n) being used for quantification

n_{IC} = number of protons that give rise to Int_{IC}

n_{tn} = number of protons of the target analyte that give rise to Int_{tn}

MW_{IC} = molecular weight of the internal calibrant

MW_t = molecular weight of the target analyte

P_{IC} = purity of the internal calibrant, as percent value

Equation 1 was used to obtain enzyme kinetic parameters by fitting data to the Michaelis-Menten model (Origin Pro 2018 (64 bit) SR1)

$$y - \frac{V_{max}\, i}{K_m + i} \qquad \textbf{E1}$$

The equations defined for the following non-linear inhibition models were used to determine the mechanism of PTP1B inhibition: competitive (E2), non-competitive (E3), uncompetitive (E4), and mixed (E5) (Origin Pro 2018 (64 bit) SR1)

$$y = \frac{V_{max}\,(X)}{K_m\left(1+\left(\frac{i}{K_i}\right)\right) + x} \qquad \textbf{E2}$$

$$y = V_{max}\,\frac{x^{nh}}{\left(1+\left(\frac{i}{K_i}\right)\right)\left(x_{05}nh\ \right) + \left(1+\left(\frac{i}{K_i}\right)\right)\left(x_{05}\, nh\right)} \qquad \textbf{E3}$$

$$y = \frac{V_{max}\,(x)}{\left((K_m)/\left(\frac{1+i}{K_I}\right)\right) + x} \qquad \textbf{E4}$$

$$y = \frac{\left((V_{max}\,(x))/\left(\frac{1+i}{\alpha K_I}\right)\right)}{x + K_m\left(\left(\frac{1+i}{K_i}\right)/\left(\frac{1+i}{\alpha K_I}\right)\right)} \qquad \textbf{E5}$$

V_{max} is the maximum velocity, x is the substrate concentration, i is the inhibitor concentration, nh is the number of Hill. K_i is the inhibition constant, and K_m is the Michaelis constant.
